# Differential Diagnosis of Early-Stage Pulmonary Sarcoidosis With Normal Serum Angiotensin-Converting Enzyme Activity Using Peripheral Blood Indicators: Machine Learning Models Development and Validation

**DOI:** 10.2196/86639

**Published:** 2026-10-26

**Authors:** Lixiuyun Wang, Yafei Tang, Jun Ma, Shuqi Wang, Guoqiang Yang, Jin Huang, Qiuhong Li, Yonghong Feng

**Affiliations:** 1 Shanghai Key Laboratory of Tuberculosis Shanghai Pulmonary Hospital, Tongji University School of Medicine Shanghai China; 2 School of Public Health, the Key Laboratory of Environmental Pollution Monitoring and Disease Control, Ministry of Education, Guizhou Medical University Guiyang China; 3 Puxing Community Health Center, Pudong New District Shanghai China; 4 Department of Respiratory Medicine Shanghai Pulmonary Hospital, Tongji University School of Medicine Shanghai China

**Keywords:** pulmonary sarcoidosis, machine learning, diagnostic model, serum angiotensin-converting enzyme, sex stratification

## Abstract

**Background:**

Early-stage pulmonary sarcoidosis with serum angiotensin-converting enzyme (sACE) activity within the normal range is difficult to distinguish from other pulmonary diseases with similar radiographic characteristics.

**Objective:**

This study aimed to develop and validate machine learning (ML)–based diagnostic models for differentiating early-stage pulmonary sarcoidosis using indices from routine peripheral blood tests.

**Methods:**

From May 2014 to May 2021, a total of 1142 patients with atypical initial pulmonary imaging findings were enrolled. After applying strict inclusion and exclusion criteria, 420 cases were divided into sarcoidosis and nonsarcoidosis groups (including mediastinal lymph node tuberculosis [MLNT], lung cancer, lymphoma, and pneumoconiosis), then stratified by sex and randomly split (7:3) into training and validation sets. Features were selected via univariate analysis (*P*<.10), the Boruta algorithm, and least absolute shrinkage and selection operator regression. Six ML algorithms—logistic regression (LR), random forest, support vector machine (SVM), extreme gradient boosting, k‑nearest neighbor, and naïve Bayes—were compared using area under the curve (AUC), accuracy, sensitivity, specificity, *F*_1_-score, Brier score, calibration assessment, and decision curve analysis. Shapley Additive Explanations was used for interpretability. The selected models were deployed as an interactive Shiny web application.

**Results:**

After stratification, the male group comprised 224 cases (sarcoidosis n=42; MLNT: n=39; lung cancer: n=106; lymphoma: n=9; pneumoconiosis: n=28), and the female group comprised 196 cases (sarcoidosis: n=100; MLNT: n=39; lung cancer: n=49; lymphoma: n=4; pneumoconiosis: n=4). Models were selected based on comprehensive evaluation of multiple performance metrics, including AUC, accuracy, specificity, *F*_1_-score, and Brier score. The selected model for the male group was SVM, incorporating the following indices: age, lymphocyte count (Lym#), red cell distribution width standard deviation, D-dimer, sACE, cluster of differentiation 4-positive T cells to cluster of differentiation 8-positive T cells ratio (CD4^+^/CD8^+^), erythrocyte sedimentation rate, triglyceride to high-density lipoprotein cholesterol ratio (TG/HDLC), basophil count, and total protein. The selected model for the female group was LR, using Lym#, sACE, total cholesterol, TG/HDLC, glucose, CD3^+^%, and CD8^+^%. The validation set results showed AUCs of 0.896 (95% CI 0.746-1.000) and 0.762 (95% CI 0.632-0.892), sensitivities of 0.700 (95% CI 0.397-0.892) and 0.679 (95% CI 0.493-0.821), and specificities of 0.942 (95% CI 0.844-0.980) and 0.815 (95% CI 0.633-0.918), respectively.

**Conclusions:**

Based on sex‑stratified strategies, the models demonstrated preliminary discriminative ability for differentiating early‑stage pulmonary sarcoidosis with normal sACE from other pulmonary diseases. However, the wide CIs preclude definitive conclusions about clinical performance, and external validation in larger cohorts is needed.

## Introduction

Sarcoidosis is a systemic granulomatous disease characterized by the formation of noncaseating epithelioid cell granulomas with unknown etiology. It can affect multiple organs throughout the body, with the lungs being the most frequently involved site [1-3]. According to the Scadding staging system [4], sarcoidosis is classified into 5 stages (0 through IV). The radiological findings in stages 0, I, and II are atypical, often with bilateral hilar or/and mediastinal lymphadenopathy with (stage II) or without (stages 0 and I) infiltration in lungs, which are also presentations of pulmonary diseases like lung cancer, smear-negative mediastinal lymph node tuberculosis (MLNT) [5], pneumoconiosis, and lymphoma, making diagnosis challenging [1,6]. Without timely intervention, approximately 25% of patients may progress to chronic or progressive disease [7], even to advanced irreversibly fibrotic stage IV, with increased mortality due to respiratory failure, infection, vital organ involvement, or comorbidities [8-10]. Therefore, early diagnosis and treatment of pulmonary sarcoidosis are of paramount importance.

The diagnosis of sarcoidosis remains one of exclusion. The World Association of Sarcoidosis and Other Granulomatous Disorders first established diagnostic criteria for sarcoidosis in 1999 [11] and updated its organ assessment tool in 2014 [12]. Based on this tool, Bickett et al [5] developed 2 sarcoidosis diagnostic scores (SDS): clinical and biopsy scores. The SDS clinical score (with a threshold of ≥3) demonstrated a sensitivity of 90.6% and a specificity of 88.5% [5]. However, a subsequent multinational, multicenter validation study revealed its limited discriminatory power in distinguishing noninfectious granulomatous diseases, with an area under the curve (AUC) of 0.684 (95% CI 0.602-0.766). Furthermore, as the patient evaluation process lacks standardization, there is heterogeneity in the evaluation results among different physicians [13].

Serum angiotensin-converting enzyme (sACE) activity is often elevated in sarcoidosis and is regarded as a potential biomarker for evaluation of activity and diagnosis of sarcoidosis. However, it was reported that 24%-60% of patients with sarcoidosis do not present with elevated sACE levels [14-16]. These false-negative sACE results substantially increase diagnostic complexity.

In recent years, machine learning (ML) has been widely adopted in clinical research due to its strengths in handling complex data patterns [17-19]. Developing reliable ML diagnosis models for sarcoidosis could potentially become one of the approaches to addressing the pain points in clinical diagnosis, as recommended by the American Thoracic Society [20].

Previous studies [21,22] have reported significant sex differences in the incidence of sarcoidosis, with a higher incidence rate in the female group than in the male group. The peak age at diagnosis is 50 years and older for the female group and approximately 40 years for the male group. Accordingly, we adopted a sex-stratified approach in our ML modeling.

The purpose of this study was to construct an interpretable ML-based differential diagnosis model for pulmonary sarcoidosis using peripheral blood indicators and a sex-stratified strategy. By applying the Shapley Additive Explanations (SHAP) method to interpret the model and visualize the contribution of individual variables to diagnostic outcomes [23], we aimed to develop a clinical assessment tool that supports early diagnosis and intervention of pulmonary sarcoidosis, particularly for patients with sACE levels within the normal range.

## Methods

### Study Population

This study used data from the electronic medical record system of Shanghai Pulmonary Hospital, a tertiary Grade A hospital. We enrolled patients with pulmonary sarcoidosis (stage I and II) and nonsarcoidosis diagnoses (including MLNT, lung cancer, pneumoconiosis, and lymphoma) hospitalized between May 2014 and May 2021, all exhibiting atypical imaging features upon their first admission.

### Data Collection and Processing

#### Data Collection

Patient demographic characteristics (age and sex), clinical diagnoses, pathological diagnoses, and initial peripheral blood test data from the first admission were extracted from the hospital’s electronic medical record system. The peripheral blood indicators included a complete blood count (red blood cells, neutrophils, and lymphocytes), humoral immunity markers (immunoglobulin G, immunoglobulin M, and immunoglobulin A), biochemical tests (total protein [TP] and albumin), cellular immunity markers (cluster of differentiation [CD]3^+^% and CD4^+^/CD8^+^ ratio), lipid profile (total cholesterol [TC], triglycerides, apolipoprotein A, and apolipoprotein E), and coagulation parameters (prothrombin time, activated partial thromboplastin time, antithrombin III, thrombin time, and fibrinogen). All variables and their reference ranges are detailed in Table S1 in Multimedia Appendix 1. Additional characteristics included symptoms, computed tomography (CT) findings, and comorbidities.

#### Inclusion and Exclusion Criteria

All cases presented with atypical imaging findings of the diagnosed diseases (no definitive diagnosis) at initial assessment were independently assessed by 2 senior physicians (JM and QL) from the departments of tuberculosis and respiratory medicine. Only those with consistent judgments from both physicians were selected according to the disease-specific criteria:

- Criteria for MLNT case selection: Cases in pathological stages I and II, which present with atypical features on CT, were selected [24]. While extensively calcified lymph nodes were easily distinguishable, those with partial calcification pose a diagnostic challenge. This study specifically enrolled cases with partial calcification who tested negative for *Mycobacterium tuberculosis* on smear and molecular tests (*M tuberculosis* DNA or RNA) at initial admission but subsequently tested positive on *M tuberculosis* culture or had 3 consecutive positive smears with *M tuberculosis* species identification after at least 1 month of follow-up.
- Criteria for pulmonary sarcoidosis case selection: Stage I and II cases that met international consensus diagnostic criteria with atypical manifestations were chosen. These included cases demonstrating amorphous or partial calcification in lymph nodes.
- Criteria for pneumoconiosis case selection: Given the inherent difficulty in differentiating pneumoconiosis from other diseases based solely on imaging, the study enrolled cases without a definitive preadmission occupational disease diagnosis. All cases received a confirmed diagnosis of pneumoconiosis during follow-up.
- Criteria for lung cancer case selection: The selected cases exhibited extensive pulmonary infiltrates and significant lymph node involvement, with a pathologically confirmed diagnosis during the follow-up period.
- Criteria for lymphoma (particularly Hodgkin lymphoma and non-Hodgkin lymphoma) case selection: The selected cases demonstrated mild to moderate homogeneous enhancement and minimal confluence on imaging, with evidence of pulmonary lymphangitic infiltration on contrast-enhanced CT, and with confirmation of lymphoma diagnosis by histopathology during follow-up.

Exclusion criteria (meeting any of the following):

- Treatment interference: Patients who had received antituberculosis therapy, antitumor therapy, and corticosteroid treatment; who had a confirmed diagnosis before admission; or who had used antibiotics (other than cephalosporins) or angiotensin-converting enzyme inhibitors within 2 weeks prior to admission.
- Abnormal sACE: sACE levels exceeding the normal (>70 IU/L in this study).
- As the lipid variables and the CD4^+^/CD8^+^ have demonstrated considerable value in sarcoidosis diagnosis [25-27], cases with missing records for either lipid variables or the CD4^+^/CD8^+^ ratio were excluded.
- Comorbidities: Concurrent diagnosis with any other disease included in the study groups.
- Cases with other diseases, such as aspergillosis or chronic beryllium disease, that have similar imaging presentations and can be easily diagnosed with characteristic laboratory tests were also excluded.

Based on the inclusion and exclusion criteria, the study included a total of 224 male cases (pulmonary sarcoidosis: n=42, MLNT: n=39, lung cancer: n=106, lymphoma: n=9, and pneumoconiosis: n=28) and 196 female cases (pulmonary sarcoidosis: n=100, MLNT: n=39, lung cancer: n=49, lymphoma: n=4, and pneumoconiosis: n=4). A flowchart outlining the study process is provided in Figure 1.

**Figure 1:**
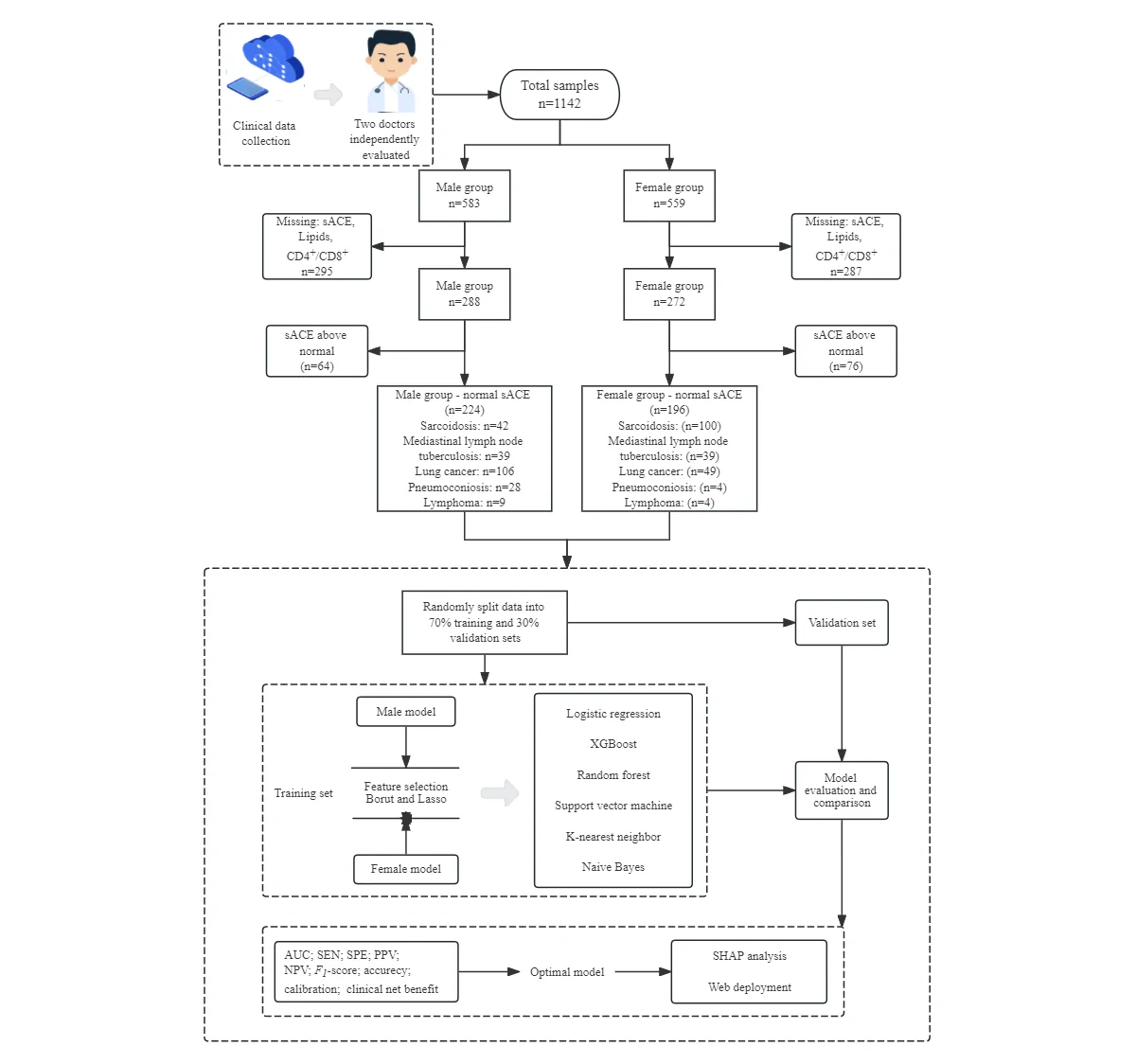
Flowchart of data collection, processing, and analysis. AUC: area under the curve; CD: cluster of differentiation; sACE: serum angiotensin-converting enzyme; SEN: sensitivity; SHAP: Shapley Additive Explanations; SPE: specificity; XGBoost: extreme gradient boosting.

### Model Development and Comparison

The dataset was randomly split into a training set and a validation set in a 7:3 ratio. The parameters for the data preprocessing steps on the validation set (including missing value imputation and standardization) were all derived from the training set to prevent data leakage and ensure an unbiased evaluation of the validation results. Feature selection was performed on the training set. The standardized formula was: 



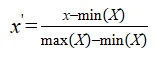



where min(*X*) denoted the minimum value of the data and max(*X*) denoted the maximum value of the data. Model variable selection was performed solely on the training set, and the selected features were fixed before any validation set evaluation, following the process: first, univariate analysis was performed to identify variables with a significance level of *P*<.10. These variables were then subjected to importance analysis using the Boruta algorithm, which rigorously identifies features that are genuinely associated with the target variable by iteratively comparing the importance of original features with that of randomly permuted shadow features. This allows us to retain variables that are statistically relevant to sarcoidosis while filtering out noise, providing a reliable initial feature set [28]. The variables obtained from the Boruta algorithm were subjected to feature compression via least absolute shrinkage and selection operator (LASSO) regression (implemented via the *glmnet* package). LASSO applies L1 regularization to further shrink the coefficient vector. Building on the Boruta output, LASSO eliminates redundant or multicollinear variables, resulting in a more parsimonious model.

The selected feature set from the training set was used to develop models using 6 ML algorithms: logistic regression (LR), random forest (RF), support vector machine (SVM), extreme gradient boosting (XGBoost), naïve Bayes (NB), and k-nearest neighbors (KNNs). To counteract the class imbalance issue in the male data (sarcoidosis cases: 42/224, 18.7%), we used a model weighting approach when modeling the male subgroup. The weight calculation formula is:



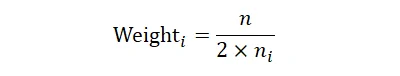



where *n* is the total sample size, and *n_i_* is the sample size for group *i*.

LR was implemented using the *stats* package, RF models were implemented using the *randomForest* package for the male cohort and the *ranger* package for the female cohort, SVM and NB using the *e1071* package, XGBoost using the *xgboost* package, and KNN using the *kknn* package. In the male group modeling, weight parameters were incorporated into the code. Specifically, the core of the KNN algorithm lies in its distance-weighted classification: for each test sample, the KNNs are identified based on Euclidean distance, and different weights are assigned according to their distances—closer neighbors receive higher weights. The final predicted class was determined by weighted voting. For the NB algorithm, weighting was achieved by adjusting the prior probabilities. Under class imbalance, setting prior probabilities inversely proportional to the class proportions enhances the algorithm’s ability to recognize the minority class. The validation set was reserved for model evaluation. To achieve optimal model performance while minimizing the risk of overfitting, hyperparameter tuning was performed using nested cross-validation and grid search.

Each model was comprehensively evaluated for its discrimination, calibration, and clinical net benefit (using decision curve analysis [DCA]). The discriminatory ability of the 6 models was assessed using the AUC, sensitivity, specificity, positive predictive value, negative predictive value, *F*_1_-score, and accuracy, while the DCA evaluated the practical utility and clinical application value of each model.

### Model Stability Analysis

We performed 10-fold cross-validation on the training set using the best model to demonstrate the model’s stability during training. Then, we additionally assessed the robustness and generalizability of the model in the validation cohort by evaluating its discriminative performance between sarcoidosis and each individual disease separately.

### Model Explanation and Web Deployment

SHAP is a method that ranks feature importance and interprets model outputs. It provides both global and local explanations for a model. Globally, it reveals overall feature-disease associations, while locally, it illustrates the reasoning behind individual predictions [17,23]. To facilitate clinical adoption and enhance understanding for clinicians, the final models were deployed as an interactive Shiny web application. By inputting a patient’s corresponding metric values, the application provides the probability score of the patient being diagnosed with pulmonary sarcoidosis and generates an individual force plot for case-specific interpretation.

### Statistical Analysis

Two independent data entry clerks performed double entry and proofreading to ensure accuracy and reliability. Except for lipid variables and the CD4^+^/CD8^+^ ratio, all other variables with missing values were imputed using the median method [29]. Specifically, the median calculated from the training set was used to impute missing values in the training set, and the same median value was then applied to the validation set to prevent data leakage. Categorical variables are presented as percentages and compared using the chi-square test. Normally distributed continuous data are expressed as mean (SD) and analyzed with the 2-sided *t* test, while nonnormally distributed data are summarized as median (IQR) and compared using the Mann-Whitney *U* test. The optimal cutoff value was established by maximizing the Youden index (sensitivity+specificity–1). Statistical significance was defined as a 2-sided *P* value <.05. All analyses, including ML model construction and SHAP value analysis, were performed using the R software (version 4.5.1; R Foundation for Statistical Computing). The R code used for the correlation analyses is provided in Multimedia Appendix 2 to facilitate reproducibility.

### Ethical Considerations

This study was conducted in accordance with the amended Declaration of Helsinki and the ethical guidelines of the institutional review board of Tongji University (project 221 approval K18-142). Clinical data were anonymized.

## Results

### Baseline Characteristics

The entire dataset included 224 male cases and 196 female cases. After the data were divided into training and validation sets, the male and female groups consisted of 162 cases and 141 cases in the training set, and 62 cases and 55 cases in the validation set, respectively. Among male patients with pulmonary sarcoidosis, the median age was 44.0 (IQR 39.0-49.8) years; among female patients, the median age was 53.0 (IQR 48.0-60.0) years. In nonsarcoidosis cases, the median age was 59.0 (IQR 46.0-66.0) years for the male group and 55.5 (IQR 45.0-64.2) years for the female group. A higher proportion of bilateral hilar lymphadenopathy was observed in patients with pulmonary sarcoidosis compared to patients without sarcoidosis in both male and female groups. Table 1 describes the demographic information for male and female groups in the entire dataset, as well as the variables included in the models. Tables S2 and S3 in Multimedia Appendix 1 summarize the missing value patterns, while Tables S4 and S5 in Multimedia Appendix 1 present the statistical results for all variables.

**Table 1:** Baseline demographic characteristics of male and female groups in the overall dataset and peripheral blood parameters included in the models.

				Male group								Female group						
				Nonsarcoidosis (n=182)		Sarcoidosis (n=42)		*P* value			Nonsarcoidosis (n=96)			Sarcoidosis (n=100)			*P* value	
**Disease category, n (%)**									<.001									<.001
	Mediastinal lymph node tuberculosis			39 (21.4)		0 (0)					39 (40.6)			0 (0)				
	Sarcoidosis			0 (0)		42 (100)					0 (0)			100 (100)				
	Lymphoma			9 (4.9)		0 (0)					4 (4.2)			0 (0)				
	Lung cancer			106 (58.2)		0 (0)					49 (51)			0 (0)				
	Pneumoconiosis			28 (15.4)		0 (0)					4 (4.17)			0 (0)				
**Demographic factors**																		
	Smoking, n (%)			91 (50)		13 (31)		.04			2 (2.1)			2 (2)			>.99	
	Drinking, n (%)			25 (13.7)		3 (7.1)		.36			0			0			—^a^	
	Age (years), median (IQR)			59.0 (46.0-66.0)		44.0 (39.0-49.8)		<.001			55.5 (45.0-64.2)			53.0 (48.0-60.0)			.48	
	Lymphocyte count (×10^9^/L), median (IQR)			1.61 (1.20-2.09)		1.35 (1.05-1.77)		.005			1.52 (1.22-1.91)			1.41 (1.14-1.66)			.02	
	Basophil count (×10^9^/L), median (IQR)			0.02 (0.01-0.03)		0.01 (0.01-0.02)		<.001			0.01 (0.01-0.03)			0.01 (0.01-0.02)			.48	
	Red cell distribution width standard deviation (fL), median (IQR)			42.6 (40.6-44.7)		40.8 (39.2-41.9)		<.001			41.8 (39.9-43.8)			41.4 (39.9-43.0)			.27	
	**Total protein** **(g/L)**							<.001									.09	
		Mean (SD)	63.7 (8.79)		69.6 (7.56)					—			—					
		Median (IQR)	—		—					67.0 (61.2-72.5)			68.6 (63.9-73.7)					
	D-dimer (ng/mL), median (IQR)			151 (84.5-350)		122 (76.0-162)		.007			177 (100-328)			138 (83.8-194)			.003	
	Glucose (mmol/L), median (IQR)			5.00 (4.60-5.54)		5.19 (5.00-5.53)		.06			5.00 (4.70-5.40)			5.40 (4.90-6.02)			.002	
	Serum angiotensin-converting enzyme (IU/L), median (IQR)			33.0 (23.0-45.8)		43.0 (35.5-54.0)		.001			37.0 (29.0-44.0)			48.0 (38.0-59.0)			<.001	
	**Total cholesterol** **(mmol/L)**							.001									.009	
		Mean (SD)	—		—					4.94 (0.97)			5.27 (0.80)					
		Median (IQR)	4.79 (4.02-5.34)		5.23 (4.88-5.63)					—			—					
	Triglyceride to high-density lipoprotein cholesterol ratio, median (IQR)			1.00 (0.67-1.52)		1.37 (0.98-1.93)		.003			0.88 (0.59-1.56)			1.32 (0.77-2.06)			.006	
	Erythrocyte sedimentation rate (mm/h), median (IQR)			31.3 (16.3-66.7)		18.7 (10.7-29.3)		<.001			25.5 (13.0-56.2)			24.0 (14.0-32.2)			.19	
	**Percentage of CD^b^3^+^ T cells (%)**							.69									.002	
		Mean (SD)	65.6 (11.5)		64.8 (12)					—			—					
		Median (IQR)	—		—					71.8 (62.7-76.5)			63.7 (57.8-72.1)					
	Percentage of CD 8^+^ T cells (%), median (IQR)			19.0 (14.8-25.5)		17.2 (11.6-25.6)		.24			20.2 (15.1-25.7)			15.8 (12.1-21.6)			<.001	
	CD4^+^ T cells to CD8^+^ T cells ratio, median (IQR)			1.73 (1.21-2.59)		2.14 (1.29-3.20)		.10			2.08 (1.43-2.67)			2.50 (1.74-3.36)			.003	
**Symptoms or complications, n (%)**																		
	Allergies			5 (2.7)		0 (0)		.59			6 (6.3)			0 (0)			.01	
	Hypertensive			37 (20.3)		3 (7.1)		.07			13 (13.5)			22 (22)			.17	
	Diabetes			17 (9.3)		4 (9.5)		>.99			7 (7.3)			7 (7)			>.99	
	Asthma			1 (0.5)		1 (2.4)		.34			1 (1)			2 (2)			>.99	
	Chronic bronchitis			6 (3.3)		1 (2.4)		>.99			2 (2.1)			1 (1)			.62	
	Bronchiectasis			14 (7.7)		3 (7.1)		>.99			2 (2.1)			13 (13)			.009	
	Cough			130 (71.4)		26 (61.9)		.31			58 (60.4)			73 (73)			.09	
	Coughing up phlegm			106 (58.2)		19 (45.2)		.18			42 (43.8)			56 (56)			.12	
	Arrhythmia			38 (20.9)		5 (11.9)		.27			11 (11.5)			9 (9)			.74	
	Fever			31 (17)		7 (16.7)		>.99			16 (16.7)			8 (8)			.10	
	Chest tightness			36 (19.8)		9 (21.4)		.98			17 (17.7)			24 (24)			.36	
	Fatigue			39 (21.4)		4 (9.5)		.12			12 (12.5)			13 (13)			>.99	
	Hemoptysis			35 (19.2)		3 (7.1)		.10			9 (9.4)			5 (5)			.36	
	Night sweats			14 (7.7)		3 (7.1)		>.99			7 (7.3)			6 (6)			.94	
	Chest pain			29 (15.9)		9 (21.4)		.53			13 (13.5)			9 (9)			.43	
	Dyspnea			37 (20.3)		6 (14.3)		.50			12 (12.5)			19 (19)			.29	
	Weight loss			8 (4.4)		1 (2.4)		>.99			3 (3.1)			7 (7)			.33	
	Pericardial effusion			14 (7.7)		0 (0)		.08			11 (11.5)			2 (2)			.02	
	Uterine fibroids			—		—		—			5 (5.2)			13 (13)			.10	
	Thyroid nodule			8 (4.4)		0 (0)		.36			6 (6.3)			10 (10)			.48	
	Subcutaneous nodule			0 (0)		3 (7.1)		.006			1 (1)			5 (5)			.21	
	Gallbladder			19 (10.4)		3 (7.1)		.77			12 (12.5)			19 (19)			.29	
	Kidney nodule			39 (21.4)		6 (14.3)		.41			14 (14.6)			22 (22)			.25	
	Liver nodule			49 (26.9)		9 (21.4)		.59			23 (24)			34 (34)			.16	
	Pleurale effusion			42 (23.1)		6 (14.3)		.30			20 (20.8)			7 (7)			.009	
**Imaging features**																		
	Cavitation, n (%)			15 (8.2)		0 (0)		.08			3 (3.1)			0 (0)			.12	
	Calcification, n (%)			17 (9.3)		3 (7.1)		>.99			9 (9.4)			3 (3)			.12	
	Number of lung field infiltrates, median (IQR)			2 (1.00-4.00)		3.00 (2.00-4.75)		.02			2.00 (1.00-4.00)			2.00 (2.00-4.00)			.17	
**Hilar lymphadenopathy type, n (%)**									<.001									<.001
	No enlargement			66 (36.3)		1 (2.4)					39 (40.6)			1 (1)				
	Bilateral enlargement			51 (28)		41 (97.6)					30 (31.3)			98 (98)				
	Unilateral enlargement			65 (35.7)		0 (0)					27 (28.1)			1 (1)				
**Lesion location, n (%)**																		
	Left upper lobe			27 (14.8)		4 (9.5)		.51			21 (21.9)			16 (16)			.38	
	Left lower lobe			27 (14.8)		3 (7.1)		.28			17 (17.7)			11 (11)			.25	
	Right upper lobe			59 (32.4)		2 (4.8)		.001			24 (25)			13 (13)			.05	
	Right lower lobe			42 (23.1)		4 (9.5)		.08			20 (20.8)			7 (7)			.009	
	Right middle lobe			10 (5.5)		2 (4.8)		>.99			10 (10.4)			7 (7)			.55	

^a^Not applicable.

^b^CD: cluster of differentiation.

### Model Development and Performance Comparison

Table 2 lists the detailed parameters for each model after tuning. Figure S1 in Multimedia Appendix 1 illustrates the variable selection process for the 2 models. In the female model, features corresponding to the minimum lambda value were selected, including lymphocyte count (Lym#), sACE, TC, triglyceride to high-density lipoprotein cholesterol ratio (TG/HDLC), glucose, CD3^+^, and CD8^+^. The features incorporated into the male model were age, Lym#, red cell distribution width standard deviation (RDW-SD), D-dimer, sACE, CD4^+^/CD8^+^, erythrocyte sedimentation rate (ESR), TG/HDLC, absolute basophil count (BASO#), and TP.

When evaluating the overall performance of the 6 ML models based on multiple performance metrics, including AUC, accuracy, specificity, *F*_1_-score, and Brier score, SVM performed best among the male models (training set: AUC 0.989, 95% CI 0.975-1.000; validation set: AUC 0.896, 95% CI 0.746-1.000), while LR performed best among the female models (training set: AUC 0.848, 95% CI 0.781-0.915; validation set: AUC 0.762, 95% CI 0.632-0.892). Figure 2 presents the receiver operating characteristic curves, calibration curves, and DCA curves for all 6 ML algorithms in the validation set. The corresponding results for the training set are provided in Figure S2 in Multimedia Appendix 1. The discrimination metrics for each model are detailed in Table 3 and Table S6 in Multimedia Appendix 1.

**Table 2:** Specific results of parameter tuning for male and female models.

		Hyperparameter tuning details
**Male models**		
	LR^a^	family=quasibinomial(link=“logit”)
	SVM^b^	kernel=“radial”, type=“C-classification”, gamma=0.1, cost=1
	XGBoost^c^	max_depth=“5”, eta=“0.1”, colsample_bytree=“0.8”, min_child_weight=“3”, subsample=“0.6”, objective=“binary:logistic”, nrounds=57
	RF^d^	ntree=100, mtry=1, nodesize=5
	KNN^e^	K=33, kernel=“triangular”
	NB^f^	laplace=1
**Female models**		
	LR	family=“binomial”
	SVM	kernel=“radial”, type=“C-classification”, gamma=0.01, cost=10
	XGBoost	max_depth=“2”, eta=“0.1”, subsample=“0.8”, colsample_bytree=“0.8”, min_child_weight=“5”, gamma=“0.5”, lambda=“1”, alpha=“0.5”
	RF	ntree=300, mtry=2, min.node.size=10
	KNN	K=25
	NB	laplace=1

^a^LR: logistic regression.

^b^SVM: support vector machine.

^c^XGBoost: extreme gradient boosting.

^d^RF: random forest.

^e^KNN: k-nearest neighbor.

^f^NB: naïve Bayes.

**Figure 2:**
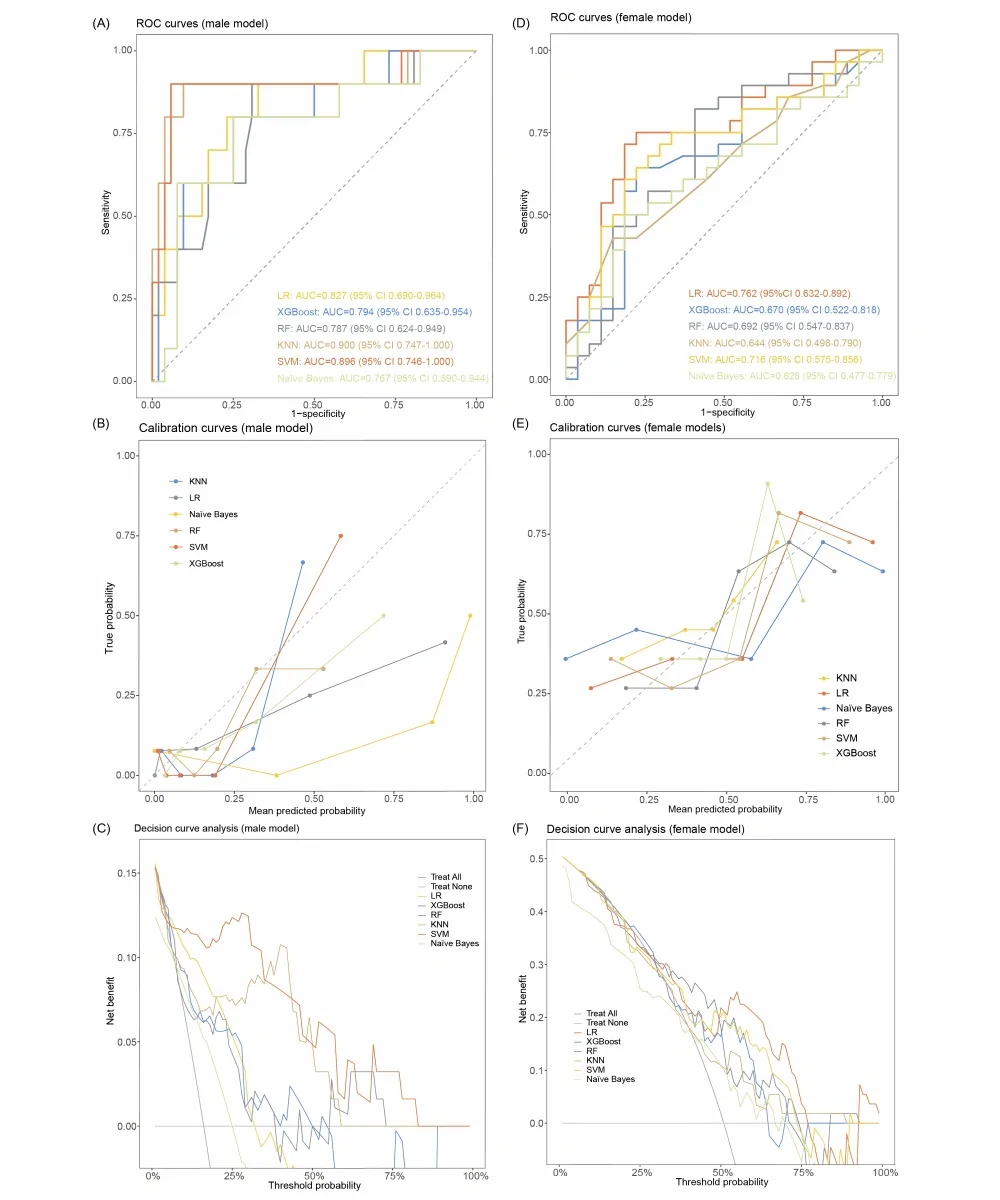
(A and D) ROC curves, (B and E) calibration plots, and (C and F) decision curve analysis of male and female models on the validation set. AUC: area under the curve; KNN: k-nearest neighbor; LR: logistic regression; RF: random forest; ROC: receiver operating characteristic; SVM: support vector machine; XGBoost: extreme gradient boosting.

**Table 3:** Diagnostic performance metrics of different machine learning algorithms for male and female models on the validation set.

		LR^a^	RF^b^	XGBoost^c^	KNN^d^	SVM^e^	NB^f^
**Male models**							
	Cutoff	0.252	0.560	0.604	0.253	0.391	0.946
	AUC^g^ (95% CI)	0.827 (0.690-0.964)	0.787 (0.624-0.949)	0.794 (0.635-0.954)	0.900 (0.747-1.000)	0.896 (0.746-1.000)	0.767 (0.590-0.944)
	Sensitivity (95% CI)	0.900 (0.596-0.982)	0.300 (0.108-0.603)	0.500 (0.237-0.763)	0.900 (0.596-0.982)	0.700 (0.397-0.892)	0.600 (0.313-0.832)
	Specificity (95% CI)	0.673 (0.538-0.785)	0.981 (0.899-0.997)	0.904 (0.794-0.958)	0.750 (0.618-0.848)	0.942 (0.844-0.980)	0.827 (0.703-0.906)
	Accuracy (95% CI)	0.710 (0.587-0.808)	0.871 (0.766-0.933)	0.839 (0.728-0.910)	0.774 (0.656-0.860)	0.903 (0.805-0.955)	0.790 (0.674-0.873)
	PPV^h^ (95% CI)	0.346 (0.194-0.538)	0.750 (0.301-0.954)	0.500 (0.237-0.763)	0.409 (0.233-0.613)	0.700 (0.397-0.892)	0.400 (0.198-0.643)
	NPV^i^ (95% CI)	0.972 (0.858-0.995)	0.879 (0.771-0.94)	0.904 (0.794-0.958)	0.975 (0.871-0.996)	0.942 (0.844-0.980)	0.915 (0.801-0.966)
	*F*_1_-score	0.500	0.429	0.500	0.563	0.700	0.480
	Brier score	0.159	0.117	0.126	0.092	0.072	0.289
**Female models**							
	Cutoff	0.568	0.423	0.504	0.510	0.577	0.545
	AUC (95% CI)	0.762 (0.632-0.892)	0.692 (0.547-0.837)	0.670 (0.522-0.818)	0.644 (0.498-0.790)	0.716 (0.575-0.856)	0.628 (0.477-0.779)
	Sensitivity (95% CI)	0.679 (0.493-0.821)	0.857 (0.685-0.943)	0.643 (0.458-0.793)	0.429 (0.265-0.609)	0.643 (0.458-0.793)	0.643 (0.458-0.793)
	Specificity (95% CI)	0.815 (0.633-0.918)	0.519 (0.340-0.693)	0.704 (0.515-0.841)	0.778 (0.592-0.894)	0.741 (0.553-0.868)	0.556 (0.373-0.724)
	Accuracy (95% CI)	0.745 (0.617-0.842)	0.691 (0.560-0.797)	0.673 (0.541-0.782)	0.600 (0.468-0.719)	0.691 (0.560-0.797)	0.600 (0.468-0.719)
	PPV (95% CI)	0.792 (0.595-0.908)	0.649 (0.488-0.782)	0.692 (0.500-0.835)	0.667 (0.437-0.837)	0.720 (0.524-0.857)	0.600 (0.423-0.754)
	NPV (95% CI)	0.710 (0.534-0.839)	0.778 (0.548-0.910)	0.655 (0.473-0.801)	0.568 (0.409-0.713)	0.667 (0.488-0.808)	0.600 (0.407-0.766)
	*F*_1_-score	0.731	0.739	0.667	0.522	0.679	0.621
	Brier score	0.209	0.221	0.224	0.237	0.220	0.288

^a^LR: logistic regression.

^b^RF: random forest.

^c^XGBoost: extreme gradient boosting.

^d^KNN: k-nearest neighbor.

^e^SVM: support vector machine.

^f^NB: naïve Bayes.

^g^AUC: area under the curve.

^h^PPV: positive predictive value.

^i^NPV: negative predictive value.

### Model Stability Analysis

Figure S3 in Multimedia Appendix 1 presents the detailed results of the 10-fold cross-validation. The selected models, SVM for the male group and LR for the female group, achieved a 10-fold cross-validation AUC of 0.922 (SD 0.079) and 0.825 (SD 0.110) within the training sets, respectively. Table S7 in Multimedia Appendix 1 shows the cross-validation results as well as the discriminative performance for sarcoidosis versus each specific comparator disease.

### Model Explanation

The SHAP method explains the final model’s output by calculating the contribution of each variable to the prediction, aiding clinicians in understanding and trusting the diagnostic model. The global interpretation, depicted in the SHAP summary plot (Figure 3B), illustrates the collective impact and importance of all features. The mean SHAP value was used to assess each feature’s overall contribution to the model. In the male model, Lym# and TP were the most important features. In the female model, the most significant predictor was sACE, followed by the TG/HDLC (Figure S4 in Multimedia Appendix 1).

SHAP dependence plots help visualize how a single feature influences the model’s output across its range of values, showing the relationship between the feature value and its SHAP value (Figure 3A). A SHAP value greater than 0 corresponds to the positive class in the model’s prediction, which, in this study, indicates the pulmonary sarcoidosis class. Figure 3D is a beeswarm plot. Each point represents one sample, and the x axis indicates the SHAP value of the corresponding feature for that sample. A positive SHAP value means the feature increases the model’s output (eg, the predicted probability of sarcoidosis), while a negative SHAP value means it decreases the output. The color of each point represents the original value of that feature in the sample. By examining the distribution of points and the color gradient, one can infer the relationship between the feature value and its SHAP contribution. For example, if high feature values cluster in the positive SHAP region, higher values of that feature tend to push the prediction toward the positive class; conversely, if high values cluster in the negative SHAP region, higher values are associated with a negative prediction. Force plots (or waterfall plots) detail the prediction for an individual sample, demonstrating how each feature value contributes to pushing the model’s base value toward the final prediction output for that specific case (Figure 3C and E).

**Figure 3:**
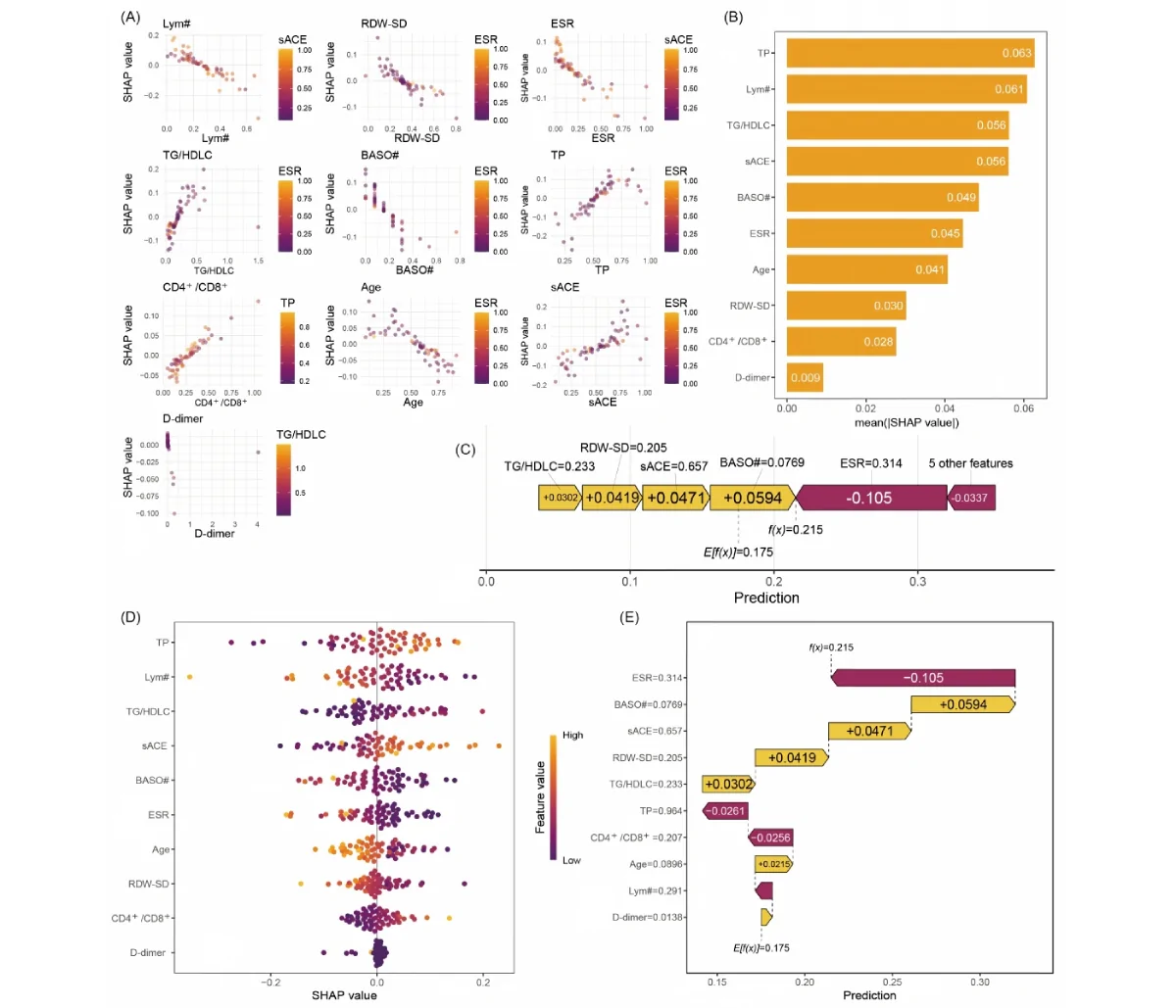
The feature importance and interpretation of the male model (based on the support vector machine algorithm). (A) SHAP dependence plot: association between each feature and its SHAP value. Point color represents the interaction effect between 2 features. (B) SHAP summary bar plot: ranking the importance of features. (C) SHAP force plot. (D) SHAP summary dot plot: relationship between feature values and SHAP values. The probability of pulmonary sarcoidosis classification increases with the SHAP value of a feature. (E) Waterfall plot: feature contributions to an individual prediction. The impact of a feature increases with the magnitude of its SHAP value. BASO#: basophil count; CD: cluster of differentiation; ESR: erythrocyte sedimentation rate; Lym#: lymphocyte count; RDW-SD: red cell distribution width standard deviation; sACE: serum angiotensin-converting enzyme; SHAP: Shapley Additive Explanations; TG/HDLC: triglyceride to high-density lipoprotein cholesterol ratio; TP: total protein.

### Web Accessibility

The final predictive model was implemented in a web-based application to enhance its utility in clinical practice, as illustrated in Figure 4 and Figure S5 in Multimedia Appendix 1. Upon entering the required feature values, the application automatically generates a diagnostic output. Additionally, an individualized force plot is displayed for each patient, indicating which features contributed to the sarcoidosis prediction: features on the right in red push the diagnosis toward “nonsarcoidosis,” while those on the left in yellow support a “pulmonary sarcoidosis” classification. The web applications for the male and female models are publicly accessible for research and demonstration purposes [30,31].

**Figure 4:**
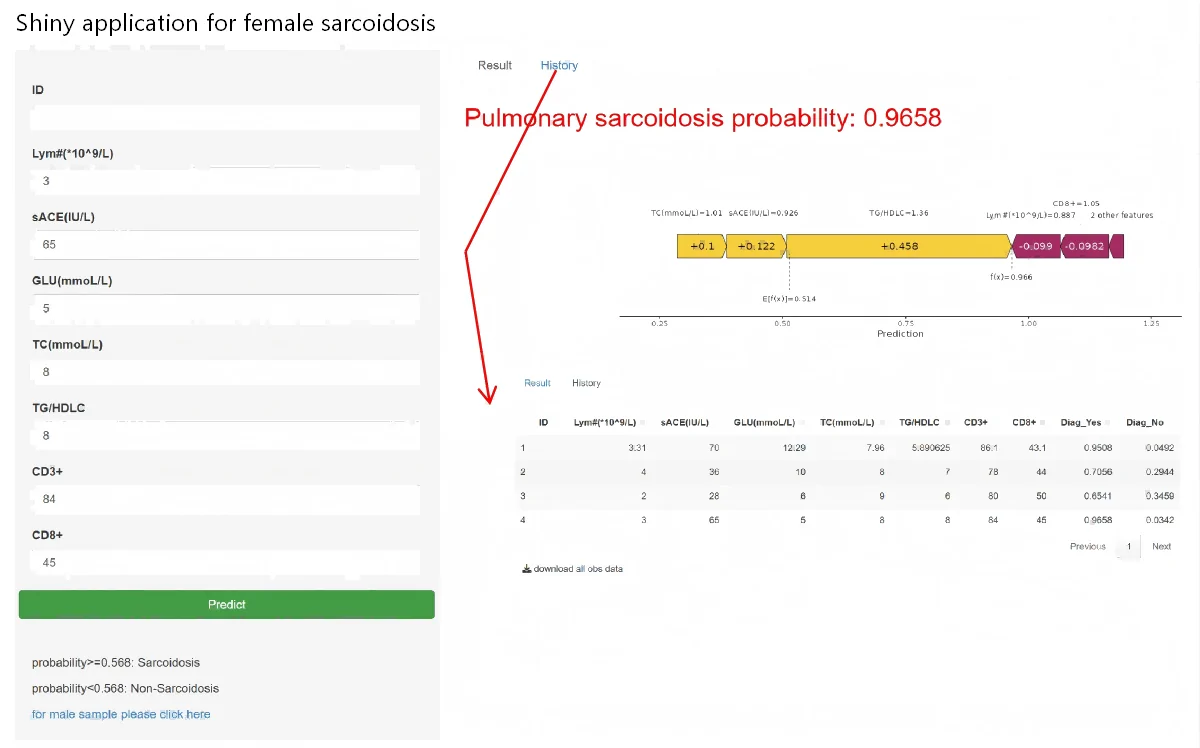
A convenient online clinical practice application featuring SHAP (female). CD: cluster of differentiation; GLU: glucose; Lym#: lymphocyte count; sACE: serum angiotensin-converting enzyme; SHAP: Shapley Additive Explanations; TC: total cholesterol; TG/HDLC: triglyceride to high-density lipoprotein cholesterol ratio.

## Discussion

### Principal Findings

This study developed diagnostic models to differentiate early-stage pulmonary sarcoidosis with normal sACE activities from cases of other atypical pulmonary diseases. To our knowledge, this is the first ML-based diagnostic model for pulmonary sarcoidosis. Our study indicated that, although sarcoidosis itself is a highly heterogeneous disease, sex-stratified ML models may provide useful discrimination between pulmonary sarcoidosis and other similar diseases, with relatively better performance observed in the male group.

The model for male patients incorporated the following indices: age, Lym#, RDW-SD, sACE, CD4^+^/CD8^+^, ESR, TG/HDLC, BASO#, TP, and D-dimer. The model for female patients used Lym#, sACE, TC, TG/HDLC, glucose, CD3^+^%, and CD8^+^%. We developed a web-based tool using the selected ML algorithms—the SVM model for male group (training set: AUC 0.989, 95% CI 0.975-1.000; validation set: AUC 0.896, 95% CI 0.746-1.000) and the LR model for female group (training set: AUC 0.848, 95% CI 0.781-0.915; validation set: AUC 0.762, 95% CI 0.632-0.892)—to facilitate the clinical application of this early pulmonary sarcoidosis differential diagnosis model. Additionally, we visualized the model using the SHAP method to enhance clinicians’ understanding.

Our model showed promising performance across internal cross-validation and analyses of individual diseases, although further validation in independent cohorts is needed to confirm its generalizability. This study fully considered sex differences in sarcoidosis and adopted a sex-stratified modeling strategy. The National Institutes of Health policy on Sex as a Biological Variable, issued in 2015 [32], calls for the inclusion of sex as a biological factor in biomedical research. Multiple studies have shown that there are significant immunological, clinical, and genetic differences between male and female patients with sarcoidosis [33-35]. A study on the pulmonary functional phenotype of sarcoidosis found that there are differences in pulmonary functional phenotypes between the sexes [36]. In addition, the association between certain human leukocyte antigen markers and sarcoidosis also shows sex differences [34], suggesting that immune pathways play different roles in the male and female groups. In this study, the selected model was SVM for the male group and LR for the female group, a difference that can be attributed to distinct intrinsic data characteristics between the 2 groups. The indices included in the male model may exhibit nonlinear relationships or threshold effects with pulmonary sarcoidosis. SVM can effectively capture complex nonlinear relationships among variables without requiring predefined functional assumptions. They are particularly suitable for small-to-moderate sample sizes and high-dimensional datasets and have demonstrated strong robustness and favorable generalization performance in various classification tasks. The female group had a smaller sample size and fewer features; LR is suitable for small-sample data and is less prone to overfitting, and its low-variance property makes it more robust. Moreover, the sets of included indices differ slightly between sexes (eg, D-dimer, RDW-SD, and BASO# in the male group; TC, glucose, and CD3^+^% in the female group), suggesting that sex-specific modeling helps remove confounding bias and improve predictive performance.

To address the class imbalance in the male data, we applied sample weighting during model development, which effectively increased the influence of the minority class (sarcoidosis). As a result, the model showed preliminary discriminative ability on the validation set (male SVM: AUC=0.896; sensitivity=0.700; specificity=0.942). The male model showed relatively better performance across several evaluation metrics than the female model, although the differences should be interpreted cautiously, given the limited sample size and wide CIs. Studies have suggested that estrogen exposure may contribute to the delayed onset of sarcoidosis in the female group by influencing the Th1/Th2 balance [37,38]. In this study, estrogen levels in the female patients fluctuate considerably with the menstrual cycle and age. This may result in greater heterogeneity or atypical patterns in immune-related biomarkers among female patients, leading to a more ambiguous and noisier relationship between features and outcomes, thereby reducing the predictive power of the model. Additionally, the male group in this study had a slightly larger sample size (n=224 cases) than the female group (n=196 cases), and the male model included more features (n=10) than the female model (n=7). These factors may also partly explain the more stable performance of the male model.

It should be noted that although the RF algorithm uses bootstrap sampling and ensemble learning (bagging) to mitigate overfitting, the AUC on the training set in this study still reached 1.000. This suggests that the current hyperparameter tuning range (eg, tree depth, minimum node size, and regularization parameters) may still be insufficient to adequately penalize model complexity, or alternatively, the combination of a small sample size and a high-dimensional feature space may have allowed the model to easily memorize noise in the training data. Specifically, while bootstrap sampling reduces variance, when there are many features or strong discriminative predictors in the data, individual trees can still achieve near-perfect splits on the training samples; after aggregating multiple trees, the predictions on the training set become highly consistent, resulting in an AUC of 1. The relatively limited sample size in this study further exacerbates this issue. Future research should adopt stricter regularization strategies (eg, limiting tree depth, reducing the number of features, and increasing pruning strength) alongside larger sample sizes to enhance model generalizability.

All indices incorporated in our model are routinely available in clinical practice, including complete blood count, inflammatory markers, T-cell subsets, biochemical tests, and lipid profiles, offering good operability and generalizability. Moreover, these indices collectively reflect multiple pathophysiological dimensions—immune activation, chronic inflammation, metabolic disturbance, and nutritional status—closely aligning with the complex mechanisms of pulmonary nodular granulomatous disease. Traditional approaches relying on a single index or manually exploring combinations of indices have often been inefficient and difficult to translate into clinical use. To address this, we used the Boruta algorithm combined with LASSO regression for feature selection, efficiently identifying the above representative and jointly effective indices.

Both models included sACE and T-cell subset indices, which are the most common biomarkers for sarcoidosis. In this research, despite sACE levels of all the cases being within the normal ranges, sACE ranked fourth in importance in the male model but first in the female model, possibly due to the higher prevalence of pneumoconiosis—which also elevates sACE—among the male group [39]. However, sACE activity has been found to be associated with population characteristics. In certain populations, sACE levels fall within the high end of the normal range, which is associated with the angiotensin-converting enzyme gene insertion/deletion polymorphism (ACE I/D) [40]. Research by Csongrádi et al [41] found that combining sACE activity testing with ACE I/D genotyping improved the specificity of sarcoidosis diagnosis to 100%, albeit with a relatively low sensitivity of 42.5%. Both male and female groups showed a reduction in Lym# in peripheral blood, which may be associated with more impaired cellular immune function, for example, dysregulation of T cell subsets, in sarcoidosis than in other differentiated diseases [42]. Elevated CD4^+^/CD8^+^ ratios in bronchoalveolar lavage fluid have been demonstrated in multiple studies to hold diagnostic value for sarcoidosis [43]. Nevertheless, the invasive risk for bronchoalveolar lavage fluid sampling limits its clinical applicability. Our study demonstrated that the peripheral blood CD4^+^/CD8^+^ ratio in male, and percentages of CD8^+^ and CD3^+^ in female, when combined with other readily available indicators, can yield a well-performing diagnostic model for sarcoidosis.

Patients with sarcoidosis exhibit dyslipidemia. An integrative observational and Mendelian randomization study on blood lipids and sarcoidosis confirmed that high-density lipoprotein cholesterol and TC are closely associated with the disease [26], which may be attributed to abnormal lipid metabolism in macrophages during sarcoidosis pathogenesis [44]. Consistently, in this study, 2 indices reflecting lipid metabolic status [45], TC and TG/HDLC ratio, were selected. Currently, the TG/HDLC ratio has been extensively studied in metabolic and cardiovascular diseases [46,47]; its relationship pattern with sarcoidosis warrants further investigation. Our model, leveraging easily obtainable samples and providing rapid results, offers a promising solution for the early differential diagnosis of pulmonary sarcoidosis. In addition, the web-based applications developed in this study are research prototypes intended solely for demonstration and peer review purposes. Translation into clinical practice would require implementation of the following security measures: (1) deploy the application within the hospital’s internal network and enforce user authentication or authorization via Lightweight Directory Access Protocol or Shiny Proxy; (2) use HTTPS to encrypt all data transmission; (3) do not store any patient identifiable information; input data are used only for the current prediction and are not persisted; (4) maintain audit logs (timestamp, user, and deidentified input values) for traceability; (5) establish model version control and an approval workflow for updates; and (6) include a clear disclaimer that the tool is for decision support only, and the final diagnosis must be made by a clinician.

### Limitations and Future Directions

This study has several limitations. First, the single-center nature of our study represents a key limitation, as it introduces potential selection bias and may restrict the model’s generalizability to other demographic and clinical environments. Although internal cross-validation was robust, this constraint necessitates future external validation. Multicenter collaborations are needed to test, refine, and ultimately ensure the broad clinical applicability of the model. Second, although we split an independent validation set and performed robustness analyses, the inherent limitation of sample size remains, which reduces the reliability of the DCA and calibration curves. Future studies with larger sample sizes and external validation are needed to further confirm the robustness and generalizability of the models. Third, class imbalance exists in the male data. Although we applied sample weighting during modeling and acceptable predictive capacity on the validation set, increasing the male sample size and using other methods to address class imbalance are still necessary in the future to enhance model performance and generalizability. Fourth, the cohort splitting strategy was performed using random sampling rather than stratified sampling. Although the distributions of disease categories were comparable between the training and validation cohorts in the present study, future multicenter studies with larger cohorts should consider stratified sampling based on disease categories and external validation to further assess the robustness, clinical applicability, and generalizability of the proposed model. Fifth, although both models demonstrated acceptable discriminative ability, the CIs for sensitivity remained relatively wide. This is partly due to the small sample size and partly because some features of sarcoidosis (eg, elevated sACE and decreased lymphocytes) may also be present in other diseases. Future studies should adopt larger sample sizes and explore novel blood biomarkers. Sixth, the data incorporated into our model consisted of peripheral blood biomarkers, which are subject to inherent variability. However, all blood samples were collected as fasting morning draws, and we excluded patients with a history of medication use (eg, corticosteroids) prior to admission to minimize confounding factors. Seventh, we included patients who had used cephalosporins within 2 weeks, because these drugs are commonly used as empirical antibiotics in clinical practice. To preserve sample size and reflect real-world clinical scenarios, we retained such cases; moreover, short-term use of cephalosporins has a minimal impact on blood parameters [48] (eg, the half-life of ceftriaxone is approximately 1.5-2 hours) [49]. Nevertheless, we acknowledge that incomplete control of antibiotic exposure remains a potential confounder. We suggest that future studies with stricter inclusion criteria or prospective designs may further address this issue. Eighth, although we performed robustness and generalizability analyses for sarcoidosis versus each single comparator disease, the sample sizes for some diseases were too small to allow meaningful statistical analysis. Therefore, the model’s generalizability to rare diseases such as lymphoma remains unclear. Future multicenter studies with larger sample sizes are needed to comprehensively validate the model’s generalizability across all comparator diseases. Ninth, while the webpage is flexible and user-friendly, it must undergo prospective validation and obtain ethics approval before being implemented in clinical practice.

### Conclusions

This study developed an interpretable model for the differential diagnosis of early pulmonary sarcoidosis versus other radiologically atypical pulmonary diseases with normal sACE levels using conventional peripheral blood markers. Given the wide CIs reflecting limited sample size, these findings are preliminary and require validation in larger independent cohorts. If successfully validated, the model holds promise to support clinical decision-making, facilitate the management of early-stage pulmonary sarcoidosis, and provide a scientific basis for clinicians when considering invasive diagnostic examinations.

## Appendix

Multimedia Appendix 1 Additional analyses and model evaluation results.

Multimedia Appendix 2 R code.

## Abbreviations

ACE I/D: angiotensin-converting enzyme gene insertion/deletion polymorphism
AUC: area under the curve
BASO#: basophil count
CD: cluster of differentiation
CT: computed tomography
DCA: decision curve analysis
ESR: erythrocyte sedimentation rate
KNN: k-nearest neighbor
LASSO: least absolute shrinkage and selection operator
LR: logistic regression
Lym#: lymphocyte count
ML: machine learning
MLNT: mediastinal lymph node tuberculosis
NB: naïve Bayes
RDW-SD: red cell distribution width standard deviation
RF: random forest
sACE: serum angiotensin-converting enzyme
SDS: sarcoidosis diagnostic score
SHAP: Shapley Additive Explanations
SVM: support vector machine
TC: total cholesterol
TG/HDLC: triglyceride to high-density lipoprotein cholesterol ratio
TP: total protein
XGBoost: extreme gradient boosting

## Data Availability

The datasets used and analyzed during this study are available from the corresponding author on reasonable request. The R scripts and computational codes used for data processing, model development, validation, and statistical analyses are provided as Multimedia Appendix 2.
