# Supplementary material for: Differential Diagnosis of Early-Stage Pulmonary Sarcoidosis With Normal Serum Angiotensin-Converting Enzyme Activity Using Peripheral Blood Indicators: Machine Learning Models Development and Validation
Source: JMIR Med Inform. 2026 Oct 26;14:e86639. doi: 10.2196/86639 (PMC13749020; doi:10.2196/86639)
Supplement: Supplementary file 1 [file medinform_v14i1e86639_app1.docx]

**Table S1.** Reference ranges of peripheral blood indicators

| Indices | Full name | Reference range | Unit |
| --- | --- | --- | --- |
| ALB | Albumin | 32-48 | g/L |
| APOA1 | Apoprotein A-1 | 1.04-2.02 | g/L |
| APOB | Apoprotein B | 0.66-1.33 | g/L |
| APOE | Apoprotein E | 27-45 | mg/L |
| APTT | Serum Activated Partial Thromboplatin Time | 25.1-36.5 | sec |
| AT-III | Serum Antithrombin III | 83-128 | % |
| BASO# | Basophils Count | 0.00-0.10 | ×10^9^/L |
| BASO% | Basophils Percentage | 0.0-1.0 | % |
| CA | Calcium | 2.18-2.60 | mmoL/L |
| C3 | Complement 3 | 0.9-1.8 | g/L |
| CRP | C-Reactive Protein | <10 | mg/L |
| D-dimer | D-dimer | 0-232 | ng/mL |
| EO# | Eosinophils Count | 0.05-0.30 | × 10^9^/L |
| EO% | Eosinophils Percentage | 1.0-5.0 | % |
| ESR | Erythrocyte Sedimentation Rate | 0-20 | mm/h |
| FIB | Serum Fibrinogen | 2.38-4.98 | g/L |
| FDP | Serum Fibrin(ogen) Degradation Products | <5 | ug/mL |
| GLU | Glucose | 4.1-5.9 | mmoL/L |
| HCT | Hematocrit | 0.35-0.45 | L/L |
| HDLC | High-Density Lipoprotein Cholesterol | >1.45 | mmoL/L |
| HGB | Haemoglobin | 130-175 | g/L |
| IgA | Immunoglobulin A | 0.7-4 | g/L |
| IgG | Immunoglobulin G | 7-16 | g/L |
| IgM | Immunoglobulin M | 0.4-2.3 | g/L |
| INR | Serum International Normalized Ratio | 0.80-1.10 | - |
| LDLC | Low-Density Lipoprotein Cholesterol | <3.37 | mmoL/L |
| LPA | Lipoprotein(a) | 0-300 | mg/L |
| Lym# | Lymphocytes Count | 0.80-4.00 | ×10^9^/L |
| Lym% | Lymphocytes Percentage | 20-40 | % |
| MON# | Monocytes Count | 0.10-0.80 | × 10^9^/L |
| MON% | Monocytes Percentage | 3-8 | % |
| MCV | Mean Corpuscular Volume | 82.0-100.0 | fL |
| MCH | Mean Corpuscular Haemoglobin | 27-34 | pg |
| MCHC | Mean Corpuscular Haemoglobin Concentration | 316-354 | g/L |
| MPV | Mean Platelet Volume | 9-13 | fL |
| NEU# | Neutrophils Count | 2.00-7.70 | ×10^9^/L |
| NEU% | Neutrophils Percentage | 50-70 | % |
| PCT | Platecrit | 0.114-0.282 | % |
| PDW | Platelet Distribution Width | 9-17 | fL |
| P-LCR | Platelet Large Cell Ratio | 13-43 | % |
| PLT | Platelets Count | 125-350 | × 10^9^/L |
| PT | Serum Prothrombin Time | 9.4-12.5 | sec |
| RBC | Red Blood Cell | 4.3-5.8 | ×10^12^/L |
| RDW-CV | Red cell Distribution Width - Coefficient of Variation | 11-16 | % |
| RDW-SD | Red cell Distribution Width - Standard Deviation | 37.0-54.0 | fL |
| sACE | serum Angiotensin-Converting Enzyme | 0-70 | IU/L |
| TC | Total Cholesterol | 2.80-5.85 | mmoL/L |
| TG | Triglyceride | <1.8 | mmoL/L |
| TP | Total Protein | 57-82 | g/L |
| TRF | Transferrin | 2.0-3.6 | g/L |
| TT | Serum Thrombin Time | 10.30-16.60 | sec |
| WBC | White Blood Cell | 3.5-9.5 | ×10^9^/L |

**Table S2.** Summary of missing data (male model)

|  | training set | | validation set | |
| --- | --- | --- | --- | --- |
|  | Missing Count | Missing Percent | Missing Count | Missing Percent |
| P-LCR | 12 | 7.41 | 5 | 8.06 |
| PDW | 9 | 5.56 | 4 | 6.45 |
| LPA/APOE | 9 | 5.56 | 4 | 6.45 |
| APOA1/APOB | 8 | 4.94 | 2 | 3.23 |
| APOE | 8 | 4.94 | 4 | 6.45 |
| LPA | 8 | 4.94 | 5 | 8.06 |
| IgG | 7 | 4.32 | 2 | 3.23 |
| IgA | 7 | 4.32 | 3 | 4.84 |
| IgM | 7 | 4.32 | 3 | 4.84 |
| C3 | 7 | 4.32 | 3 | 4.84 |
| TRF | 7 | 4.32 | 2 | 3.23 |
| APOA1 | 7 | 4.32 | 2 | 3.23 |
| APOB | 7 | 4.32 | 2 | 3.23 |
| ESR | 5 | 3.09 | 2 | 3.23 |
| D-Dimer | 5 | 3.09 | 1 | 1.61 |
| FDP | 4 | 2.47 | 3 | 4.84 |
| PT | 2 | 1.23 | 0 | 0 |
| INR | 2 | 1.23 | 0 | 0 |
| FIB | 2 | 1.23 | 0 | 0 |
| APTT | 2 | 1.23 | 0 | 0 |
| TT | 2 | 1.23 | 0 | 0 |
| AT3 | 2 | 1.23 | 1 | 1.61 |
| LDLC | 1 | 0.62 | 1 | 1.61 |
| LDLC/HDLC | 1 | 0.62 | 1 | 1.61 |

**Table S3.** Summary of missing data (female model)

|  | training set | | validation set | |
| --- | --- | --- | --- | --- |
|  | Missing Count | Missing Percent | Missing Count | Missing Percent |
| IgG | 14 | 9.93 | 4 | 7.27 |
| IgA | 14 | 9.93 | 4 | 7.27 |
| IgM | 14 | 9.93 | 4 | 7.27 |
| C3 | 14 | 9.93 | 4 | 7.27 |
| TRF | 14 | 9.93 | 4 | 7.27 |
| P-LCR | 8 | 5.67 | 3 | 5.45 |
| PDW | 6 | 4.26 | 1 | 1.82 |
| LPA/APOE | 6 | 4.26 | 6 | 10.91 |
| ESR | 6 | 4.26 | 3 | 5.45 |
| MPV | 5 | 3.55 | 2 | 3.64 |
| APOB | 5 | 3.55 | 4 | 7.27 |
| APOA1/APOB | 5 | 3.55 | 4 | 7.27 |
| APOE | 5 | 3.55 | 5 | 9.09 |
| LPA | 5 | 3.55 | 5 | 9.09 |
| PCT | 4 | 2.84 | 1 | 1.82 |
| APOA1 | 4 | 2.84 | 4 | 7.27 |
| LDLC | 2 | 1.42 | 0 | 0 |
| LDLC/HDLC | 2 | 1.42 | 0 | 0 |
| D-dimer | 2 | 1.42 | 1 | 1.82 |
| RDW-SD | 1 | 0.71 | 0 | 0 |
| RDW-CV | 1 | 0.71 | 0 | 0 |
| TP | 1 | 0.71 | 0 | 0 |
| ALB | 1 | 0.71 | 0 | 0 |
| CA | 1 | 0.71 | 1 | 1.82 |
| TG/HDLC | 1 | 0.71 | 0 | 0 |
| PT | 1 | 0.71 | 0 | 0 |
| INR | 1 | 0.71 | 0 | 0 |
| FIB | 1 | 0.71 | 0 | 0 |
| APTT | 1 | 0.71 | 0 | 0 |
| TT | 1 | 0.71 | 0 | 0 |
| AT3 | 1 | 0.71 | 0 | 0 |
| FDP | 1 | 0.71 | 1 | 1.82 |

**Table S4.** Results of univariate analysis for all variables in the male dataset

|  | training set | |  | validation set | |  |
| --- | --- | --- | --- | --- | --- | --- |
|  | Non-SAR (N=130) | SAR (N=32) | P-value | Non-SAR (N=52) | SAR (N=10) | P-value |
| Disease Category, n (%) | |  | <.001 |  |  | <.001 |
| Mediastinal lymph node tuberculosis | 25 (19.2) | 0 (0.0) |  | 14 (26.9) | 0 (0.0) |  |
| Sarcoidosis | 0 (0.0) | 32 (100) |  | 0 (0.0) | 10 (100) |  |
| Lymphoma | 5 (3.8) | 0 (0.0) |  | 4 (7.7) | 0 (0.0) |  |
| lungcancer | 80 (61.5) | 0 (0.0) |  | 26 (50.0) | 0 (0.0) |  |
| Pneumoconiosis | 20 (15.4) | 0 (0.0) |  | 8 (15.4) | 0 (0.0) |  |
| Age years, median (IQR) | 60.0 (45.2, 66.0) | 44.0 (37.8, 49.2) | <.001 | 57.0 (47.5, 66.0) | 41.0 (39.2, 53.2) | .02 |
| HGB, mean (SD) | 152 (21.1) | 159 (18.3) | .07 | 153 (139, 166) | 163 (156, 176) | .05 |
| RBC, mean (SD) | 5.11 (0.64) | 5.39 (0.64) | .03 | 5.22 (4.76, 5.52) | 5.62 (5.22, 5.88) | .04 |
| WBC, median (IQR) | 6.82 (5.39, 8.28) | 5.46 (4.59, 6.64) | .005 | 6.04 (5.41, 7.48) | 5.30 (4.27, 5.90) | .04 |
| NEU%, mean (SD) | 64.9 (9.69) | 65.9 (9.47) | .60 | 64.5 (10.5) | 64.1 (9.16) | .92 |
| Lym%^a^ | 24.9 (18.2, 30.2) | 23.0 (18.0, 26.3) | .42 | 25.5 (9.79) | 24.9 (6.92) | .83 |
| MON%, median (IQR) | 7.00 (5.50, 9.07) | 7.25 (6.00, 8.72) | .55 | 7.20 (6.20, 8.15) | 7.85 (5.77, 8.85) | .55 |
| EO%, median (IQR) | 2.00 (1.10, 3.40) | 2.20 (1.17, 3.25) | .61 | 1.95 (0.80, 3.52) | 2.10 (0.98, 3.50) | .87 |
| BASO%, median (IQR) | 0.30 (0.10, 0.50) | 0.20 (0.10, 0.30) | .008 | 0.30 (0.10, 0.40) | 0.20 (0.20, 0.30) | .82 |
| NEU#, median (IQR) | 4.39 (3.48, 5.74) | 3.64 (2.94, 4.73) | .03 | 4.20 (3.58, 5.11) | 3.38 (3.00, 3.84) | .04 |
| Lym#, median (IQR) | 1.62 (1.20, 2.06) | 1.39 (1.08, 1.73) | .01 | 1.56 (1.20, 2.11) | 1.24 (1.03, 1.79) | .20 |
| NEU/Lym, median (IQR) | 2.65 (1.94, 3.82) | 2.71 (2.15, 3.91) | .54 | 2.62 (1.92, 3.57) | 2.78 (2.11, 3.59) | .82 |
| MON#^a^ | 0.46 (0.38, 0.63) | 0.45 (0.36, 0.50) | .27 | 0.48 (0.15) | 0.45 (0.22) | .74 |
| MON/Lym | 0.30 (0.19, 0.43) | 0.32 (0.25, 0.42) | .28 | 0.29 (0.21, 0.37) | 0.30 (0.24, 0.43) | .75 |
| EO#, median (IQR) | 0.14 (0.07, 0.24) | 0.15 (0.07, 0.18) | .60 | 0.12 (0.06, 0.22) | 0.11 (0.06, 0.21) | .75 |
| BASO#, median (IQR) | 0.02 (0.01, 0.03) | 0.01 (0.01, 0.01) | <.001 | 0.02 (0.01, 0.03) | 0.01 (0.01, 0.02) | .39 |
| HCT^a^ | 0.40 (0.05) | 0.41 (0.05) | .14 | 0.40 (0.36, 0.42) | 0.44 (0.41, 0.45) | .005 |
| MCV^a^ | 95.6 (89.2, 103) | 90.9 (87.0, 95.2) | .005 | 94.6 (9.91) | 93.9 (8.60) | .81 |
| MCH^a^ | 31.3 (29.3, 33.8) | 30.5 (30.0, 31.7) | .22 | 31.0 (3.18) | 30.7 (2.61) | .80 |
| MCHC, mean (SD) | 342 (13.0) | 346 (10.9) | .12 | 343 (12.6) | 340 (9.17) | .41 |
| PLT#^a^ | 274 (228, 344) | 237 (204, 271) | .01 | 257 (69.4) | 224 (77.0) | .22 |
| PLT/Lym, median (IQR) | 148 (103, 208) | 153 (130, 186) | .50 | 147 (104, 176) | 147 (123, 162) | .67 |
| PLT/NEU, median (IQR) | 55.8 (43.2, 71.1) | 53.0 (42.2, 70.2) | .69 | 53.6 (44.5, 71.9) | 56.9 (45.3, 68.9) | .89 |
| RDW-SD, median (IQR) | 43.0 (40.6, 45.0) | 40.5 (38.5, 41.7) | <.001 | 42.3 (40.6, 43.8) | 41.7 (40.3, 43.9) | .66 |
| RDW-CV, median (IQR) | 12.8 (12.3, 13.5) | 12.6 (12.1, 13.0) | .06 | 12.9 (12.5, 13.3) | 12.9 (12.4, 13.3) | .85 |
| PCT^a^ | 0.24 (0.21, 0.31) | 0.22 (0.19, 0.26) | .048 | 0.23 (0.05) | 0.22 (0.06) | .63 |
| PDW, median (IQR) | 11.8 (11.0, 13.1) | 12.7 (11.4, 13.9) | .07 | 12.1 (11.1, 13.9) | 13.8 (11.3, 16.0) | .16 |
| P-LCR^a^ | 27.5 (23.2, 32.6) | 29.2 (25.6, 35.6) | .21 | 29.0 (8.72) | 36.1 (11.9) | .10 |
| MPV^a^ | 10.2 (9.70, 11.0) | 10.6 (10.1, 11.1) | .11 | 10.4 (1.22) | 11.3 (1.55) | .10 |
| IgG, median (IQR) | 12.6 (10.9, 15.2) | 12.0 (11.1, 13.1) | .09 | 12.0 (10.7, 13.4) | 13.5 (11.6, 15.1) | .30 |
| IgA, median (IQR) | 2.43 (1.96, 3.23) | 2.56 (1.97, 3.28) | .60 | 2.45 (2.03, 2.97) | 2.36 (1.81, 2.58) | .35 |
| IgM, median (IQR) | 0.91 (0.74, 1.48) | 0.90 (0.67, 1.05) | .04 | 1.02 (0.79, 1.40) | 0.83 (0.70, 0.99) | .25 |
| C3^a^ | 1.23 (1.07, 1.43) | 1.23 (1.12, 1.35) | .95 | 1.15 (0.22) | 1.15 (0.13) | .96 |
| TRF, mean (SD) | 2.15 (0.48) | 2.28 (0.45) | .18 | 2.03 (0.46) | 2.40 (0.33) | .008 |
| TP, mean (SD) | 63.9 (8.78) | 68.9 (6.90) | .001 | 63.2 (8.88) | 71.7 (9.45) | .02 |
| ALB, mean (SD) | 37.9 (5.00) | 41.5 (3.65) | <.001 | 37.5 (4.91) | 42.7 (4.22) | .004 |
| GLU, median (IQR) | 5.00 (4.60, 5.49) | 5.18 (5.00, 5.85) | .09 | 4.96 (4.64, 5.82) | 5.25 (4.95, 5.39) | .36 |
| CA, mean (SD) | 2.28 (0.14) | 2.29 (0.13) | .79 | 2.25 (0.15) | 2.42 (0.15) | .005 |
| sACE, mean (SD) | 34.0 (14.7) | 43.2 (16.6) | .006 | 35.7 (16.0) | 44.6 (9.61) | .03 |
| TC^a^ | 4.79 (3.91, 5.30) | 5.18 (4.88, 5.62) | .001 | 4.80 (0.94) | 5.12 (0.93) | .33 |
| TG, median (IQR) | 0.80 (0.62, 1.12) | 0.97 (0.74, 1.18) | .07 | 1.04 (0.67, 1.46) | 0.87 (0.77, 1.19) | .65 |
| HDLC, mean (SD) | 1.26 (0.39) | 1.39 (0.28) | .03 | 1.22 (0.36) | 1.43 (0.29) | .06 |
| TG/HDLC, median (IQR) | 0.95 (0.65, 1.45) | 1.39 (1.02, 2.06) | <.001 | 1.16 (0.67, 1.81) | 1.24 (0.71, 1.43) | .66 |
| LDLC^a^ | 2.52 (2.03, 3.08) | 2.68 (2.02, 3.35) | .21 | 2.80 (0.91) | 2.99 (0.84) | .52 |
| LDLC/HDLC, median (IQR) | 2.42 (1.81, 3.25) | 2.88 (2.10, 3.51) | .07 | 2.59 (2.14, 3.44) | 2.74 (2.14, 3.23) | .80 |
| APOA1^a^ | 1.25 (1.05, 1.49) | 1.24 (1.01, 1.37) | .31 | 1.21 (0.37) | 1.32 (0.27) | .29 |
| APOB, median (IQR) | 0.79 (0.63, 0.86) | 0.79 (0.66, 0.91) | .57 | 0.78 (0.66, 1.00) | 0.79 (0.67, 0.92) | .81 |
| APOA1/APOB, median (IQR) | 1.49 (1.29, 2.03) | 1.48 (1.26, 1.57) | .16 | 1.45 (1.07, 1.74) | 1.41 (1.27, 1.66) | .67 |
| APOE, median (IQR) | 32.7 (26.1, 39.5) | 35.6 (32.5, 41.7) | .07 | 34.6 (29.0, 44.0) | 39.1 (33.1, 47.1) | .44 |
| LPA, median (IQR) | 127 (60.5, 244) | 89.2 (54.1, 173) | .24 | 120 (75.9, 238) | 142 (86.5, 246) | .72 |
| LPA/APOE, median (IQR) | 3.51 (1.84, 7.30) | 2.36 (1.63, 3.92) | .09 | 3.54 (1.80, 6.90) | 3.62 (2.07, 6.28) | .92 |
| ESR, median (IQR) | 33.3 (17.5, 70.7) | 19.3 (14.3, 27.3) | .001 | 28.7 (13.8, 53.7) | 10.7 (7.33, 30.3) | .05 |
| CD3^+^, mean (SD) | 66.3 (11.9) | 64.9 (12.9) | .59 | 64.0 (10.3) | 64.5 (9.01) | .89 |
| CD3^+^CD16^+^CD56^+^, median (IQR) | 12.4 (6.60, 21.0) | 12.2 (7.70, 20.1) | .78 | 12.2 (7.27, 16.8) | 14.9 (7.32, 19.2) | .52 |
| CD3^++a^ | 64.0 (54.4, 71.1) | 63.4 (55.4, 73.7) | .84 | 63.7 (12.3) | 64.3 (7.76) | .84 |
| CD4^+^, mean (SD) | 34.3 (9.88) | 35.3 (11.5) | .64 | 35.0 (11.2) | 34.2 (6.01) | .76 |
| CD8^+^, median (IQR) | 19.2 (14.8, 26.2) | 14.9 (10.3, 23.5) | .05 | 17.9 (15.1, 23.5) | 24.6 (16.8, 28.9) | .18 |
| CD4^+^/CD8^+^, median (IQR) | 1.69 (1.25, 2.59) | 2.34 (1.45, 3.68) | .02 | 1.85 (1.15, 2.56) | 1.54 (1.19, 2.12) | .50 |
| PT^a^ | 11.4 (10.8, 11.9) | 11.4 (10.8, 11.9) | .84 | 11.1 (0.98) | 11.3 (0.74) | .55 |
| INR^a^ | 1.06 (1.00, 1.12) | 1.08 (1.03, 1.13) | .29 | 1.04 (0.11) | 1.08 (0.07) | .14 |
| FIB^a^ | 3.53 (2.96, 4.54) | 3.09 (2.76, 3.51) | .006 | 3.66 (1.00) | 3.05 (0.43) | .004 |
| APTT^a^ | 31.4 (29.2, 33.3) | 31.9 (30.1, 32.5) | .91 | 30.9 (3.13) | 31.6 (2.29) | .44 |
| TT, median (IQR) | 14.0 (12.8, 14.9) | 14.6 (13.2, 15.6) | .13 | 13.9 (12.9, 14.9) | 14.5 (13.9, 15.0) | .17 |
| AT3^a^ | 105 (97.0, 112) | 111 (104, 116) | .006 | 104 (15.1) | 106 (13.3) | .55 |
| D-dimer, median (IQR) | 144 (79.8, 351) | 123 (78.0, 161) | .03 | 161 (92.8, 341) | 104 (75.5, 161) | .12 |
| FDP, median (IQR) | 1.20 (0.50, 2.36) | 0.70 (0.50, 1.04) | .008 | 1.01 (0.44, 2.08) | 0.25 (0.10, 0.54) | .005 |

^a^Continuous variables are presented as mean (SD) or median (IQR), as appropriate.

%: Percentage of detected content, #: Count of detected content,

**Table S5.** Results of univariate analysis for all variables in the female dataset

|  | Training set | | | Validation set | | |
| --- | --- | --- | --- | --- | --- | --- |
|  | Nonsarcoidosis (N=69) | Sarcoidosis (N=72) | P-value | Nonsarcoidosis (N=27) | Sarcoidosis (N=28) | P-value |
| **Disease Category,n (%)** | |  | <.001 |  |  | <.001 |
| Mediastinal lymph node tuberculosis | 25 (36.2) | 0 (0.0) |  | 14 (51.9) | 0 (0.0) |  |
| Sarcoidosis | 0 (0.0) | 72 (100) |  | 0 (0.00) | 28 (100) |  |
| Lymphoma | 4 (5.8) | 0 (0.0) |  | 0 (0.0) | 0 (0.0) |  |
| Lung cancer | 38 (55.1) | 0 (0.0) |  | 11 (40.7) | 0 (0.0) |  |
| Pneumoconiosis | 2 (2.9) | 0 (0.0) |  | 2 (7.41) | 0 (0.0) |  |
| Age, years, median (IQR) | 55.0 (45.0, 64.0) | 54.0 (48.0, 60.0) | .51 | 56.0 (45.5, 64.0) | 53.0 (48.8, 60.2) | .83 |
| HGB^a^ | 143 (132, 154) | 148 (137, 154) | .26 | 143 (13.4) | 151 (14.2) | .04 |
| RBC, mean (SD) | 5.06 (0.41) | 5.13 (0.36) | .33 | 5.00 (0.41) | 5.20 (0.40) | .08 |
| WBC^a^ | 5.46 (4.29, 6.53) | 5.02 (3.90, 6.22) | .38 | 5.22 (1.86) | 5.26 (1.53) | .94 |
| NEU%, mean (SD) | 62.2 (9.42) | 64.4 (10.1) | .17 | 58.6 (10.3) | 64.3 (9.91) | .04 |
| Lym%, mean (SD) | 28.2 (8.85) | 26.2 (8.12) | .18 | 31.0 (9.59) | 25.8 (7.66) | .03 |
| MON%, median (IQR) | 7.00 (5.60, 8.80) | 6.40 (4.90, 8.45) | .17 | 6.60 (5.55, 8.50) | 6.55 (5.70, 7.75) | .51 |
| EO%, median (IQR) | 1.60 (1.10, 2.40) | 2.50 (1.20, 3.42) | .11 | 2.30 (0.85, 3.85) | 2.55 (1.75, 3.83) | .50 |
| BASO%, median (IQR) | 0.20 (0.20, 0.40) | 0.20 (0.20, 0.50) | .76 | 0.30 (0.20, 0.55) | 0.20 (0.08, 0.40) | .04 |
| NEU#, median (IQR) | 3.46 (2.51, 4.64) | 3.46 (2.52, 4.52) | .80 | 3.10 (2.49, 4.20) | 3.33 (2.81, 4.56) | .33 |
| Lym#^a^ | 1.52 (1.20, 1.86) | 1.39 (1.12, 1.72) | .07 | 1.66 (0.60) | 1.42 (0.44) | .10 |
| NEU/Lym, median (IQR) | 2.16 (1.71, 3.00) | 2.53 (1.92, 3.36) | .13 | 1.73 (1.52, 2.50) | 2.36 (1.79, 2.99) | .04 |
| MON#, median (IQR) | 0.39 (0.31, 0.51) | 0.34 (0.28, 0.43) | .04 | 0.34 (0.26, 0.50) | 0.36 (0.30, 0.42) | .93 |
| MON/Lym, median (IQR) | 0.25 (0.17, 0.37) | 0.24 (0.19, 0.33) | .93 | 0.24 (0.17, 0.34) | 0.26 (0.22, 0.31) | .31 |
| EO#, median (IQR) | 0.09 (0.06, 0.14) | 0.11 (0.06, 0.17) | .45 | 0.12 (0.06, 0.20) | 0.14 (0.11, 0.19) | .57 |
| BASO#, median (IQR) | 0.01 (0.01, 0.02) | 0.01 (0.01, 0.03) | .67 | 0.02 (0.01, 0.03) | 0.01 (0.01, 0.02) | .05 |
| HCT^a^ | 0.38 (0.34, 0.41) | 0.39 (0.36, 0.40) | .33 | 0.37 (0.03) | 0.39 (0.03) | .09 |
| MCV^a^ | 93.0 (86.0, 97.8) | 91.0 (87.0, 95.7) | .67 | 92.9 (7.46) | 92.7 (7.60) | .92 |
| MCH, median (IQR) | 29.6 (27.5, 31.2) | 29.4 (28.6, 31.1) | .80 | 30.0 (28.3, 31.6) | 30.8 (29.5, 32.1) | .42 |
| MCHC^a^ | 338 (332, 343) | 341 (332, 346) | .14 | 339 (8.03) | 343 (12.0) | .14 |
| PLT#^a^ | 270 (82.8) | 270 (89.7) | .99 | 264 (224, 298) | 242 (227, 273) | .28 |
| PLT/Lym, median (IQR) | 155 (118, 199) | 176 (128, 210) | .14 | 142 (103, 184) | 149 (127, 199) | .52 |
| PLT/NEU, median (IQR) | 69.1 (49.4, 93.9) | 70.9 (49.2, 86.5) | .93 | 69.6 (55.5, 88.5) | 63.3 (50.2, 76.3) | .14 |
| RDW-SD, median (IQR) | 41.8 (40.1, 43.8) | 41.5 (40.0, 43.3) | .38 | 41.8 (39.8, 43.3) | 41.2 (39.2, 42.9) | .50 |
| RDW-CV, median (IQR) | 13.0 (12.4, 13.4) | 12.9 (12.5, 13.3) | >.99 | 12.6 (12.4, 13.6) | 12.8 (12.3, 13.4) | .89 |
| PCT, median (IQR) | 0.24 (0.21, 0.29) | 0.24 (0.20, 0.30) | .90 | 0.25 (0.22, 0.28) | 0.23 (0.21, 0.24) | .14 |
| PDW, median (IQR) | 12.4 (10.9, 13.7) | 12.4 (11.5, 13.6) | .69 | 12.3 (11.3, 12.9) | 11.9 (11.5, 12.9) | .81 |
| P-LCR, median (IQR) | 29.5 (24.1, 36.2) | 29.5 (23.8, 35.0) | .93 | 30.9 (26.3, 33.5) | 29.5 (26.5, 31.5) | .58 |
| MPV^a^ | 10.7 (1.27) | 10.6 (1.01) | .88 | 10.6 (10.2, 11.0) | 10.4 (10.2, 10.8) | .32 |
| IgG, median (IQR) | 13.6 (12.2, 16.2) | 13.6 (11.7, 15.1) | .16 | 13.6 (11.3, 14.9) | 13.8 (12.0, 16.5) | .63 |
| IgA, median (IQR) | 2.60 (2.20, 3.89) | 2.58 (1.99, 3.12) | .05 | 2.73 (2.18, 3.82) | 2.55 (2.20, 2.82) | .29 |
| IgM, median (IQR) | 1.07 (0.78, 1.67) | 1.07 (0.88, 1.29) | .65 | 1.07 (0.93, 1.37) | 1.00 (0.83, 1.94) | .97 |
| C3, mean (SD) | 1.22 (0.21) | 1.17 (0.18) | .15 | 1.20 (0.19) | 1.20 (0.20) | .86 |
| TRF^a^ | 2.23 (1.92, 2.39) | 2.23 (2.10, 2.54) | .15 | 2.36 (0.38) | 2.45 (0.46) | .40 |
| TP^a^ | 67.0 (60.0, 72.0) | 69.2 (64.0, 74.2) | .05 | 67.7 (8.26) | 68.0 (6.46) | .90 |
| ALB, median (IQR) | 39.1 (36.0, 42.0) | 40.0 (38.0, 42.0) | .08 | 39.0 (38.0, 41.9) | 40.5 (37.8, 42.0) | .87 |
| GLU, median (IQR) | 5.00 (4.70, 5.40) | 5.43 (4.91, 6.26) | .002 | 5.10 (4.77, 5.35) | 5.35 (4.78, 5.63) | .44 |
| CA^a^ | 2.30 (2.20, 2.34) | 2.31 (2.20, 2.36) | .35 | 2.29 (0.11) | 2.31 (0.11) | .48 |
| sACE, mean (SD) | 35.2 (12.3) | 47.9 (14.1) | <.001 | 40.1 (12.9) | 49.8 (13.7) | .01 |
| TC^a^ | 4.99 (4.17, 5.64) | 5.31 (4.81, 5.65) | .02 | 5.01 (0.70) | 5.37 (0.91) | .11 |
| TG, median (IQR) | 0.95 (0.59, 1.28) | 1.09 (0.71, 1.50) | .18 | 0.85 (0.57, 1.21) | 0.92 (0.66, 1.39) | .24 |
| HDLC^a^ | 1.37 (1.04, 1.64) | 1.39 (1.12, 1.60) | .74 | 1.44 (0.33) | 1.45 (0.37) | .91 |
| TG/HDLC, median (IQR) | 0.87 (0.66, 1.68) | 1.37 (0.91, 2.13) | .008 | 0.89 (0.51, 1.42) | 1.18 (0.66, 1.84) | .26 |
| LDLC^a^ | 2.75 (2.17, 3.14) | 2.76 (2.31, 3.37) | .18 | 2.73 (0.69) | 2.98 (1.01) | .28 |
| LDLC/HDLC, mean (SD) | 2.46 (0.95) | 2.68 (0.87) | .14 | 2.42 (0.88) | 2.53 (0.67) | .60 |
| APOA1^a^ | 1.22 (1.03, 1.51) | 1.23 (1.06, 1.52) | .98 | 1.36 (0.47) | 1.34 (0.33) | .84 |
| APOB, median (IQR) | 0.75 (0.62, 1.03) | 0.86 (0.76, 1.04) | .06 | 0.72 (0.66, 0.93) | 0.88 (0.82, 1.13) | .06 |
| APOA1/APOB, median (IQR) | 1.58 (1.31, 1.99) | 1.50 (1.28, 1.79) | .21 | 1.84 (1.31, 2.50) | 1.48 (1.32, 1.66) | .21 |
| APOE^a^ | 40.3 (14.9) | 44.3 (10.9) | .08 | 38.8 (31.7, 43.4) | 42.0 (37.3, 49.5) | .14 |
| LPA, median (IQR) | 142 (78.0, 264) | 138 (83.4, 236) | .77 | 143 (50.5, 246) | 138 (62.6, 176) | .87 |
| LPA/APOE, median (IQR) | 3.44 (1.98, 6.25) | 3.30 (1.96, 5.97) | .60 | 3.30 (1.57, 6.86) | 3.30 (1.23, 5.25) | .74 |
| ESR, median (IQR) | 28.0 (14.0, 57.0) | 24.5 (15.8, 32.2) | .22 | 25.0 (11.5, 48.5) | 17.5 (8.75, 32.5) | .69 |
| CD3^+a^ | 72.4 (63.0, 76.4) | 63.4 (57.6, 70.8) | <.001 | 67.6 (12.4) | 67.6 (9.54) | .99 |
| CD3^+^CD16^+^CD56^+^, median (IQR) | 8.40 (5.90, 13.5) | 9.45 (6.30, 15.7) | .17 | 7.60 (4.30, 16.9) | 9.15 (6.30, 14.3) | .38 |
| CD3^++a^ | 68.1 (60.7, 74.5) | 62.3 (58.0, 71.2) | .01 | 65.4 (11.3) | 63.9 (12.8) | .65 |
| CD4^+^, mean (SD) | 40.2 (8.40) | 39.4 (10.9) | .66 | 39.3 (9.57) | 38.2 (14.3) | .75 |
| CD8^+^, median (IQR) | 20.7 (15.0, 25.6) | 15.7 (12.1, 21.4) | <.001 | 19.1 (16.0, 24.5) | 16.0 (12.5, 22.4) | .11 |
| CD4^+^/CD8^+^, median (IQR) | 2.11 (1.47, 2.64) | 2.62 (1.79, 3.36) | .00 | 1.78 (1.40, 2.81) | 2.20 (1.50, 3.13) | .41 |
| PT^a^ | 11.3 (10.9, 11.8) | 11.2 (10.6, 11.6) | .11 | 11.3 (0.94) | 11.1 (0.88) | .37 |
| INR^a^ | 1.07 (1.00, 1.11) | 1.06 (0.99, 1.10) | .18 | 1.06 (0.10) | 1.04 (0.10) | .39 |
| FIB, median (IQR) | 3.28 (2.90, 3.93) | 3.03 (2.69, 3.34) | .01 | 3.13 (2.82, 3.55) | 2.85 (2.63, 3.08) | .12 |
| APTT^a^ | 31.1 (3.07) | 30.8 (2.98) | .58 | 30.9 (2.34) | 30.5 (3.17) | .60 |
| TT^a^ | 14.2 (13.3, 15.3) | 14.8 (13.9, 15.6) | .03 | 14.6 (1.11) | 14.7 (1.59) | .76 |
| AT3^a^ | 102 (94.0, 113) | 102 (93.2, 112) | .85 | 107 (13.0) | 101 (10.0) | .08 |
| D-dimer, median (IQR) | 207 (103, 388) | 146 (81.5, 198) | .006 | 162 (93.0, 248) | 136 (87.5, 178) | .23 |
| FDP, median (IQR) | 1.26 (0.57, 2.75) | 0.90 (0.56, 1.45) | .04 | 1.06 (0.56, 1.66) | 0.98 (0.52, 1.31) | .42 |

^a^Continuous variables are presented as mean (SD) or median (IQR), as appropriate.

%: Percentage of detected content, #: Count of detected content,

**Table S6.** Diagnostic performance metrics of different machine learning algorithms of the training set

|  | LR | RF | XGBoost | KNN | SVM | NB |
| --- | --- | --- | --- | --- | --- | --- |
| male models |  |  |  |  |  |  |
| AUC (95%CI) | 0.953 (0.922–0.984) | 1.000 (0.999–1.000) | 0.997 (0.991–1.000) | 0.939 (0.899–0.979) | 0.989 (0.975–1.000) | 0.937 (0.892–0.981) |
| Sensitivity (95%CI) | 1.000 (1.000–1.000) | 1.000 (1.000–1.000) | 1.000 (1.000–1.000) | 0.969 (0.908–1.000) | 0.969 (0.908–1.000) | 0.938 (0.854–1.000) |
| Specificity (95%CI) | 0.754 (0.680–0.828) | 0.992 (0.977–1.000) | 0.985 (0.963–1.000) | 0.800 (0.731–0.869) | 0.946 (0.907–0.985) | 0.885 (0.830–0.940) |
| Accuracy (95%CI) | 0.802 (0.801–0.804) | 0.994 (0.994–0.994) | 0.988 (0.988–0.988) | 0.833 (0.832–0.835) | 0.951 (0.950–0.951) | 0.895 (0.894–0.896) |
| PPV (95%CI) | 0.500 (0.378–0.623) | 0.970 (0.911,1.028) | 0.941 (0.862–1.020) | 0.544 (0.415–0.673) | 0.816 (0.693–0.939) | 0.667 (0.529–0.804) |
| NPV (95%CI) | 1.000 (1.000–1.000) | 1.000 (1.000–1.000) | 1.000 (1.000–1.000) | 0.990 (0.972–1.009) | 0.992 (0.976–1.008) | 0.983 (0.959–1.006) |
| *F1*-score | 0.667 | 0.985 | 0.970 | 0.670 | 0.886 | 0.779 |
| Brier score | 0.094 | 0.031 | 0.043 | 0.095 | 0.034 | 0.190 |
| female models |  |  |  |  |  |  |
| AUC (95%CI) | 0.848 (0.781–0.915) | 0.998 (0.994–1.000) | 0.887 (0.832–0.941) | 0.789 (0.715–0.864) | 0.876 (0.815–0.937) | 0.853 (0.789–0.917) |
| Sensitivity (95%CI) | 0.722 (0.619–0.826) | 1.000 (1.000–1.000) | 0.819 (0.731–0.908) | 0.597 (0.484–0.711) | 0.778 (0.682–0.874) | 0.778 (0.682–0.874) |
| Specificity (95%CI) | 0.870 (0.790–0.949) | 0.957 (0.908–1.000) | 0.826 (0.737–0.916) | 0.870 (0.790–0.949) | 0.899 (0.827–0.970) | 0.841 (0.754–0.927) |
| Accuracy (95%CI) | 0.794 (0.792–0.797) | 0.979 (0.978–0.979) | 0.823 (0.821–0.825) | 0.730 (0.728–0.733) | 0.837 (0.835–0.839) | 0.809 (0.806–0.811) |
| PPV (95%CI) | 0.852 (0.763–0.941) | 0.960 (0.916–1.004) | 0.831 (0.744–0.918) | 0.827 (0.724–0.930) | 0.889 (0.811–0.966) | 0.836 (0.747–0.925) |
| NPV (95%CI) | 0.750 (0.655–0.845) | 1.000 (1.000–1.000) | 0.814 (0.723–0.905) | 0.674 (0.577–0.772) | 0.795 (0.705–0.884) | 0.784 (0.690–0.878) |
| *F1*-score | 0.782 | 0.980 | 0.825 | 0.694 | 0.830 | 0.806 |
| Brier score | 0.158 | 0.059 | 0.165 | 0.200 | 0.147 | 0.154 |

LR: logistic regression, XGBoost: extreme gradient boosting, RF: random forest, SVM: support vector machine, KNN: K-Nearest Neighbor, NB: Naive Bayes

**Table S7.** Robustness analysis of male and female Models

|  | AUC | Accuracy | Sensitivity | Specificity |
| --- | --- | --- | --- | --- |
| Male model (SVM) |  |  |  |  |
| sarcoidosis v.s. lung cancer (95%CI) | 0.919 (0.782–1.000) | 0.889 (0.747–0.956) | 0.700 (0.397–0.892) | 0.962 (0.811–0.993) |
| sarcoidosis v.s. MLNT (95%CI) | 0.914 (0.745–1.000) | 0.875 (0.690–0.957) | 0.700 (0.397–0.892) | 1.000 (0.785–1.000) |
| sarcoidosis v.s. Pneumoconiosis (95%CI) | 0.863 (0.670–1.000) | 0.778 (0.548–0.910) | 0.700 (0.397–0.892) | 0.875 (0.529–0.978) |
| 10-Fold Cross-Validation   (SD) | 0.922 (0.079) | 0.871 (0.061) | 0.658 (0.231) | 0.923 (0.063) |
| Female model (LR) |  |  |  |  |
| sarcoidosis v.s. lung cancer (95%CI) | 0.789 (0.634–0.944) | 0.744 (0.589–0.854) | 0.679 (0.493–0.821) | 0.909 (0.623–0.984) |
| sarcoidosis v.s. MLNT (95%CI) | 0.753 (0.589–0.916) | 0.714 (0.564–0.828) | 0.679 (0.493–0.821 ) | 0.786 (0.524–0.924) |
| 10-Fold Cross-Validation (SD) | 0.825 (0.110) | 0.752 (0.090) | 0.728 (0.229) | 0.757 (0.127) |

MLNT: Mediastinal Lymph Node Tuberculosis; Sensitivity was identical across comparisons because the same number of sarcoidosis cases was used in each comparison.

**Figure S1.** The process and results of variable selection using Boruta and LASSO (A/B/E: male group; C/D/F: female group). (A): result of Boruta for male group; (B): result of LASSO for male group; (C): result of Boruta for female group; (D): result of LASSO for female group; (E) and (F): ranking of coefficients for the final variables in the male and female groups, respectively.

**Figure S2.** Receiver operating characteristic curves (A and D), calibration plots (B and E), and decision curve analysis (C and F) of male and female models on the Training set. AUC: area under the curve; KNN: k-nearest neighbor; LR: logistic regression; RF: random forest; SVM: support vector machine; XGBoost: extreme gradient boosting; ROC: receiver operating characteristic

**Figure S3.** 10-Fold cross-validation results for the optimal male and female models

**Figure S4.** The feature importance and interpretation of the female model (based on the LR algorithm). (A) SHAP dependence plot: association between each feature and its SHAP value. point color represents the interaction effect between 2 features. (B) SHAP summary dot plot: relationship between feature values and SHAP values. The probability of pulmonary sarcoidosis classification increases with the SHAP value of a feature. (C) SHAP summary bar plot: ranking the importance of features. (D) SHAP force plot and (D) waterfall plot: feature contributions to an individual prediction. The impact of a feature increases with the magnitude of its SHAP value. GLU: glucose; CD3^+^: percentages of cluster of differentiation 3-positive T cell; CD8^+^: percentages of cluster of differentiation 3-positive T cell; sACE: serum angiotensin-converting enzyme; SHAP: Shapley Additive Explanations; TC: total cholesterol; TG/HDLC: triglyceride to high-density lipoprotein cholesterol ratio.

**Figure S5.** A Convenient online clinical practice application featuring SHAP explanations (male). sACE: serum angiotensin-converting enzyme; ESR: erythrocyte sedimentation rate; Lym#: lymphocyte count; RDW-SD: red cell distribution width standard deviation; TG/HDLC: triglyceride to high-density lipoprotein cholesterol ratio.
