# Supplementary material for: Differential Diagnosis of Early-Stage Pulmonary Sarcoidosis With Normal Serum Angiotensin-Converting Enzyme Activity Using Peripheral Blood Indicators: Machine Learning Models Development and Validation
Source: JMIR Med Inform. 2026 Oct 26;14:e86639. doi: 10.2196/86639 (PMC13749020; doi:10.2196/86639)
Supplement: Supplementary file 2 [file medinform_v14i1e86639_app2.docx]

library(readxl)

library(openxlsx)

library(dplyr)

library(ggplot2)

library(tidyverse)

library(patchwork)

library(tableone)

library(Matrix)

library(glmnet)

library(reportROC)

library(pROC)

library(mice)

library(zoo)

library(compareGroups)

library(MatchIt)

library(car)

library(e1071)

library(caret)

library(randomForest)

library(xgboost)

library(ggpubr)

library(Boruta)

library(FactoMineR)

library(factoextra)

library(class)

library(rmda)

library(dcurves)

library(fastshap)

library(shapviz)

library(pdp)

library(gridExtra)

library(car)

########Female models#######

quan <- read_excel("C:\\Users\\wlxy8\\Desktop\\课题\\肺结核鉴别诊断\\研二下诊断分析\\sace全重新做\\不平衡数量\\不平衡样本量.xlsx",

col_names = TRUE,sheet=1)

summary(dft$TID)

quan$TID <- as.factor(quan$TID)

set.seed(123)

train_index<-sample(2,nrow(quan),replace = T,prob = c(0.7,0.3))

df1<-quan[train_index==1,]

df2<-quan[train_index==2,]

write.xlsx(df2,"C:\\Users\\wlxy8\\Desktop\\课题\\肺结核鉴别诊断\\研二下诊断分析\\sace全重新做\\不平衡数量\\重新插补后的结果\\女验数据集.xlsx")

#######plot missing#######

names(df1)

md.pattern(df1[c(1:5,68:136)])

\# 定义函数：生成缺失值报告（仅包含有缺失的变量）

missing_values_report <- function(df) {

\# 计算每列的缺失值数量

na_count <- sapply(df, function(x) sum(is.na(x)))

\# 计算每列的缺失值百分比

na_percent <- round(na_count / nrow(df) * 100, 2)

\# 创建数据框并过滤无缺失的变量

result <- data.frame(

Variable = names(na_count),

Missing_Count = na_count,

Missing_Percent = na_percent

)

result <- result[result$Missing_Count > 0, ] # 只保留有缺失的行

\# 按缺失数量降序排序

result <- result[order(-result$Missing_Count), ]

\# 重置行号（可选）

rownames(result) <- NULL

return(result)

}

qstabfjj <- missing_values_report(df2[c(1:5,68:136)])

write.xlsx(qstabfjj,"C:\\Users\\wlxy8\\Desktop\\课题\\肺结核鉴别诊断\\研二下诊断分析\\sace全重新做\\不平衡数量\\重新插补后的结果\\女验缺失值.xlsx")

####Median imputation#######

names(df1)

df11 <- df1[c(1:5,68:136)]

df22 <- df2[c(1:5,68:136)]

numeric_cols <- sapply(df11, is.numeric) &

!names(df11) %in% "Species"

train_medians <- df11 %>%

select(where(is.numeric)) %>%

summarise(across(everything(), median, na.rm = TRUE))

cat("训练集各特征中位数:\n")

print(train_medians)

\# Fill function: Fills the data frame using the median vector passed as an argument

fill_median <- function(data, medians) {

for (col in names(medians)) {

if (col %in% names(data)) {

na_idx <- is.na(data[[col]])

data[[col]][na_idx] <- medians[[col]]

}

}

return(data)

}

dftrf <- fill_median(df11, train_medians)

dftef	<- fill_median(df22, train_medians) # Median of the training set

f1<-function(x){

return((x-min(x)) / (max(x)-min(x)))

}

tb<- descrTable(ID~.,data=dftr_f,method=NA)

export2csv(tb,file = "C:\\Users\\wlxy8\\Desktop\\课题\\肺结核鉴别诊断\\研二下诊断分析\\sace全重新做\\不平衡数量\\重新插补后的结果\\女训基线.csv")

names(dftrf)

summary(dftrf)

train_min <- apply(dftrf[, 6:74], 2, min, na.rm = TRUE)

train_max <- apply(dftrf[, 6:74], 2, max, na.rm = TRUE)

dftr_f1<-as.data.frame(lapply(dftrf[6:74],f1))

dftr_f <- cbind(dftrf[1:5],dftr_f1)

dfte_f1 <- as.data.frame(mapply(function(col, mn, mx) {

(col - mn) / (mx - mn)

}, dftef[, 6:74], train_min, train_max, SIMPLIFY = FALSE))

dfte_f <- cbind(dftef[, 1:5], dfte_f1)

set.seed(123)

boruta_model <- Boruta(ID~.,

data = dftr_f[c("ID","Lym0","Mono0","IgA",

"TP","ALB","GLU","SACE","TC","TGHDLC","APOB",

"APOE","CD3","CD3xx","CD8","CD4CD8", "FIBs","TTs",

"DDimerS","FDPS"

)],

pValue = 0.01,

mcAdj = TRUE,

maxRuns = 100,

doTrace = 0,

holdHistory = TRUE,

getImp = getImpRfZ)

Boruta::plotImpHistory(boruta_model)

boruta.variable.imp <- boruta.imp(boruta_model)

head(boruta.variable.imp)

print(boruta_model)

pdf("C:\\Users\\wlxy8\\Desktop\\课题\\肺结核鉴别诊断\\研二下诊断分析\\sace全重新做\\不平衡数量\\重新插补后的结果\\女boruta.pdf",

width = 9, height = 5)

plot(boruta_model,

sort=T,#按重要性排序

whichShadow=c(T,T,T),#是否画出影子特征，(max shadow,mean shadow,min shadow)

las=2,#x轴刻度标签90度旋转，1 or 2

xlab='')#x轴标题

dev.off()

final_model <- TentativeRoughFix(boruta_model)

print(final_model)

selected_features <- getSelectedAttributes(final_model, withTentative = FALSE)

print(selected_features)

y<-as.matrix(dftr_f[,1])

x<-as.matrix(dftr_f[c("Lym0","GLU" ,"SACE","TC","TGHDLC","APOB","CD3",

"CD8","DDimerS", "FDPS"

)])

lasso_model <- glmnet(x, y, family = "binomial",

alpha = 1) # 表示采用L1正则化，即Lasso回归。

max(lasso_model$lambda)

print(lasso_model)

#LASSO

plot(lasso_model,

xvar = "lambda")

set.seed(123)

cvfit=cv.glmnet(x,y,family = "binomial",alpha = 1,type.measure = "deviance",

nfolds = 5)

plot(cvfit)

la_min <- cvfit$lambda.min

la_lse <- cvfit$lambda.1se

l.coef2<-coef(cvfit$glmnet.fit,s=la_min,exact = F)

l.coef1<-coef(cvfit$glmnet.fit,s=la_lse,exact = F)

pdf("C:\\Users\\wlxy8\\Desktop\\课题\\肺结核鉴别诊断\\研二下诊断分析\\sace全重新做\\不平衡数量\\重新插补后的结果\\女lamo.pdf",

height = 6, width = 6

)

plot(lasso_model,

xvar = "lambda")

plot(cvfit)

dev.off()

\# Extract non-zero coefficients

coef_df <- data.frame(

variable = rownames(l.coef2)[-1],

coefficient =as.vector(l.coef2)[-1]

) %>%

filter(coefficient != 0) %>%

arrange(desc(abs(coefficient)))

\# Plot a coefficient bar chart

ggplot(coef_df, aes(x = reorder(variable, abs(coefficient)),

y = coefficient,fill=coefficient>0)) +

geom_bar(stat = "identity") +

coord_flip() +

labs(

x = "variables", y = "coefficient") +

theme_bw()+

theme(panel.grid=element_blank(),legend.position="none")

ggsave("C:\\Users\\wlxy8\\Desktop\\课题\\肺结核鉴别诊断\\研二下诊断分析\\sace全重新做\\不平衡数量\\重新插补后的结果\\女变量排序.pdf",

height = 6, width = 6

)

features <- coef_df$variable

formula <- as.formula(paste("ID ~", paste(features, collapse = "+")))

#######LR#########

lrmod<-glm(formula ,family="binomial",data = dftr_f)

vif(lrmod)

summary(lrmod)

flrpro<-predict(lrmod, newdata = dftr_f, type = "response")

roc.mod <- reportROC(gold =dftr_f$ID,predictor = flrpro,important = "se",plot=TRUE)

flrroc1 <- roc(dftr_f$ID,flrpro)

flrprote<-predict(lrmod, newdata = dfte_f, type = "response")

flrroc2 <- roc(dfte_f$ID,flrprote)

######## LR 10-folds Cross-validation########

library(pROC)

library(caret)

n_folds <- 10

fold_auc <- numeric(n_folds)

fold_sen <- numeric(n_folds)

fold_spe <- numeric(n_folds)

fold_acc <- numeric(n_folds)

\# 确保结果变量为数值 0/1

dftr_f$ID <- as.numeric(as.character(dftr_f$ID))

\# 创建分层10折索引

set.seed(123) # 固定随机种子，保证可重复

folds <- createFolds(dftr_f$ID, k = n_folds, list = TRUE)

for (i in 1:n_folds) {

val_idx <- folds[[i]]

tr_idx <- setdiff(1:nrow(dftr_f), val_idx)

tr_fold <- dftr_f[tr_idx, ]

val_fold <- dftr_f[val_idx, ]

\# 1. 在训练集上拟合模型

mod <- glm(formula, data = tr_fold, family = "binomial")

\# 2. 用训练集预测概率（用于确定阈值）

prob_train <- predict(mod, newdata = tr_fold, type = "response")

\# 用验证集预测概率（用于评估）

prob_val <- predict(mod, newdata = val_fold, type = "response")

\# 3. 仅在训练集上计算最佳阈值（约登指数）

roc_train <- roc(tr_fold$ID, prob_train, quiet = TRUE)

best_thresh <- coords(roc_train, "best", ret = "threshold",

best.method = "youden")$threshold[1]

\# 4. 用训练集得到的阈值对验证集进行分类

pred_class <- factor(ifelse(prob_val >= best_thresh, 1, 0), levels = c(0, 1))

true_class <- factor(val_fold$ID, levels = c(0, 1))

\# 5. 计算验证集的性能指标

cm <- confusionMatrix(pred_class, true_class, positive = "1")

fold_sen[i] <- cm$byClass["Sensitivity"]

fold_spe[i] <- cm$byClass["Specificity"]

fold_acc[i] <- cm$overall["Accuracy"]

\# 6. 计算验证集的 AUC（AUC 不需要阈值，故可直接用验证集概率）

roc_val <- roc(val_fold$ID, prob_val, quiet = TRUE)

fold_auc[i] <- auc(roc_val)

}

\# 输出平均性能

cat("\n========== 10折交叉验证结果（LR） ==========\n")

cat("平均 AUC	:", round(mean(fold_auc), 4), "±", round(sd(fold_auc), 4), "\n")

cat("平均灵敏度	:", round(mean(fold_sen), 4), "±", round(sd(fold_sen), 4), "\n")

cat("平均特异度	:", round(mean(fold_spe), 4), "±", round(sd(fold_spe), 4), "\n")

cat("平均准确度	:", round(mean(fold_acc), 4), "±", round(sd(fold_acc), 4), "\n")

\# ========== 箱线图（指标分布） ==========

df_metrics <- data.frame(

Fold = factor(rep(1:n_folds, 4)),

Metric = rep(c("AUC", "Sensitivity", "Specificity", "Accuracy"), each = n_folds),

Value = c(fold_auc, fold_sen, fold_spe, fold_acc)

)

p_box <- ggplot(df_metrics, aes(x = Metric, y = Value, fill = Metric)) +

geom_jitter(width = 0.2, alpha = 0.5) +

geom_boxplot(width = 0.3, fill = "white", outlier.shape = NA) +

stat_summary(fun = mean, geom = "point", shape = 18, size = 2, color = "darkred") +

labs(title = "", x = "", y = "") +

theme_classic(base_size = 10) +

theme(legend.position = "none",

axis.text.x = element_text(angle = 45, hjust = 1))

print(p_box)

ggsave("C:\\Users\\wlxy8\\Desktop\\课题\\肺结核鉴别诊断\\研二下诊断分析\\sace全重新做\\不平衡数量\\重新插补后的结果\\女LR交叉验证.pdf",

width = 4, height = 3, dpi = 300)

\####

###########XGB#########

library(Matrix)

set.seed(123)

ftd1 = data.matrix(dftr_f[,features])

ftd2 = Matrix(ftd1,sparse = T)

xgbftr = list(data = ftd2, y=as.numeric(as.character(dftr_f$ID)));xgbftr

xgbftr = xgb.DMatrix(data=xgbftr$data,label = xgbftr$y)

\# validation

fted1 = data.matrix(dfte_f[,features])

fted2 = Matrix(fted1,sparse = T)

xgbfte = list(data = fted2, y=as.numeric(as.character(dfte_f$ID)))

xgbfte = xgb.DMatrix(data=xgbfte$data,label = xgbfte$y)

param_grid <- expand.grid(

max_depth = c(2, 3, 4),

eta = c(0.01, 0.05, 0.1),

subsample = c(0.6, 0.8),

colsample_bytree = c(0.6, 0.8),

min_child_weight = c(3, 5),

gamma = c(0, 0.5),

lambda = c(1, 2), # L2正则

alpha = c(0.5, 1)	# L1正则

)

\# 存储结果

cv_results <- data.frame()

set.seed(123)

for (i in 1:nrow(param_grid)) {

params <- list(

objective = "binary:logistic",

eval_metric = "auc",

max_depth = param_grid$max_depth[i],

eta = param_grid$eta[i],

subsample = param_grid$subsample[i],

colsample_bytree = param_grid$colsample_bytree[i],

min_child_weight = param_grid$min_child_weight[i],

gamma = param_grid$gamma[i],

lambda = param_grid$lambda[i],

alpha = param_grid$alpha[i]

)

cv <- xgb.cv(

params = params,

data = xgbftr,

nrounds = 200,

nfold = 5,

early_stopping_rounds = 20,

verbose = 0,

prediction = FALSE

)

best_nrounds <- cv$best_iteration

best_auc <- cv$evaluation_log[best_nrounds, test_auc_mean]

cv_results <- rbind(cv_results, data.frame(

params,

best_nrounds = best_nrounds,

cv_auc = best_auc

))

}

\# 选出最佳参数组合

best_row <- cv_results[which.max(cv_results$cv_auc), ]

best_params <- list(

objective = "binary:logistic",

eval_metric = "auc",

max_depth = best_row$max_depth,

eta = best_row$eta,

subsample = best_row$subsample,

colsample_bytree = best_row$colsample_bytree,

min_child_weight = best_row$min_child_weight,

gamma = best_row$gamma,

lambda = best_row$lambda,

alpha = best_row$alpha

)

best_nrounds <- best_row$best_nrounds

set.seed(123)

fxgb.mod<- xgb.train(

params = best_params,

data = xgbftr,

nrounds = best_nrounds,

verbose = 1

)

\# 第5步：预测

fxgb.pre = predict(fxgb.mod,newdata =xgbftr,type = "prob")

fxgb.roc.tr <- roc(dftr_f$ID,fxgb.pre)

fxgb.prete <- predict(fxgb.mod,newdata =xgbfte,type = "prob")

fxgb.roc.te <- roc(dfte_f$ID,as.numeric(fxgb.prete))

##############RF##############

unique(dftr_m$ID)

set.seed(123)

frfx <- dftr_f[,features]

frfy <- as.factor(dftr_f$ID)

library(caret)

library(ranger)

set.seed(123)

\# Define the tuning grid: only mtry varies

tuneGrid_mtry <- expand.grid(

mtry = c(2, 3, 4, 5, 6),

splitrule = c("gini", "extratrees"),

min.node.size = c(1, 5, 10)

)

ctrl <- trainControl(method = "cv", number = 5, verbose = FALSE)

\# Train the model (set `num.trees` to 500, since we are only tuning `mtry` at this stage)

model_mtry <- train(

x = frfx,

y = frfy,

method = "ranger",

tuneGrid = tuneGrid_mtry,

trControl = ctrl,

num.trees = 500,

importance = "impurity"

)

\# 查看 mtry 调优结果

print(model_mtry)

\# 提取最佳参数

best_mtry <- model_mtry$bestTune$mtry

best_splitrule <- as.character(model_mtry$bestTune$splitrule)

best_min.node.size <- model_mtry$bestTune$min.node.size

cat("\n最佳 mtry:", best_mtry,

" | splitrule:", best_splitrule,

" | min.node.size:", best_min.node.size, "\n")

\# 3. 第二步：固定最佳 mtry 等参数，调优 num.trees（即 ntree）

\# 定义 num.trees 候选值

num.trees_list <- c(200, 300, 400, 500, 600)

\# 固定调优网格（只包含一个组合）

tuneGrid_fixed <- expand.grid(

mtry = best_mtry,

splitrule = best_splitrule,

min.node.size = best_min.node.size

)

results <- data.frame(num.trees = integer(), Accuracy = numeric())

\# Tuning num.trees

for (nt in num.trees_list) {

set.seed(123)

model_nt <- train(

x = frfx,

y = frfy,

method = "ranger",

tuneGrid = tuneGrid_fixed,

trControl = ctrl,

num.trees = nt,

importance = "impurity"

)

acc <- max(model_nt$results$Accuracy)

results <- rbind(results, data.frame(num.trees = nt, Accuracy = acc))

cat("num.trees =", nt, " | CV Accuracy =", round(acc, 4), "\n")

}

print(results)

\# num.trees

best_ntree <- results$num.trees[which.max(results$Accuracy)]

cat("\n最佳 num.trees:", best_ntree,

"，对应 CV 准确率:", round(max(results$Accuracy), 4), "\n")

\# 4. Train the final model using the optimal parameters

set.seed(123)

f_rf_model <- ranger(

x = frfx,

y = frfy,

mtry = best_mtry,

splitrule = best_splitrule,

min.node.size = best_min.node.size,

num.trees = best_ntree,

importance = "impurity",

probability = TRUE	# 与 randomForest 的 bootstrap 一致

)

print(f_rf_model)

importance_scores <- importance(f_rf_model)

names(dftr_f)

f_rftrpre <- predict(f_rf_model,data = dftr_f)

f_rftrpro <- predict(f_rf_model,data = dftr_f,type = "response")$predictions[, 2]

f_rf_roctr <- roc(as.numeric(as.character(dftr_f$ID)),f_rftrpro)

f_rftepro <- predict(f_rf_model,data = dfte_f,type="response")$predictions[, 2]

f_rfprete <- predict(f_rf_model,data = dfte_f)

f_rf_rocte <- roc(as.numeric(as.character(dfte_f$ID)),f_rftepro)

###SVM#####

str(dftr_f)

fsvmx <- dftr_f[,features]

fsvmy <- as.factor(dftr_f$ID)

set.seed(123)

tuned <- e1071::tune.svm(

x = fsvmx, y = fsvmy,

cost = 10^(-1:3), gamma = 10^(-3:1),

kernel = "radial", type = "C-classification",

tunecontrol = tune.control(sampling = "cross", cross = 5)

)

print(tuned$best.parameters)

best_cost <- tuned$best.parameters$cost

best_gamma <- tuned$best.parameters$gamma

f_svmmod <- svm(

x = fsvmx, y = fsvmy,

kernel = "radial", type = "C-classification",

cost = best_cost, gamma = best_gamma,

probability = TRUE

)

fsvmpre <- predict(f_svmmod,newdata = dftr_f[, features],probability = T)

fsvmprotr <- attr(fsvmpre,"probabilities")[,2]

fsvmprete <- predict(f_svmmod,newdata = dfte_f[,features],probability = T)

fsvmprote <- attr(fsvmprete,"probabilities")[,2]

f_svmroctr <- roc(dftr_f$ID,fsvmprotr)

f_svmrocte <- roc(dfte_f$ID,fsvmprote)

reportROC(gold =dfte_f$ID,predictor = fsvmprote)

####KNN#####

\# 2. find K

unique(dftr_f$ID0)

dftr_f$ID0 <- factor(dftr_f$ID,

levels = c(0, 1),

labels = c("case0", "case1"))

dfte_f$ID0 <- factor(dfte_f$ID,

levels = c(0, 1),

labels = c("case0", "case1"))

fknnctrl <- trainControl(

method = "cv",

number = 10, # 10 fold cv

classProbs = TRUE,

summaryFunction = twoClassSummary,

savePredictions = "final"

)

tuneGrid <- expand.grid(k = 1:50) # K from 1 to 50

\# 4. train KNN

set.seed(123)

fknnmod <- train(

x = dftr_f[,features],

y = dftr_f$ID0,

method = "knn",

trControl = fknnctrl,

tuneGrid = tuneGrid,

metric = "ROC"

)

\# 5. 查看最佳K值

print(fknnmod)

best_k <- fknnmod$bestTune$k

cat("最佳K值:", best_k, "\n")

fknnpretr <- predict(fknnmod, newdata = dftr_f, type = "prob")

fknnprotr <- fknnpretr$case1

fknnprete <- predict(fknnmod, newdata = dfte_f, type = "prob")

fknnprote <- fknnprete$case1

f_knnroctr <- roc(dftr_f$ID,fknnpretr$case1)

f_knnrocte <- roc(dfte_f$ID,fknnprete$case1)

########NB##########

fnbmod <- naiveBayes(formula,

data = dftr_f,laplace =1 )

fnbpretr <- predict(fnbmod,dftr_f,type="raw")

fnbprotr <- fnbpretr[,2]

fnbprete <- predict(fnbmod,dfte_f,type="raw")

fnbprote <- fnbprete[,2]

f_nbroctr <- roc(dftr_f$ID,fnbpretr[,2])

f_nbrocte <- roc(dfte_f$ID,fnbprete[,2])

#######ROC###########

roc_list <- list(

LR = flrroc1,

XGBoost = fxgb.roc.tr,

RF = f_rf_roctr,

KNN = f_knnroctr,

SVM = f_svmroctr,

`Naive Bayes` = f_nbroctr

)

\# 提取AUC和95% CI

roc_labels <- lapply(names(roc_list), function(model) {

roc_obj <- roc_list[[model]]

auc_value <- as.numeric(auc(roc_obj))

ci_value <- as.numeric(ci.auc(roc_obj))

data.frame(

Model = model,

AUC = auc_value,

CI_lower = ci_value[1],

CI_upper = ci_value[3]

)

})

roc_labels <- do.call(rbind, roc_labels)

\# 自动生成标签

roc_labels$label <- paste0(

roc_labels$Model,

": AUC = ",

sprintf("%.3f", roc_labels$AUC),

" (",

sprintf("%.3f", roc_labels$CI_lower),

" to ",

sprintf("%.3f", roc_labels$CI_upper),

")"

)

model_colors <- c(

"LR" = "#fd7446",

"XGBoost" = "#709ae1",

"RF" = "#8a9197",

"KNN" = "#d2af81",

"SVM" = "#fed439",

"Naive Bayes" = "#d5e4a2"

)

\# 自动设置文字位置

roc_labels$x <- 0.5

roc_labels$y <- seq(

0.25,

0,

length.out = nrow(roc_labels)

)

f_trainroc <- ggroc(

roc_list,

legacy.axes = TRUE,

size = 1

) +

\# 对角线

geom_segment(

aes(

x = 0,

xend = 1,

y = 0,

yend = 1

),

color = "darkgrey",

linetype = "dashed"

) +

\# ROC曲线颜色

scale_colour_manual(

values = model_colors

) +

\# 动态添加AUC和95%CI

geom_text(

data = roc_labels,

aes(

x = x,

y = y,

label = label,

color = Model

),

inherit.aes = FALSE,

size = 4,

hjust = 0,

vjust = 0,

family = "sans"

) +

theme_bw() +

theme(

panel.grid = element_blank(),

text = element_text(family = "sans"),

legend.position = "none",

plot.title = element_text(hjust = 0.5)

)

ggsave("C:\\Users\\wlxy8\\Desktop\\课题\\肺结核鉴别诊断\\研二下诊断分析\\sace全重新做\\不平衡数量\\重新插补后的结果\\女ROC训练集2.pdf",width=7,height=7,dpi=300)

roc_list2 <- list(

LR = flrroc2,

XGBoost = fxgb.roc.te,

RF = f_rf_rocte,

KNN = f_knnrocte,

SVM = f_svmrocte,

`Naive Bayes` = f_nbrocte

)

\# 提取AUC和95% CI

roc_labels2 <- lapply(names(roc_list2), function(model) {

roc_obj <- roc_list2[[model]]

auc_value <- as.numeric(auc(roc_obj))

ci_value <- as.numeric(ci.auc(roc_obj))

data.frame(

Model = model,

AUC = auc_value,

CI_lower = ci_value[1],

CI_upper = ci_value[3]

)

})

roc_labels2 <- do.call(rbind, roc_labels2)

\# 自动生成标签

roc_labels2$label <- paste0(

roc_labels2$Model,

": AUC = ",

sprintf("%.3f", roc_labels2$AUC),

" (",

sprintf("%.3f", roc_labels2$CI_lower),

" to ",

sprintf("%.3f", roc_labels2$CI_upper),

")"

)

\# 自动设置文字位置

roc_labels2$x <- 0.5

roc_labels2$y <- seq(

0.25,

0,

length.out = nrow(roc_labels2)

)

f_testroc <- ggroc(

roc_list2,

legacy.axes = TRUE,

size = 1

) +

\# 对角线

geom_segment(

aes(

x = 0,

xend = 1,

y = 0,

yend = 1

),

color = "darkgrey",

linetype = "dashed"

) +

\# ROC曲线颜色

scale_colour_manual(

values = model_colors

) +

\# 动态添加AUC和95% CI

geom_text(

data = roc_labels2,

aes(

x = x,

y = y,

label = label,

color = Model

),

inherit.aes = FALSE,

size = 4,

hjust = 0,

vjust = 0,

family = "sans"

) +

theme_bw() +

theme(

panel.grid = element_blank(),

text = element_text(family = "sans"),

legend.position = "none",

plot.title = element_text(hjust = 0.5)

)

ggsave("C:\\Users\\wlxy8\\Desktop\\课题\\肺结核鉴别诊断\\研二下诊断分析\\sace全重新做\\不平衡数量\\重新插补后的结果\\女ROC验证集2.pdf",width=7,height=7,dpi=300)

##########DCA##########

fdca_data <- data.frame(

outcome =as.numeric(as.character(dftr_f$ID)), # 真实结果（二分类变量）

LR = flrpro, # 逻辑回归预测概率

XGBoost=fxgb.pre,

RF = f_rftrpro, # 随机森林预测概率

KNN=fknnprotr,

SVM = fsvmprotr,# 支持向量机预测概率

NaiveBayes=fnbprotr

)

fdca_results <- dca(

data = fdca_data,

outcome~.,

thresholds = seq(0.01, 0.99, 0.01)

)

plot(fdca_results) +

theme_bw() +

labs(title = "Decision Curve Analysis (Female model)",

x = "Threshold probability",

y = "Net Benefit") +

scale_color_manual(values = c("darkgrey","grey","#fd7446","#709ae1","#8a9197","#d2af81","#fed439","#d5e4a2") )+

theme(legend.position = c(0.15,0.38),

panel.grid=element_blank(),

legend.key.size = unit(13, "pt"))

\# scale_color_brewer(palette = "Set1")

ggsave("C:\\Users\\wlxy8\\Desktop\\课题\\肺结核鉴别诊断\\研二下诊断分析\\sace全重新做\\不平衡数量\\重新插补后的结果\\女DCA训练集2.pdf",width=7,height=7,dpi=300)

#validation DCA

fdca_datate <- data.frame(

outcome =as.numeric(as.character(dfte_f$ID)), # 真实结果（二分类变量）

LR = flrprote, # 逻辑回归预测概率

XGBoost=fxgb.prete,

RF = f_rftepro, # 随机森林预测概率

KNN=fknnprote,

SVM = fsvmprote,# 支持向量机预测概率

NaiveBayes=fnbprote

)

fdca_teresults <- dca(

data = fdca_datate,

outcome~.,

thresholds = seq(0.01, 0.99, 0.01)

)

plot(fdca_teresults) +

theme_bw() +

labs(title = "Decision Curve Analysis (Female model)",

x = "Threshold probability",

y = "Net Benefit") +

scale_color_manual(values = c("darkgrey","grey","#fd7446","#709ae1","#8a9197","#d2af81","#fed439","#d5e4a2")) +

theme(legend.position = c(0.15,0.38),

panel.grid=element_blank(),

legend.key.size = unit(13, "pt"))

\# scale_color_brewer(palette = "Set1")

ggsave("C:\\Users\\wlxy8\\Desktop\\课题\\肺结核鉴别诊断\\研二下诊断分析\\sace全重新做\\不平衡数量\\重新插补后的结果\\女DCA验证集2.pdf",width=7,height=7,dpi=300)

######Calculate the discrimination index(Female)###########

calc_metrics_at_cutoff <- function(gold, prob, cutoff) {

pred <- ifelse(prob >= cutoff, 1, 0)

\# Construct a confusion matrix

tp <- sum(pred == 1 & gold == 1)

tn <- sum(pred == 0 & gold == 0)

fp <- sum(pred == 1 & gold == 0)

fn <- sum(pred == 0 & gold == 1)

n <- length(gold)

\# (Sensitivity) = TP / (TP+FN)

sen <- tp / (tp + fn)

\# (Specificity) = TN / (TN+FP)

spe <- tn / (tn + fp)

\# (Accuracy) = (TP+TN)/n

acc <- (tp + tn) / n

\# (PPV) = TP / (TP+FP)

ppv <- tp / (tp + fp)

\# (NPV) = TN / (TN+FN)

npv <- tn / (tn + fn)

wilson_ci <- function(x, n) {

if (n == 0) return(c(NA, NA))

p <- x / n

z <- qnorm(0.975)

denom <- 1 + z^2/n

centre <- p + z^2/(2*n)

half <- z * sqrt((p*(1-p) + z^2/(4*n)) / n)

c((centre - half)/denom, (centre + half)/denom)

}

sen_ci <- wilson_ci(tp, tp+fn)

spe_ci <- wilson_ci(tn, tn+fp)

acc_ci <- wilson_ci(tp+tn, n)

ppv_ci <- wilson_ci(tp, tp+fp)

npv_ci <- wilson_ci(tn, tn+fn)

return(data.frame(

Cutoff = cutoff,

Sensitivity = sen, Sensitivity_low = sen_ci[1], Sensitivity_up = sen_ci[2],

Specificity = spe, Specificity_low = spe_ci[1], Specificity_up = spe_ci[2],

Accuracy = acc, Accuracy_low = acc_ci[1], Accuracy_up = acc_ci[2],

PPV = ppv, PPV_low = ppv_ci[1], PPV_up = ppv_ci[2],

NPV = npv, NPV_low = npv_ci[1], NPV_up = npv_ci[2]

))

}

###train

MLmodels <- list(

LR = flrpro, RF = f_rftrpro, XGB = fxgb.pre,

KNN = fknnprotr, SVM = fsvmprotr, NB = fnbprotr

)

results_list <- list()

cutoff_list <- list()

for (name in names(MLmodels)) {

res <- reportROC(gold = dftr_f$ID, predictor = MLmodels[[name]],

important = "se", plot = FALSE)

\# Extract the optimal cutoff calculated on the training set (for use on the validation set)

cutoff_train <- as.numeric(res$Cutoff)

cutoff_list[[name]] <- cutoff_train

results_list[[name]] <- data.frame(

Model = name,

Cutoff = round(cutoff_train, 3),

AUC = round(as.numeric(res$AUC), 3),

AUC_low = round(as.numeric(res$AUC.low), 3),

AUC_up = round(as.numeric(res$AUC.up), 3),

Sensitivity = round(as.numeric(res$SEN), 3),

Sensitivity_low = round(as.numeric(res$SEN.low), 3),

Sensitivity_up = round(as.numeric(res$SEN.up), 3),

Specificity = round(as.numeric(res$SPE), 3),

Specificity_low = round(as.numeric(res$SPE.low), 3),

Specificity_up = round(as.numeric(res$SPE.up), 3),

Accuracy = round(as.numeric(res$ACC), 3),

Accuracy_low = round(as.numeric(res$ACC.low), 3),

Accuracy_up = round(as.numeric(res$ACC.up), 3),

PPV = round(as.numeric(res$PPV), 3),

PPV_low = round(as.numeric(res$PPV.low), 3),

PPV_up = round(as.numeric(res$PPV.up), 3),

NPV = round(as.numeric(res$NPV), 3),

NPV_low = round(as.numeric(res$NPV.low), 3),

NPV_up = round(as.numeric(res$NPV.up), 3)

)

}

final_res_tr <- do.call(rbind, results_list)

write.xlsx(final_res_tr, "C:\\Users\\wlxy8\\Desktop\\课题\\肺结核鉴别诊断\\研二下诊断分析\\sace全重新做\\不平衡数量\\重新插补后的结果\\女训区分度4.xlsx")

###validation

MLmodels2 <- list(

LR = flrprote, RF = f_rftepro, XGB = fxgb.prete,

KNN = fknnprote, SVM = fsvmprote, NB = fnbprote

)

results_list2 <- list()

for (name in names(MLmodels2)) {

roc_obj <- roc(dfte_f$ID, MLmodels2[[name]], quiet = TRUE)

auc_val <- auc(roc_obj)

auc_ci <- ci.auc(roc_obj, method = "delong")

\# 2. 使用训练集上得到的 cutoff 计算验证集的二分类指标

cutoff_train <- cutoff_list[[name]] # 从训练集传入

metrics_val <- calc_metrics_at_cutoff(gold = dfte_f$ID,

prob = MLmodels2[[name]],

cutoff = cutoff_train)

results_list2[[name]] <- data.frame(

Model = name,

Cutoff_train = round(cutoff_train, 3),

AUC = round(auc_val, 3),

AUC_low = round(auc_ci[1], 3),

AUC_up = round(auc_ci[3], 3),

Sensitivity = round(metrics_val$Sensitivity, 3),

Sensitivity_low = round(metrics_val$Sensitivity_low, 3),

Sensitivity_up = round(metrics_val$Sensitivity_up, 3),

Specificity = round(metrics_val$Specificity, 3),

Specificity_low = round(metrics_val$Specificity_low, 3),

Specificity_up = round(metrics_val$Specificity_up, 3),

Accuracy = round(metrics_val$Accuracy, 3),

Accuracy_low = round(metrics_val$Accuracy_low, 3),

Accuracy_up = round(metrics_val$Accuracy_up, 3),

PPV = round(metrics_val$PPV, 3),

PPV_low = round(metrics_val$PPV_low, 3),

PPV_up = round(metrics_val$PPV_up, 3),

NPV = round(metrics_val$NPV, 3),

NPV_low = round(metrics_val$NPV_low, 3),

NPV_up = round(metrics_val$NPV_up, 3)

)

}

final_res_te <- do.call(rbind, results_list2)

write.xlsx(final_res_te, "C:\\Users\\wlxy8\\Desktop\\课题\\肺结核鉴别诊断\\研二下诊断分析\\sace全重新做\\不平衡数量\\重新插补后的结果\\女验区分度4.xlsx")

#flrroc2,fxgb.roc.te,f_rf_rocte,f_svmrocte,f_knnrocte,f_nbrocte

best_cutoff <- coords(flrroc1, "best", best.method = "youden")#计算最佳截断点

best_cutoff

pred_class <- ifelse(flrpro >= best_cutoff$threshold, 1, 0)

conf_matrix <- table(Actual = dftr_f$ID, Pred = pred_class)

flrcmtr <- confusionMatrix( as.factor(pred_class),as.factor(dftr_f$ID),

positive = "1", mode = "everything")

pred_class2 <- ifelse(flrprote >= best_cutoff$threshold, 1, 0)

conf_matrix <- table(Actual = dfte_f$ID, Pred = pred_class2)

flrcmte <- confusionMatrix( as.factor(pred_class2),as.factor(dfte_f$ID),

positive = "1", mode = "everything")

best_cutoff <- coords(fxgb.roc.tr, "best", best.method = "youden")#计算最佳截断点

best_cutoff

pred_class <- ifelse(fxgb.pre >= best_cutoff$threshold, 1, 0)

conf_matrix <- table(Actual = dftr_f$ID, Pred = pred_class)

fxgbcmtr <- confusionMatrix(as.factor(pred_class),as.factor(dftr_f$ID),

positive = "1", mode = "everything")

pred_class2 <- ifelse(fxgb.prete >= best_cutoff$threshold, 1, 0)

conf_matrix2 <- table(Actual = dfte_f$ID, Pred = pred_class2)

fxgbcmte <- confusionMatrix(as.factor(pred_class2),as.factor(dfte_f$ID),

positive = "1", mode = "everything")

best_cutoff <- coords(f_rf_roctr, "best", best.method = "youden")#计算最佳截断点

best_cutoff

pred_class <- ifelse(f_rftrpro >= best_cutoff$threshold, 1, 0)

conf_matrix <- table(Actual = dftr_f$ID, Pred = pred_class)

frfcmtr <- confusionMatrix( as.factor(pred_class),as.factor(dftr_f$ID),

positive = "1", mode = "everything")

pred_class2 <- ifelse(f_rftepro >= best_cutoff$threshold, 1, 0)

conf_matrix2 <- table(Actual = dfte_f$ID, Pred = pred_class2)

frfcmte <- confusionMatrix( as.factor(pred_class2),as.factor(dfte_f$ID),

positive = "1", mode = "everything")

best_cutoff <- coords(f_svmroctr, "best", best.method = "youden")#计算最佳截断点

best_cutoff

pred_class <- ifelse(fsvmprotr >= best_cutoff$threshold, 1, 0)

conf_matrix <- table(Actual = dftr_f$ID, Pred = pred_class)

fsvmcmtr <- confusionMatrix( as.factor(pred_class),as.factor(dftr_f$ID),

positive = "1", mode = "everything")

pred_class2 <- ifelse(fsvmprote >= best_cutoff$threshold, 1, 0)

conf_matrix2 <- table(Actual = dfte_f$ID, Pred = pred_class2)

fsvmcmte <- confusionMatrix( as.factor(pred_class2),as.factor(dfte_f$ID),

positive = "1", mode = "everything")

best_cutoff <- coords(f_knnroctr, "best", best.method = "youden")#计算最佳截断点

best_cutoff

pred_class <- ifelse(fknnprotr >= best_cutoff$threshold, 1, 0)

conf_matrix <- table(Actual = dftr_f$ID, Pred = pred_class)

fknncmtr <- confusionMatrix( as.factor(pred_class),as.factor(dftr_f$ID),

positive = "1", mode = "everything")

pred_class2 <- ifelse(fknnprote >= best_cutoff$threshold, 1, 0)

conf_matrix2 <- table(Actual = dfte_f$ID, Pred = pred_class2)

fknncmte <- confusionMatrix( as.factor(pred_class2),as.factor(dfte_f$ID),

positive = "1", mode = "everything")

best_cutoff <- coords(f_nbroctr, "best", best.method = "youden")#计算最佳截断点

best_cutoff

pred_class <- ifelse(fnbprotr >= best_cutoff$threshold, 1, 0)

conf_matrix <- table(Actual = dftr_f$ID, Pred = pred_class)

fnbcmtr <- confusionMatrix( as.factor(pred_class),as.factor(dftr_f$ID),

positive = "1", mode = "everything")

pred_class2 <- ifelse(fnbprote >= best_cutoff$threshold, 1, 0)

conf_matrix2 <- table(Actual = dfte_f$ID, Pred = pred_class2)

fnbcmte <- confusionMatrix( as.factor(pred_class2),as.factor(dfte_f$ID),

positive = "1", mode = "everything")

fcmtr_result <- data.frame(lr <- c(

round(flrcmtr$overall["Accuracy"], 4),

round(flrcmtr$byClass["Sensitivity"], 4),

round(flrcmtr$byClass["Specificity"], 4),

round(flrcmtr$byClass["Precision"], 4),

round(flrcmtr$byClass["Recall"], 4),

round(flrcmtr$byClass["F1"], 4),

round(flrcmtr$byClass["Pos Pred Value"], 4),

round(flrcmtr$byClass["Neg Pred Value"], 4),

round(flrcmtr$overall["Kappa"], 4),

round(flrcmtr$byClass["Balanced Accuracy"], 4)),

xgb <- c(

round(fxgbcmtr$overall["Accuracy"], 4),

round(fxgbcmtr$byClass["Sensitivity"], 4),

round(fxgbcmtr$byClass["Specificity"], 4),

round(fxgbcmtr$byClass["Precision"], 4),

round(fxgbcmtr$byClass["Recall"], 4),

round(fxgbcmtr$byClass["F1"], 4),

round(fxgbcmtr$byClass["Pos Pred Value"], 4),

round(fxgbcmtr$byClass["Neg Pred Value"], 4),

round(fxgbcmtr$overall["Kappa"], 4),

round(fxgbcmtr$byClass["Balanced Accuracy"], 4)),

rf <- c(

round(frfcmtr$overall["Accuracy"], 4),

round(frfcmtr$byClass["Sensitivity"], 4),

round(frfcmtr$byClass["Specificity"], 4),

round(frfcmtr$byClass["Precision"], 4),

round(frfcmtr$byClass["Recall"], 4),

round(frfcmtr$byClass["F1"], 4),

round(frfcmtr$byClass["Pos Pred Value"], 4),

round(frfcmtr$byClass["Neg Pred Value"], 4),

round(frfcmtr$overall["Kappa"], 4),

round(frfcmtr$byClass["Balanced Accuracy"], 4)),

svm <- c(

round(fsvmcmtr$overall["Accuracy"], 4),

round(fsvmcmtr$byClass["Sensitivity"], 4),

round(fsvmcmtr$byClass["Specificity"], 4),

round(fsvmcmtr$byClass["Precision"], 4),

round(fsvmcmtr$byClass["Recall"], 4),

round(fsvmcmtr$byClass["F1"], 4),

round(fsvmcmtr$byClass["Pos Pred Value"], 4),

round(fsvmcmtr$byClass["Neg Pred Value"], 4),

round(fsvmcmtr$overall["Kappa"], 4),

round(fsvmcmtr$byClass["Balanced Accuracy"], 4)),

knn <- c(

round(fknncmtr$overall["Accuracy"], 4),

round(fknncmtr$byClass["Sensitivity"], 4),

round(fknncmtr$byClass["Specificity"], 4),

round(fknncmtr$byClass["Precision"], 4),

round(fknncmtr$byClass["Recall"], 4),

round(fknncmtr$byClass["F1"], 4),

round(fknncmtr$byClass["Pos Pred Value"], 4),

round(fknncmtr$byClass["Neg Pred Value"], 4),

round(fknncmtr$overall["Kappa"], 4),

round(fknncmtr$byClass["Balanced Accuracy"], 4)),

nb <- c(

round(fnbcmtr$overall["Accuracy"], 4),

round(fnbcmtr$byClass["Sensitivity"], 4),

round(fnbcmtr$byClass["Specificity"], 4),

round(fnbcmtr$byClass["Precision"], 4),

round(fnbcmtr$byClass["Recall"], 4),

round(fnbcmtr$byClass["F1"], 4),

round(fnbcmtr$byClass["Pos Pred Value"], 4),

round(fnbcmtr$byClass["Neg Pred Value"], 4),

round(fnbcmtr$overall["Kappa"], 4),

round(fnbcmtr$byClass["Balanced Accuracy"], 4))

)

write.csv(fcmtr_result,"C:\\Users\\wlxy8\\Desktop\\课题\\肺结核鉴别诊断\\研二下诊断分析\\sace全重新做\\不平衡数量\\重新插补后的结果\\女各指标结果训练集3.csv")

fcmte_result <- data.frame(lr <- c(

round(flrcmte$overall["Accuracy"], 4),

round(flrcmte$byClass["Sensitivity"], 4),

round(flrcmte$byClass["Specificity"], 4),

round(flrcmte$byClass["Precision"], 4),

round(flrcmte$byClass["Recall"], 4),

round(flrcmte$byClass["F1"], 4),

round(flrcmte$byClass["Pos Pred Value"], 4),

round(flrcmte$byClass["Neg Pred Value"], 4),

round(flrcmte$overall["Kappa"], 4),

round(flrcmte$byClass["Balanced Accuracy"], 4)),

xgb <- c(

round(fxgbcmte$overall["Accuracy"], 4),

round(fxgbcmte$byClass["Sensitivity"], 4),

round(fxgbcmte$byClass["Specificity"], 4),

round(fxgbcmte$byClass["Precision"], 4),

round(fxgbcmte$byClass["Recall"], 4),

round(fxgbcmte$byClass["F1"], 4),

round(fxgbcmte$byClass["Pos Pred Value"], 4),

round(fxgbcmte$byClass["Neg Pred Value"], 4),

round(fxgbcmte$overall["Kappa"], 4),

round(fxgbcmte$byClass["Balanced Accuracy"], 4)),

rf <- c(

round(frfcmte$overall["Accuracy"], 4),

round(frfcmte$byClass["Sensitivity"], 4),

round(frfcmte$byClass["Specificity"], 4),

round(frfcmte$byClass["Precision"], 4),

round(frfcmte$byClass["Recall"], 4),

round(frfcmte$byClass["F1"], 4),

round(frfcmte$byClass["Pos Pred Value"], 4),

round(frfcmte$byClass["Neg Pred Value"], 4),

round(frfcmte$overall["Kappa"], 4),

round(frfcmte$byClass["Balanced Accuracy"], 4)),

svm <- c(

round(fsvmcmte$overall["Accuracy"], 4),

round(fsvmcmte$byClass["Sensitivity"], 4),

round(fsvmcmte$byClass["Specificity"], 4),

round(fsvmcmte$byClass["Precision"], 4),

round(fsvmcmte$byClass["Recall"], 4),

round(fsvmcmte$byClass["F1"], 4),

round(fsvmcmte$byClass["Pos Pred Value"], 4),

round(fsvmcmte$byClass["Neg Pred Value"], 4),

round(fsvmcmte$overall["Kappa"], 4),

round(fsvmcmte$byClass["Balanced Accuracy"], 4)),

knn <- c(

round(fknncmte$overall["Accuracy"], 4),

round(fknncmte$byClass["Sensitivity"], 4),

round(fknncmte$byClass["Specificity"], 4),

round(fknncmte$byClass["Precision"], 4),

round(fknncmte$byClass["Recall"], 4),

round(fknncmte$byClass["F1"], 4),

round(fknncmte$byClass["Pos Pred Value"], 4),

round(fknncmte$byClass["Neg Pred Value"], 4),

round(fknncmte$overall["Kappa"], 4),

round(fknncmte$byClass["Balanced Accuracy"], 4)),

nb <- c(

round(fnbcmte$overall["Accuracy"], 4),

round(fnbcmte$byClass["Sensitivity"], 4),

round(fnbcmte$byClass["Specificity"], 4),

round(fnbcmte$byClass["Precision"], 4),

round(fnbcmte$byClass["Recall"], 4),

round(fnbcmte$byClass["F1"], 4),

round(fnbcmte$byClass["Pos Pred Value"], 4),

round(fnbcmte$byClass["Neg Pred Value"], 4),

round(fnbcmte$overall["Kappa"], 4),

round(fnbcmte$byClass["Balanced Accuracy"], 4))

)

write.csv(fcmte_result,"C:\\Users\\wlxy8\\Desktop\\课题\\肺结核鉴别诊断\\研二下诊断分析\\sace全重新做\\不平衡数量\\重新插补后的结果\\女各指标结果验证集3.csv")

####Calibration curve /train/female#####

calibration_data <- data.frame(

ID = as.numeric(as.character(dftr_f$ID)),

LR = flrpro,

XGBoost = fxgb.pre,

RF = f_rftrpro,

KNN = fknnprotr,

SVM = fsvmprotr,

NaiveBayes = fnbprotr

)

calibration_long <- calibration_data %>%

pivot_longer(cols = -ID, names_to = "model", values_to = "pred")

nbins <- 5

cal_curve_data <- calibration_long %>%

group_by(model) %>%

mutate(bin = ntile(pred, nbins)) %>% # 分成nbins组

group_by(model, bin) %>%

summarise(

mean_pred = mean(pred, na.rm = TRUE),

mean_obs = mean(ID, na.rm = TRUE),

.groups = "drop"

)

\# plot

jzqx <- ggplot(cal_curve_data, aes(x = mean_pred, y = mean_obs, color = model)) +

geom_line() +

geom_point() +

geom_abline(intercept = 0, slope = 1, linetype = "dashed", color = "gray") +

scale_color_manual(values = c("#fed439","#fd7446","#709ae1","#8a9197","#d2af81","#d5e4a2"))+

labs(x = "Predicted probability", y = "True probaility",

title = "Calibration Curves (Female models)",

color = "models") +

theme_bw()+

theme(panel.grid = element_blank())+

coord_equal()

ggsave("C:\\Users\\wlxy8\\Desktop\\课题\\肺结核鉴别诊断\\研二下诊断分析\\sace全重新做\\不平衡数量\\重新插补后的结果\\女校准曲线训练集2.pdf",width=7,height=7,dpi=300)

\# Brier score

model_names <- c("LR", "XGBoost", "RF", "KNN", "SVM", "NaiveBayes")

brier_scores <- data.frame(

Model = model_names,

BrierScore = sapply(model_names, function(m) {

mean((calibration_data[[m]] - as.numeric(as.character(calibration_data$ID)))^2)

})

)

print(brier_scores)

######Calibration curve/validation/female#########

calibration_data2 <- data.frame(

ID = dfte_f$ID, # 实际结果

LR = flrprote,

XGBoost = fxgb.prete,

RF = f_rftepro,

KNN = fknnprote,

SVM = fsvmprote,

NaiveBayes = fnbprote

)

calibration_long2 <- calibration_data2 %>%

pivot_longer(cols = -ID, names_to = "model", values_to = "pred")

cal_curve_data2 <- calibration_long2 %>%

group_by(model) %>%

mutate(bin = ntile(pred, nbins)) %>% # 分成nbins组

group_by(model, bin) %>%

summarise(

mean_pred = mean(pred, na.rm = TRUE),

mean_obs = mean(ID, na.rm = TRUE),

.groups = "drop"

)

\# plot

jzqx2 <- ggplot(cal_curve_data2, aes(x = mean_pred, y = mean_obs, color = model)) +

geom_line() +

geom_point() +

geom_abline(intercept = 0, slope = 1, linetype = "dashed", color = "gray") +

scale_color_manual(values = c("#fed439","#fd7446","#709ae1","#8a9197","#d2af81","#d5e4a2"))+

labs(x = "Predicted probability", y = "True probaility",

title = "Calibration Curves (Female models)",

color = "models") +

scale_y_continuous(limits = c(0,1),breaks = seq(0,1,0.25))+

theme_bw()+

theme(panel.grid = element_blank())+

coord_equal() # 使x和y轴比例相同，更易看出偏差

ggsave("C:\\Users\\wlxy8\\Desktop\\课题\\肺结核鉴别诊断\\研二下诊断分析\\sace全重新做\\不平衡数量\\重新插补后的结果\\女校准曲线验证集2.pdf",width=7,height=7,dpi=300)

\# Brier scores

brier_scores2 <- data.frame(

Model = model_names,

BrierScore = sapply(model_names, function(m) {

mean((calibration_data2[[m]] - as.numeric(as.character(calibration_data2$ID)))^2)

})

)

#########FEMALE/Differential Diagnosis of Sarcoidosis from Specific Control Diseases##########

cancer <- dftef %>%

filter(TID==1|

TID==3)

names(cancer)

cancer1 <- as.data.frame(mapply(function(col, mn, mx) {

(col - mn) / (mx - mn)

}, cancer[, 6:74], train_min, train_max, SIMPLIFY = FALSE))

cancer <- cbind(cancer[, 1:5], cancer1)

can_pro<-predict(lrmod, newdata = cancer, type = "response")

lr_cutoff <- coords(flrroc1, "best", best.method = "youden")#计算最佳截断点

pred_can <- ifelse(can_pro >= lr_cutoff$threshold, 1, 0)

can_cm <- confusionMatrix( as.factor(pred_can),as.factor(cancer$ID),

positive = "1", mode = "everything")

tub <- dftef %>%

filter(TID==1|

TID==0)

tub1 <- as.data.frame(mapply(function(col, mn, mx) {

(col - mn) / (mx - mn)

}, tub[, 6:74], train_min, train_max, SIMPLIFY = FALSE))

tub <- cbind(tub[, 1:5], tub1)

tub_pro<-predict(lrmod, newdata = tub, type = "response")

pred_tub <- ifelse(tub_pro >= lr_cutoff$threshold, 1, 0)

tub_cm <- confusionMatrix( as.factor(pred_tub),as.factor(tub$ID),

positive = "1", mode = "everything")

res_cancer <- calc_metrics_at_cutoff(

gold	= cancer$ID,

prob	= can_pro,

cutoff = lr_cutoff$threshold

)

res_cancer$Dataset <- "cancer"

res_tub <- calc_metrics_at_cutoff(

gold	= tub$ID,

prob	= tub_pro,

cutoff = lr_cutoff$threshold

)

res_tub$Dataset <- "tub"

\# 合并结果

obo_res <- rbind(res_cancer, res_tub)

write.csv(obo_res,"C:\\Users\\wlxy8\\Desktop\\课题\\肺结核鉴别诊断\\研二下诊断分析\\sace全重新做\\不平衡数量\\重新插补后的结果\\一对一检验结果\\女一对一.csv")

reportROC(cancer$ID,can_pro,plot = T)

######Female/SHAP########

library(kernelshap)

library(shapviz)

bg_data <- dfte_f[,features]

pred_fun <- function(object, newdata) {

predict(object, newdata = newdata, type = "response")

}

shap_results <- permshap(

object = lrmod, # 你的 glm 模型

X = bg_data, # 你想要解释的特征数据

bg_X = dfte_f, # 背景数据集

exact = FALSE	# 使用高效的近似算法

)

shp <- shapviz(shap_results)

sv_waterfall(shp, row_id = 12) +

#ggtitle("Waterfall Plot for First Sample") +

theme_bw()+

theme(panel.grid=element_blank())

ggsave("C:\\Users\\wlxy8\\Desktop\\课题\\肺结核鉴别诊断\\研二下诊断分析\\sace全重新做\\不平衡数量\\重新插补后的结果\\shap女LR瀑布图3.pdf",width=6,height=5,dpi=300)

sv_importance(shp, kind = "bee") + # 'bee'显示散点，'bar'为纯柱状

#ggtitle("SHAP Variable Importance") +

theme_bw()+

theme(panel.grid=element_blank())

ggsave("C:\\Users\\wlxy8\\Desktop\\课题\\肺结核鉴别诊断\\研二下诊断分析\\sace全重新做\\不平衡数量\\重新插补后的结果\\shap女LR蜂窝图3.pdf",width=6,height=5,dpi=300)

sv_importance(shp, kind = "bar") + # 'bee'显示散点，'bar'为纯柱状

#ggtitle("SHAP Variable Importance") +

theme_bw()+

theme(panel.grid=element_blank())

ggsave("C:\\Users\\wlxy8\\Desktop\\课题\\肺结核鉴别诊断\\研二下诊断分析\\sace全重新做\\不平衡数量\\重新插补后的结果\\shap女LR重要图3.pdf",width=6,height=5,dpi=300)

sv_force(shp, row_id = 12)

ggsave("C:\\Users\\wlxy8\\Desktop\\课题\\肺结核鉴别诊断\\研二下诊断分析\\sace全重新做\\不平衡数量\\重新插补后的结果\\shap女LR单个力图3.pdf",width=7,height=2,dpi=300)

features <- colnames(shp$X)

dep_plots <- lapply(features, function(feat) {

sv_dependence(shp, v = feat, alpha = 0.5) +

ggtitle(feat) +

theme_minimal() +

theme(plot.title = element_text(size = 10)

)

})

\# 拼接图形（例如 4 列）

combined_plot <- wrap_plots(dep_plots, ncol = 2)

pdf("C:\\Users\\wlxy8\\Desktop\\课题\\肺结核鉴别诊断\\研二下诊断分析\\sace全重新做\\不平衡数量\\重新插补后的结果\\shap女LR依赖图3.pdf",

width = 6, height = 7)

print(combined_plot)

dev.off()

######Male model##########

quan<- read_excel("C:\\Users\\wlxy8\\Desktop\\课题\\肺结核鉴别诊断\\研二下诊断分析\\sace全重新做\\不平衡数量\\不平衡样本量.xlsx",

col_names = TRUE,sheet=1)

summary(dftm$TID)

dftm$TID <- as.factor(dftm$TID)

set.seed(123)

train_index<-sample(2,nrow(quan),replace = T,prob = c(0.7,0.3))

df1 <- quan[train_index==1,]

df2 <- quan[train_index==2,]

write.xlsx(df1,"C:\\Users\\wlxy8\\Desktop\\课题\\肺结核鉴别诊断\\研二下诊断分析\\sace全重新做\\不平衡数量\\重新插补后的结果\\男训数据集.xlsx")

#######missing#######

names(dftr_m)

md.pattern(df1[c(1:5,65:138)])

missing_values_report <- function(df) {

na_count <- sapply(df, function(x) sum(is.na(x)))

na_percent <- round(na_count / nrow(df) * 100, 2)

result <- data.frame(

Variable = names(na_count),

Missing_Count = na_count,

Missing_Percent = na_percent

)

result <- result[result$Missing_Count > 0, ] # 只保留有缺失的行

result <- result[order(-result$Missing_Count), ]

rownames(result) <- NULL

return(result)

}

xunlian <- missing_values_report(df1[c(1:5,65:138)])

write.xlsx(xunlian,"C:\\Users\\wlxy8\\Desktop\\课题\\肺结核鉴别诊断\\研二下诊断分析\\sace全重新做\\不平衡数量\\男训缺失情况.xlsx")

####Median imputation#######

names(dftr_m)

df11 <- df1[c(1:5,68:137)]

df22 <- df2[c(1:5,68:137)]

numeric_cols <- sapply(df11, is.numeric) &

!names(df11) %in% "Species"

train_medians <- df11 %>%

select(where(is.numeric)) %>%

summarise(across(everything(), median, na.rm = TRUE))

cat("训练集各特征中位数:\n")

print(train_medians)

\# Fill function: Fills the data frame using the median vector passed as an argument

fill_median <- function(data, medians) {

for (col in names(medians)) {

if (col %in% names(data)) {

na_idx <- is.na(data[[col]])

data[[col]][na_idx] <- medians[[col]]

}

}

return(data)

}

dftrm <- fill_median(df11, train_medians)

dftem	<- fill_median(df22, train_medians) # Median of the training set

f1<-function(x){

return((x-min(x)) / (max(x)-min(x)))

}

dftr_m$TID <- as.character(dftr_m$TID)

tb<- descrTable(ID~.,data=dftr_m,method=NA)

export2csv(tb,file = "C:\\Users\\wlxy8\\Desktop\\课题\\肺结核鉴别诊断\\研二下诊断分析\\sace全重新做\\不平衡数量\\重新插补后的结果\\男验基线.csv")

train_min <- apply(dftrm[, 6:76], 2, min, na.rm = TRUE)

train_max <- apply(dftrm[, 6:76], 2, max, na.rm = TRUE)

dftr_m1<-as.data.frame(lapply(dftrm[6:76],f1))

dftr_m <- cbind(dftrm[1:5],dftr_m1)

dfte_m1 <- as.data.frame(mapply(function(col, mn, mx) {

(col - mn) / (mx - mn)

}, dftem[, 6:76], train_min, train_max, SIMPLIFY = FALSE))

dfte_m <- cbind(dftem[, 1:5], dfte_m1)

#####Calculate category weights

n_train <- nrow(dftr_m)

n1 <- sum(dftr_m$ID == 1)

n0 <- sum(dftr_m$ID == 0)

w_class0 <- n_train / (2 * n0)

w_class1 <- n_train / (2 * n1)

sample_weights <- ifelse(dftr_m$ID == 1, w_class1, w_class0)

class_weights <- c("0" = w_class0, "1" = w_class1)

getImpRangerWeighted <- function(x, y, ...) {

\# x: 特征矩阵, y: 因子向量

df_temp <- data.frame(x, y = y)

fit <- ranger(

y ~ .,

data = df_temp,

num.trees = 500,

importance = "permutation",

class.weights = class_weights,

probability = FALSE,

...

)

imp <- fit$variable.importance

imp[colnames(x)]

}

######Boruta and lasso select variables######

library(ranger)

dftr_m$ID <- as.factor(dftr_m$ID)

set.seed(123)

boruta_model <- Boruta(ID~.,

data = dftr_m[c("ID","age","HGB","RBC","WBC","BASO9",

"NEU0","Lym0","BASO0","MCV","PLT0","RDWSD","RDWCV","PCT",

"PDW","IgG","IgM","TP","ALB","GLU","SACE","TC",

"TG","HDLC","TGHDLC","LDLHDL","APOE","LPAAPOE","ESR","CD8",

"CD4CD8","FIBs","AT3S","DDimerS","FDPS"

)],

mcAdj = TRUE,

maxRuns = 100,

doTrace = 2,

holdHistory = TRUE,

getImp = getImpRangerWeighted)

Boruta::plotImpHistory(boruta_model)

boruta.variable.imp <- boruta.imp(boruta_model)

head(boruta.variable.imp)

print(boruta_model)

pdf("C:\\Users\\wlxy8\\Desktop\\课题\\肺结核鉴别诊断\\研二下诊断分析\\sace全重新做\\不平衡数量\\重新插补后的结果\\男boruta.pdf",

width = 9, height = 5)

plot(boruta_model,

sort=T,#按重要性排序

whichShadow=c(T,T,T),#是否画出影子特征，(max shadow,mean shadow,min shadow)

las=2,#x轴刻度标签90度旋转，1 or 2

xlab='')#x轴标题

dev.off()

final_model <- TentativeRoughFix(boruta_model)

print(final_model)

selected_features <- getSelectedAttributes(final_model, withTentative = FALSE)

print(selected_features)

#LASSO

\# Perform weighted LASSO using glmnet and select lambda via cross-validation

set.seed(123)

cv_lasso <- cv.glmnet(

x = x,

y = y,

family = "binomial",

alpha = 1, # LASSO

weights = sample_weights,

nfolds = 10,

type.measure = "deviance",

parallel = FALSE

)

y <- as.numeric(as.character(dftr_m$ID))

x <- as.matrix(dftr_m[,c(

"age","Lym0","BASO0","RDWSD","TP","ALB",

"SACE","TGHDLC","ESR","CD8","CD4CD8","DDimerS"

)])

lasso0 <- glmnet(

x,

y,

family="binomial",

alpha=1

)

lambda_seq <- lasso0$lambda

n_lambda <- length(lambda_seq)

set.seed(123)

K <- 10

foldid <- sample(rep(1:K, length.out=nrow(x)))

cv_loss <- matrix(NA,

nrow=K,

ncol=n_lambda)

for(i in 1:K){

train_idx <- foldid!=i

test_idx <- foldid==i

x_train <- x[train_idx,]

y_train <- y[train_idx]

x_test <- x[test_idx,]

y_test <- y[test_idx]

######## 重新计算weights ########

n_train <- length(y_train)

n1 <- sum(y_train==1)

n0 <- sum(y_train==0)

w0 <- n_train/(2*n0)

w1 <- n_train/(2*n1)

weights_train <- ifelse(y_train==1,

w1,

w0)

################################

fit <- glmnet(

x_train,

y_train,

family="binomial",

alpha=1,

lambda=lambda_seq,

weights=weights_train

)

pred <- predict(

fit,

newx=x_test,

type="response"

)

pred <- pmin(

pmax(pred,1e-15),

1-1e-15

)

for(j in 1:n_lambda){

p <- pred[,j]

cv_loss[i,j] <- -2*mean(

y_test*log(p)+

(1-y_test)*log(1-p)

)

}

}

cv_mean <- colMeans(cv_loss)

cv_sd <- apply(cv_loss,2,sd)

cv_se <- cv_sd/sqrt(K)

id_min <- which.min(cv_mean)

lambda_min <- lambda_seq[id_min]

threshold <- cv_mean[id_min]+cv_se[id_min]

id_1se <- max(which(cv_mean<=threshold))

lambda_1se <- lambda_seq[id_1se]

plot(log(lambda_seq),

cv_mean,

pch=16,

type="b",

xlab="log(Lambda)",

ylab="Binomial Deviance",

main="Weighted LASSO (10-fold CV)",

ylim=c(min(cv_mean-cv_se),

max(cv_mean+cv_se)))

segments(log(lambda_seq),

cv_mean-cv_se,

log(lambda_seq),

cv_mean+cv_se)

abline(v=log(lambda_min),

lty=2,

lwd=2,

col="red")

abline(v=log(lambda_1se),

lty=2,

lwd=2,

col="blue")

legend("topright",

c("lambda.min",

"lambda.1se"),

lwd=2,

lty=2,

col=c("red","blue"))

n_total <- length(y)

n1_la <- sum(y==1)

n0_la <- sum(y==0)

w0_la <- n_total/(2*n0)

w1_la <- n_total/(2*n1)

weights_final <- ifelse(y==1,

w1_la,

w0_la)

final_fit <- glmnet(

x,

y,

family="binomial",

alpha=1,

lambda=lambda_seq,

weights=weights_final

)

l.coef2 <- coef(

final_fit,

s=lambda_min

)

l.coef1 <- coef(

final_fit,

s=lambda_1se

)

coef_df <- data.frame(

variable = rownames(l.coef2)[-1],

coefficient =as.vector(l.coef2)[-1]

) %>%

filter(coefficient != 0) %>%

arrange(desc(abs(coefficient)))

\# plot

ggplot(coef_df, aes(x = reorder(variable, abs(coefficient)),

y = coefficient,fill=coefficient>0)) +

geom_bar(stat = "identity") +

coord_flip() +

labs(

x = "variables", y = "coefficient") +

theme_bw()+

theme(panel.grid=element_blank(),legend.position="none")

ggsave("C:\\Users\\wlxy8\\Desktop\\课题\\肺结核鉴别诊断\\研二下诊断分析\\sace全重新做\\不平衡数量\\重新插补后的结果\\男重要性图3.pdf",

height = 6, width = 6

)

features <- coef_df$variable

formula <- as.formula(paste("ID ~", paste(features, collapse = "+")))

modm<-glm(formula ,#family="binomial",

family = quasibinomial(link = "logit"), data = dftr_m,weights = sample_weights)

summary(modm)

vif(modm)

lrprotr<-predict(modm, newdata = dftr_m, type = "response") #这里不需要变动

roc.mod <- reportROC(gold =dftr_m$ID,predictor = lrprotr,important = "se",plot=TRUE)

lrroc1 <- roc(dftr_m$ID,lrprotr)

lrprote<-predict(modm, newdata = dfte_m, type = "response")

lrroc2 <- roc(dfte_m$ID,lrprote)

dca(data=dfte_m,ID~lrprote)

####XGB####

library(xgboost)

library(Matrix)

set.seed(123)

td1 = data.matrix(dftr_m[,features])

\# Convert the independent variables into a sparse matrix

td2 = Matrix(td1,sparse = T)

xgbmtr = list(data = td2, y=as.numeric(as.character(dftr_m$ID)));xgbmtr

\# train

xgbmtr = xgb.DMatrix(data=xgbmtr$data,label = xgbmtr$y,weight = sample_weights)

\# validation

ted1 = data.matrix(dfte_m[,features])

ted2 = Matrix(ted1,sparse = T)

xgbmte = list(data = ted2, y=as.numeric(as.character(dfte_m$ID)))

xgbmte = xgb.DMatrix(data=xgbmte$data,label = xgbmte$y)

set.seed(123)

param_grid <- expand.grid(

max_depth = c(3,5,7),

eta = c(0.01, 0.1, 0.3),

colsample_bytree = c(0.6, 0.8),

min_child_weight = c(3, 5),

subsample = c(0.6, 0.8)

)

b_auc <- 0

b_params <- NULL

for (i in 1:nrow(param_grid)) {

params <- as.list(param_grid[i,])

cv <- xgb.cv(

params = params,

data = xgbmtr,

nrounds = 200,

nfold = 5,

early_stopping_rounds = 10,

objective = "binary:logistic",

metrics = "auc",

verbose = 0

)

if (max(cv$evaluation_log$test_auc_mean) > b_auc) {

b_auc <- max(cv$evaluation_log$test_auc_mean)

b_nrounds <- which.max(cv$evaluation_log$test_auc_mean)

b_params <- params

}

}

xgb.mod <- xgb.train(

params = b_params,

data = xgbmtr,

nrounds = b_nrounds,

objective = "binary:logistic"

)

xgb.pre = predict(xgb.mod,newdata =xgbmtr,type = "prob")

xgb.roc.tr <- roc(dftr_m$ID,xgb.pre)

xgb.prete <- predict(xgb.mod,newdata =xgbmte,type = "prob")

xgb.roc.te <- roc(dfte_m$ID,xgb.prete)

plot(xgb.roc.te)

reportROC(gold =dfte_m$ID,predictor = xgb.prete,important = "se",plot=TRUE)

dca(data = dfte_m, ID ~ xgb.prete)

#########RF##############

library(randomForest)

set.seed(123)

x_train <- dftr_m[, features]

y_train <- as.factor(dftr_m$ID)

n_trainrf <- length(y_train)

n1rf <- sum(y_train == "1")

n0rf <- sum(y_train == "0")

\# Class weights

class_weights <- c(

"0" = n_trainrf / (2 * n0rf),

"1" = n_trainrf / (2 * n1rf)

)

class_weights

########## 1. Tune ntree ##########

ntree_grid <- seq(100, 500, by = 20)

oob <- numeric(length(ntree_grid))

for(i in seq_along(ntree_grid)){

rf <- randomForest(

x = x_train,

y = y_train,

ntree = ntree_grid[i],

mtry = floor(sqrt(ncol(x_train))),

nodesize = 5,

classwt = class_weights

)

oob[i] <- rf$err.rate[nrow(rf$err.rate), "OOB"]

}

\# Select ntree programmatically based on minimum OOB error

optimal_ntree <- ntree_grid[which.min(oob)]

optimal_ntree

\# Plot ntree tuning

plot(

ntree_grid,

oob,

type = "b",

pch = 19,

xlab = "Number of trees",

ylab = "OOB Error"

)

abline(

v = optimal_ntree,

col = "red",

lwd = 2,

lty = 2

)

########## 2. Tune mtry ##########

mtry_grid <- 1:length(features)

oob_error <- numeric(length(mtry_grid))

for(i in seq_along(mtry_grid)){

rf_tmp <- randomForest(

x = x_train,

y = y_train,

mtry = mtry_grid[i],

ntree = optimal_ntree,

nodesize = 5,

classwt = class_weights,

importance = FALSE

)

oob_error[i] <- rf_tmp$err.rate[

nrow(rf_tmp$err.rate),

"OOB"

]

}

results <- data.frame(

mtry = mtry_grid,

OOB = oob_error

)

results

\# Select mtry programmatically

optimal_mtry <- results$mtry[

which.min(results$OOB)

]

optimal_mtry

\# Plot mtry tuning

plot(

results$mtry,

results$OOB,

type = "b",

pch = 19,

xlab = "mtry",

ylab = "OOB Error",

main = "Random Forest Hyperparameter Tuning"

)

abline(

v = optimal_mtry,

col = "red",

lwd = 2,

lty = 2

)

########## 3. Final Random Forest model ##########

set.seed(123)

m_rf_model <- randomForest(

x = x_train,

y = y_train,

ntree = optimal_ntree,

nodesize = 5,

mtry = optimal_mtry,

classwt = class_weights,

importance = TRUE

)

print(m_rf_model)

plot(

m_rf_model,

lwd = 2,

main = "OOB MSE"

)

########## 4. Training set prediction ##########

m_rftrpre <- predict(m_rf_model,newdata = dftr_m)

m_rftrpro <- predict(m_rf_model,newdata = dftr_m,type = "prob")[, 2]

m_rf_roctr <- roc(as.numeric(as.character(dftr_m$ID)),m_rftrpro)

########## 5. Validation set prediction ##########

m_rftepro <- predict(m_rf_model,newdata = dfte_m,type = "prob")[, 2]

m_rfprete <- predict(m_rf_model,newdata = dfte_m)

m_rf_rocte <- roc(as.numeric(as.character(dfte_m$ID)),m_rftepro)

###SVM#####

library(caret)

library(e1071)

library(kernlab)

msvmx <- dftr_m[,features]

msvmy <- factor(dftr_m$ID)

set.seed(123)

tuned_model <- tune.svm(formula,

data = dftr_m,class.weights = class_weights,

cost = 10^(-1:3),gamma = 10^(-3:1),

tunecontrol=tune.control(sampling = "bootstrap",

nboot = 100))

print(tuned_model)

best_cost_svm <- tuned_model$best.parameters$cost

best_gamma_svm <- tuned_model$best.parameters$gamma

set.seed(123)

m_svmmod <- svm(formula,

data = dftr_m,class.weights=class_weights,

probability = T,

kernel="radial",type = "C-classification",

gamma=best_gamma_svm,

cost=best_cost_svm)

print(m_svmmod)

summary(m_svmmod)

msvmpre <- predict(m_svmmod,newdata = dftr_m,probability = T)

msvmprotr <- attr(msvmpre,"probabilities")[,2]

m_svmroctr <- roc(as.numeric(dftr_m$ID),msvmprotr)

msvmprete <- predict(m_svmmod,newdata = dfte_m,probability = T)

m_svmrocte <- roc(as.numeric(dfte_m$ID),attr(msvmprete,"probabilities")[,2])

msvmprote <- attr(msvmprete,"probabilities")[,2]

######SVM 10-folds Cross-validation#########

X <- dftr_m[, features]

Y <- factor(dftr_m$ID)

levels(Y) <- make.names(levels(Y))	# 转换为合法因子水平 "X0", "X1"

positive_class <- levels(Y)[2]	# 正类（少数类，通常为 "X1"）

\# 外层 10 折划分

set.seed(123)

outer_folds <- createFolds(Y, k = 10, returnTrain = FALSE)

\# 存储结果

cv_results <- data.frame(

Fold = 1:10,

Cutoff = NA,

AUC = NA,

Accuracy = NA,

Sensitivity = NA,

Specificity = NA,

Best_cost = NA,

Best_gamma = NA

)

all_predictions <- data.frame()

\# ========== 外层 10 折循环 ==========

for (i in 1:10) {

cat("Outer fold:", i, "\n")

test_idx <- outer_folds[[i]]

train_idx <- setdiff(1:length(Y), test_idx)

X_train <- X[train_idx, ]

Y_train <- Y[train_idx]

X_test <- X[test_idx, ]

Y_test <- Y[test_idx]

\# ---- 动态权重（局部命名，不覆盖全局变量） ----

n_fold <- length(Y_train)

n1_fold <- sum(Y_train == positive_class)

n0_fold <- sum(Y_train == levels(Y)[1])

w0_fold <- n_fold / (2 * n0_fold)

w1_fold <- n_fold / (2 * n1_fold)

class_weights_fold <- setNames(c(w0_fold, w1_fold), levels(Y_train))

\# ===== 步骤 2：内层调参（bootstrap 100 次） =====

set.seed(456 + i)	# 每折可设不同种子，避免全部相同

inner_tune <- tune.svm(

x = X_train,

y = Y_train,

class.weights = class_weights_fold,

cost = 10^(-1:3), # 0.1, 1, 10, 100, 1000

gamma = 10^(-3:1), # 0.001, 0.01, 0.1, 1, 10

tunecontrol = tune.control(sampling = "bootstrap", nboot = 100)

)

best_cost <- inner_tune$best.parameters$cost

best_gamma <- inner_tune$best.parameters$gamma

cv_results$Best_cost[i] <- best_cost

cv_results$Best_gamma[i] <- best_gamma

\# ===== 步骤 3：用最佳参数训练 SVM =====

svm_fold <- svm(

x = X_train,

y = Y_train,

class.weights = class_weights_fold,

probability = TRUE,

kernel = "radial",

type = "C-classification",

gamma = best_gamma,

cost = best_cost

)

\# ===== 步骤 4：在训练部分上确定最佳阈值（约登指数） =====

train_prob <- predict(svm_fold, newdata = X_train, probability = TRUE)

train_prob_values <- attr(train_prob, "probabilities")[, positive_class]

\# 安全检查：训练集必须包含两类

if (length(unique(Y_train)) < 2) {

stop("Fold ", i, " 训练集只有一个类别，请检查数据")

}

train_roc <- roc(response = Y_train, predictor = train_prob_values, quiet = TRUE)

best_cutoff <- as.numeric(

coords(train_roc, x = "best", best.method = "youden", ret = "threshold")

)

cv_results$Cutoff[i] <- best_cutoff

\# ===== 步骤 5：在测试部分上预测与评估 =====

test_prob <- predict(svm_fold, newdata = X_test, probability = TRUE)

test_prob_values <- attr(test_prob, "probabilities")[, positive_class]

test_class <- ifelse(test_prob_values >= best_cutoff,

positive_class,

levels(Y)[1])

test_class <- factor(test_class, levels = levels(Y))

\# 混淆矩阵与指标

cm <- confusionMatrix(test_class, Y_test, positive = positive_class)

cv_results$Accuracy[i]	<- cm$overall["Accuracy"]

cv_results$Sensitivity[i] <- cm$byClass["Sensitivity"]

cv_results$Specificity[i] <- cm$byClass["Specificity"]

test_roc <- roc(response = Y_test, predictor = test_prob_values, quiet = TRUE)

cv_results$AUC[i] <- auc(test_roc)

\# 保存该折的详细预测

fold_pred <- data.frame(

Fold = i,

ID = Y_test,

Probability = test_prob_values,

Predicted = test_class,

Cutoff = best_cutoff

)

all_predictions <- rbind(all_predictions, fold_pred)

}

\# ========== 结果汇总 ==========

print(cv_results)

cat("\n====================\n")

cat("Nested 10-fold CV results (weights & tuning inside each fold)\n")

cat("====================\n")

cat("AUC: ", round(mean(cv_results$AUC), 3), " +/- ", round(sd(cv_results$AUC), 3), "\n")

cat("Accuracy: ", round(mean(cv_results$Accuracy), 3), " +/- ", round(sd(cv_results$Accuracy), 3), "\n")

cat("Sensitivity: ", round(mean(cv_results$Sensitivity), 3), " +/- ", round(sd(cv_results$Sensitivity), 3), "\n")

cat("Specificity: ", round(mean(cv_results$Specificity), 3), " +/- ", round(sd(cv_results$Specificity), 3), "\n")

cat("Cutoff: ", round(mean(cv_results$Cutoff), 3), " +/- ", round(sd(cv_results$Cutoff), 3), "\n")

cv_long <- cv_results %>%

select(Fold, Accuracy, AUC, Sensitivity, Specificity) %>%

pivot_longer(cols = c(Accuracy, AUC, Sensitivity, Specificity),

names_to = "Metric", values_to = "Value")

\# 设置指标顺序，让图形排列整齐

cv_long$Metric <- factor(cv_long$Metric,

levels = c("AUC", "Accuracy", "Sensitivity", "Specificity"))

\# 绘图：箱式图 + 散点叠加

ggplot(cv_long, aes(x = Metric, y = Value)) +

geom_jitter(width = 0.2, alpha = 0.5) +

geom_boxplot(width = 0.3,fill = "white", outlier.shape = NA) +

stat_summary(fun = mean, geom = "point",

shape = 18, size = 2, color = "darkred") +

\# scale_fill_manual(values = c("#fc9f01", "#a1a0a5", "#004081")) +

\# scale_y_continuous(limits = c(0.7,1),breaks = seq(0.7,1.0,0.1))+

labs(title = "",

x = "", y = "") +

theme_classic(base_size = 10) +

theme(legend.position = "none",

plot.title = element_text(hjust = 0.5, face = "bold"),

axis.text.x = element_text(angle = 45, hjust = 1))

ggsave("C:\\Users\\wlxy8\\Desktop\\课题\\肺结核鉴别诊断\\研二下诊断分析\\sace全重新做\\不平衡数量\\重新插补后的结果\\男SVM交叉验证.pdf",width=4,height=3,dpi=300)

####KNN#####

library(kknn)

library(caret)

library(pROC)

set.seed(123)

knn_model <- kknn(

formula = ID~Lym0+RDWSD+ESR+TGHDLC+BASO0+TP+CD4CD8+age+SACE+DDimerS, # 预测y

train	= dftr_m, # train data

test	= dfte_m, # validation data

k	= 5,

distance = 2,

kernel = "triangular"

)

summary(knn_model)

pred_prob <- knn_model$prob[, "1"]

\# AUC

auc_val <- roc(dfte_m$ID, pred_prob)$auc

cat("验证集 AUC:", round(auc_val, 4), "\n")

k_values <- seq(3, 51, by = 2) # 奇数

cv_results <- data.frame(k = k_values, auc = NA)

#Manual 5-fold cross-validation

set.seed(123)

folds_knn <-createFolds(1:nrow(dftr_m), k = 5, list = TRUE)

for (j in seq_along(k_values)) {

k_val <- k_values[j]

fold_auc <- numeric(5)

for (i in 1:5) {

val_idx <- folds_knn[[i]]

tr_idx <- setdiff(1:nrow(dftr_m), val_idx)

tr_fold <- dftr_m[tr_idx, ]

val_fold <- dftr_m[val_idx, ]

\# 使用kknn

fit_fold <- kknn(

ID~Lym0+RDWSD+ESR+TGHDLC+BASO0+TP+CD4CD8+age+SACE+DDimerS,

train = tr_fold,

test = val_fold,

k = k_val,

distance = 2,

kernel = "triangular"

)

prob_fold <- fit_fold$prob[, "1"]

fold_auc[i] <- roc(val_fold$ID, prob_fold)$auc

}

cv_results$auc[j] <- mean(fold_auc)

cat("k =", k_val, "平均AUC =", round(mean(fold_auc), 4), "\n")

}

best_k <- cv_results$k[which.max(cv_results$auc)]

cat("\n最佳K值:", best_k, "\n")

final_knn <- kknn(

ID~Lym0+RDWSD+ESR+TGHDLC+BASO0+TP+CD4CD8+age+SACE+DDimerS,

train = dftr_m,

test = dfte_m,

k = best_k,

distance = 2,

kernel = "triangular"

)

#train

set.seed(123)

folds_knn <- createFolds(1:nrow(dftr_m), k = 5, list = TRUE)

\# 存储每个样本的交叉验证预测概率

cv_probs <- numeric(nrow(dftr_m))

for (i in 1:5) {

val_idx <- folds_knn[[i]]

tr_idx <- setdiff(1:nrow(dftr_m), val_idx)

tr_fold <- dftr_m[tr_idx, ]

val_fold <- dftr_m[val_idx, ]

fit_cv <- kknn(

ID ~ Lym0 + RDWSD + ESR + TGHDLC + BASO0 + TP + CD4CD8 + age + SACE + DDimerS,

train = tr_fold,

test = val_fold,

k = best_k,

distance = 2,

kernel = "triangular"

)

cv_probs[val_idx] <- fit_cv$prob[, "1"]

}

\# 计算交叉验证 AUC（无偏的训练集性能估计）

cv_auc_train <- roc(dftr_m$ID, cv_probs)$auc

cat("训练集交叉验证 AUC (无偏):", round(cv_auc_train, 4), "\n")

knntr_cv <- roc(dftr_m$ID, cv_probs, quiet = TRUE)

\# 提取新 AUC 及其 95% CI

auc_cv <- auc(knntr_cv)

auc_ci_cv <- ci.auc(knntr_cv, method = "delong")

knntepro <- final_knn$prob[, "1"]

knnte <- roc(dfte_m$ID, knntepro)

reportROC(dftr_m$ID, cv_probs)

########NB##########

library(e1071)

library(pROC)

library(caret)

\# Normalize the category weights to prior probabilities

prior_prob <- class_weights / sum(class_weights)

names(prior_prob) <- levels(dftr_m$ID)

cat("加权先验概率:", prior_prob, "\n")

nb_model <- naiveBayes(

x = dftr_m[, features],

y = dftr_m$ID,

prior = prior_prob,

laplace = 1

)

nbtrpro <- predict(nb_model, dftr_m[, features], type = "raw")[, "1"]

nbtr <- roc(dftr_m$ID, nbtrpro)

nbtepro <- predict(nb_model, dfte_m[, features], type = "raw")[, "1"]

nbte <- roc(dfte_m$ID, nbtepro)

m_roc_list <- list(

LR = lrroc1,

XGBoost = xgb.roc.tr,

RF = m_rf_roctr,

KNN = knntr_cv,

SVM = m_svmroctr,

`Naive Bayes` = nbtr

)

\# 定义模型颜色

m_model_colors <- c(

LR = "#fed439",

XGBoost = "#709ae1",

RF = "#8a9197",

KNN = "#d2af81",

SVM = "#fd7446",

`Naive Bayes` = "#d5e4a2"

)

\# 动态提取AUC及95% CI

m_roc_labels <- do.call(rbind, lapply(names(m_roc_list), function(model) {

roc_obj <- m_roc_list[[model]]

auc_value <- as.numeric(auc(roc_obj))

ci_value <- as.numeric(ci.auc(roc_obj))

data.frame(

Model = model,

AUC = auc_value,

CI_lower = ci_value[1],

CI_upper = ci_value[3]

)

}))

\# 生成动态标签

m_roc_labels$label <- paste0(

m_roc_labels$Model,

": AUC = ",

sprintf("%.3f", m_roc_labels$AUC),

" (",

sprintf("%.3f", m_roc_labels$CI_lower),

" to ",

sprintf("%.3f", m_roc_labels$CI_upper),

")"

)

\# 设置文字位置

m_roc_labels$x <- 0.5

m_roc_labels$y <- c(0.25, 0.20, 0.15, 0.10, 0.05, 0.00)

\# 绘制ROC曲线

m_trainroc <- ggroc(

m_roc_list,

legacy.axes = TRUE,

size = 1

) +

geom_segment(

aes(x = 0, xend = 1, y = 0, yend = 1),

color = "darkgrey",

linetype = "dashed"

) +

\# ROC曲线颜色

scale_colour_manual(

values = m_model_colors

) +

\# 动态添加AUC和95% CI

geom_text(

data = m_roc_labels,

aes(

x = x,

y = y,

label = label,

color = Model

),

inherit.aes = FALSE,

size = 4,

family = "sans",

hjust = 0,

vjust = 0

) +

theme_bw() +

theme(

panel.grid = element_blank(),

text = element_text(family = "sans"),

legend.position = "none",

plot.title = element_text(hjust = 0.5)

)

ggsave("C:\\Users\\wlxy8\\Desktop\\课题\\肺结核鉴别诊断\\研二下诊断分析\\sace全重新做\\不平衡数量\\重新插补后的结果\\男ROC训练集.pdf",width=7,height=7,dpi=300)

m_roc_list2 <- list(

LR = lrroc2,

XGBoost = xgb.roc.te,

RF = m_rf_rocte,

KNN = knnte,

SVM = m_svmrocte,

`Naive Bayes` = nbte

)

\# 模型颜色

m_model_colors <- c(

LR = "#fed439",

XGBoost = "#709ae1",

RF = "#8a9197",

KNN = "#d2af81",

SVM = "#fd7446",

`Naive Bayes` = "#d5e4a2"

)

\# 动态提取AUC和95% CI

m_roc_labels2 <- do.call(

rbind,

lapply(names(m_roc_list2), function(model) {

roc_obj <- m_roc_list2[[model]]

auc_value <- as.numeric(auc(roc_obj))

\# AUC=1时直接设定CI为1-1，避免ci.auc() warning

if (auc_value >= 0.999999) {

ci_lower <- 1

ci_upper <- 1

} else {

ci_value <- as.numeric(ci.auc(roc_obj))

ci_lower <- ci_value[1]

ci_upper <- ci_value[3]

}

data.frame(

Model = model,

AUC = auc_value,

CI_lower = ci_lower,

CI_upper = ci_upper

)

})

)

\# 动态生成标签

m_roc_labels2$label <- paste0(

m_roc_labels2$Model,

": AUC = ",

sprintf("%.3f", m_roc_labels2$AUC),

" (",

sprintf("%.3f", m_roc_labels2$CI_lower),

" to ",

sprintf("%.3f", m_roc_labels2$CI_upper),

")"

)

\# 设置文字位置

m_roc_labels2$x <- 0.5

m_roc_labels2$y <- c(

0.25,

0.20,

0.15,

0.10,

0.05,

0.00

)

\# 绘制ROC曲线

m_testroc <- ggroc(

m_roc_list2,

legacy.axes = TRUE,

size = 1

) +

\# 对角线

geom_segment(

aes(

x = 0,

xend = 1,

y = 0,

yend = 1

),

color = "darkgrey",

linetype = "dashed"

) +

\# ROC曲线颜色

scale_colour_manual(

values = m_model_colors

) +

\# 动态添加AUC和95% CI

geom_text(

data = m_roc_labels2,

aes(

x = x,

y = y,

label = label,

color = Model

),

inherit.aes = FALSE,

size = 4,

family = "sans",

hjust = 0,

vjust = 0

) +

theme_bw() +

theme(

panel.grid = element_blank(),

text = element_text(family = "sans"),

legend.position = "none",

plot.title = element_text(hjust = 0.5)

)

ggsave("C:\\Users\\wlxy8\\Desktop\\课题\\肺结核鉴别诊断\\研二下诊断分析\\sace全重新做\\不平衡数量\\重新插补后的结果\\男ROC验证集.pdf",width=7,height=7,dpi=300)

#######Calculate the discrimination index#########

calc_metrics_at_cutoff <- function(gold, prob, cutoff) {

pred <- ifelse(prob >= cutoff, 1, 0)

\# Construct a confusion matrix

tp <- sum(pred == 1 & gold == 1)

tn <- sum(pred == 0 & gold == 0)

fp <- sum(pred == 1 & gold == 0)

fn <- sum(pred == 0 & gold == 1)

n <- length(gold)

\# (Sensitivity) = TP / (TP+FN)

sen <- tp / (tp + fn)

\# (Specificity) = TN / (TN+FP)

spe <- tn / (tn + fp)

\# (Accuracy) = (TP+TN)/n

acc <- (tp + tn) / n

\# (PPV) = TP / (TP+FP)

ppv <- tp / (tp + fp)

\# (NPV) = TN / (TN+FN)

npv <- tn / (tn + fn)

wilson_ci <- function(x, n) {

if (n == 0) return(c(NA, NA))

p <- x / n

z <- qnorm(0.975)

denom <- 1 + z^2/n

centre <- p + z^2/(2*n)

half <- z * sqrt((p*(1-p) + z^2/(4*n)) / n)

c((centre - half)/denom, (centre + half)/denom)

}

sen_ci <- wilson_ci(tp, tp+fn)

spe_ci <- wilson_ci(tn, tn+fp)

acc_ci <- wilson_ci(tp+tn, n)

ppv_ci <- wilson_ci(tp, tp+fp)

npv_ci <- wilson_ci(tn, tn+fn)

return(data.frame(

Cutoff = cutoff,

Sensitivity = sen, Sensitivity_low = sen_ci[1], Sensitivity_up = sen_ci[2],

Specificity = spe, Specificity_low = spe_ci[1], Specificity_up = spe_ci[2],

Accuracy = acc, Accuracy_low = acc_ci[1], Accuracy_up = acc_ci[2],

PPV = ppv, PPV_low = ppv_ci[1], PPV_up = ppv_ci[2],

NPV = npv, NPV_low = npv_ci[1], NPV_up = npv_ci[2]

))

}

###train set

MLmodels <- list(

LR = lrprotr, RF = m_rftrpro, XGB = xgb.pre,

KNN = cv_probs, SVM = msvmprotr, NB = nbtrpro

)

results_list <- list()

cutoff_list <- list()

for (name in names(MLmodels)) {

res <- reportROC(gold = dftr_m$ID, predictor = MLmodels[[name]],

important = "se", plot = FALSE)

\# Extract the optimal cutoff calculated on the training set (for use on the validation set)

cutoff_train <- as.numeric(res$Cutoff)

cutoff_list[[name]] <- cutoff_train

results_list[[name]] <- data.frame(

Model = name,

Cutoff = round(cutoff_train, 3),

AUC = round(as.numeric(res$AUC), 3),

AUC_low = round(as.numeric(res$AUC.low), 3),

AUC_up = round(as.numeric(res$AUC.up), 3),

Sensitivity = round(as.numeric(res$SEN), 3),

Sensitivity_low = round(as.numeric(res$SEN.low), 3),

Sensitivity_up = round(as.numeric(res$SEN.up), 3),

Specificity = round(as.numeric(res$SPE), 3),

Specificity_low = round(as.numeric(res$SPE.low), 3),

Specificity_up = round(as.numeric(res$SPE.up), 3),

Accuracy = round(as.numeric(res$ACC), 3),

Accuracy_low = round(as.numeric(res$ACC.low), 3),

Accuracy_up = round(as.numeric(res$ACC.up), 3),

PPV = round(as.numeric(res$PPV), 3),

PPV_low = round(as.numeric(res$PPV.low), 3),

PPV_up = round(as.numeric(res$PPV.up), 3),

NPV = round(as.numeric(res$NPV), 3),

NPV_low = round(as.numeric(res$NPV.low), 3),

NPV_up = round(as.numeric(res$NPV.up), 3)

)

}

final_res_tr <- do.call(rbind, results_list)

write.xlsx(final_res_tr, "C:\\Users\\wlxy8\\Desktop\\课题\\肺结核鉴别诊断\\研二下诊断分析\\sace全重新做\\不平衡数量\\重新插补后的结果\\男训区分度5.xlsx")

###validation set

MLmodels2 <- list(

LR = lrprote, RF = m_rftepro, XGB = xgb.prete,

KNN = knntepro, SVM = msvmprote, NB = nbtepro

)

results_list2 <- list()

for (name in names(MLmodels2)) {

roc_obj <- roc(dfte_m$ID, MLmodels2[[name]], quiet = TRUE)

auc_val <- auc(roc_obj)

auc_ci <- ci.auc(roc_obj, method = "delong")

\# 2. 使用训练集上得到的 cutoff 计算验证集的二分类指标

cutoff_train <- cutoff_list[[name]] # 从训练集传入

metrics_val <- calc_metrics_at_cutoff(gold = dfte_m$ID,

prob = MLmodels2[[name]],

cutoff = cutoff_train)

results_list2[[name]] <- data.frame(

Model = name,

Cutoff_train = round(cutoff_train, 3),

AUC = round(auc_val, 3),

AUC_low = round(auc_ci[1], 3),

AUC_up = round(auc_ci[3], 3),

Sensitivity = round(metrics_val$Sensitivity, 3),

Sensitivity_low = round(metrics_val$Sensitivity_low, 3),

Sensitivity_up = round(metrics_val$Sensitivity_up, 3),

Specificity = round(metrics_val$Specificity, 3),

Specificity_low = round(metrics_val$Specificity_low, 3),

Specificity_up = round(metrics_val$Specificity_up, 3),

Accuracy = round(metrics_val$Accuracy, 3),

Accuracy_low = round(metrics_val$Accuracy_low, 3),

Accuracy_up = round(metrics_val$Accuracy_up, 3),

PPV = round(metrics_val$PPV, 3),

PPV_low = round(metrics_val$PPV_low, 3),

PPV_up = round(metrics_val$PPV_up, 3),

NPV = round(metrics_val$NPV, 3),

NPV_low = round(metrics_val$NPV_low, 3),

NPV_up = round(metrics_val$NPV_up, 3)

)

}

final_res_te <- do.call(rbind, results_list2)

write.xlsx(final_res_te, "C:\\Users\\wlxy8\\Desktop\\课题\\肺结核鉴别诊断\\研二下诊断分析\\sace全重新做\\不平衡数量\\重新插补后的结果\\男验区分度5.xlsx")

#######MALE/Differential Diagnosis of Sarcoidosis from Specific Control Diseases######

cancer <- dftem%>%

filter(TID==1|

TID==3)

names(cancer)

cancer1 <- as.data.frame(mapply(function(col, mn, mx) {

(col - mn) / (mx - mn)

}, cancer[, 6:76], train_min, train_max, SIMPLIFY = FALSE))

cancer <- cbind(cancer[, 1:5], cancer1)

can_svmpre <- predict(m_svmmod,newdata = cancer,probability = T)

can_roc <- roc(as.numeric(cancer$ID),attr(can_svmpre,"probabilities")[,2])

canpro <- attr(can_svmpre,"probabilities")[,2]

b_co_svm <- coords(m_svmroctr, "best", best.method = "youden")#计算最佳截断点

b_co_svm

pc_can <- ifelse(canpro>= b_co_svm$threshold, 1, 0)

can_cm <- confusionMatrix( as.factor(pc_can),as.factor(cancer$ID),

positive = "1", mode = "everything")

tub <- dftem%>%

filter(TID==1|

TID==0)

tub1 <- as.data.frame(mapply(function(col, mn, mx) {

(col - mn) / (mx - mn)

}, tub[, 6:76], train_min, train_max, SIMPLIFY = FALSE))

tub <- cbind(tub[, 1:5], tub1)

tub_svmpre <- predict(m_svmmod,newdata = tub,probability = T)

tub_roc <- roc(as.numeric(tub$ID),attr(tub_svmpre,"probabilities")[,2])

tubpro <- attr(tub_svmpre,"probabilities")[,2]

pc_tub <- ifelse(tubpro>= b_co_svm$threshold, 1, 0)

tub_cm <- confusionMatrix( as.factor(pc_tub),as.factor(tub$ID),

positive = "1", mode = "everything")

pneu <- dftem%>%

filter(TID==1|

TID==4)

pneu1 <- as.data.frame(mapply(function(col, mn, mx) {

(col - mn) / (mx - mn)

}, pneu[, 6:76], train_min, train_max, SIMPLIFY = FALSE))

pneu <- cbind(pneu[, 1:5], pneu1)

pneu_svmpre <- predict(m_svmmod,newdata = pneu,probability = T)

pneu_roc <- roc(as.numeric(pneu$ID),attr(pneu_svmpre,"probabilities")[,2])

pneupro <- attr(pneu_svmpre,"probabilities")[,2]

pc_pneu <- ifelse(pneupro>= b_co_svm$threshold, 1, 0)

pneu_cm <- confusionMatrix( as.factor(pc_pneu),as.factor(pneu$ID),

positive = "1", mode = "everything")

reportROC(cancer$ID,canpro,plot = T)

\# lab_ID <- list(

cancer = cancer$ID,

tub	= tub$ID,

pneu	= pneu$ID,

lym	= lym$ID

)

res_cancer <- calc_metrics_at_cutoff(

gold	= as.numeric(as.character(cancer$ID)),

prob	= as.numeric(canpro),

cutoff = b_co_svm$threshold

)

res_cancer$Dataset <- "cancer"

res_tub <- calc_metrics_at_cutoff(

gold	= as.numeric(as.character(tub$ID)),

prob	= tubpro,

cutoff = b_co_svm$threshold

)

res_tub$Dataset <- "tub"

res_pneu <- calc_metrics_at_cutoff(

gold	= pneu$ID,

prob	= pneupro,

cutoff = b_co_svm$threshold

)

res_pneu$Dataset <- "pneu"

\# 合并结果

obo_res <- rbind(res_cancer, res_tub, res_pneu)

write.csv(obo_res,"C:\\Users\\wlxy8\\Desktop\\课题\\肺结核鉴别诊断\\研二下诊断分析\\sace全重新做\\不平衡数量\\重新插补后的结果\\一对一检验结果\\男一对一1.csv")

##########

######index

best_cutoff <- coords(lrroc1, "best", best.method = "youden")#计算最佳截断点

best_cutoff

pred_class <- ifelse(lrprotr >= best_cutoff$threshold, 1, 0)

conf_matrix <- table(Actual = dftr_m$ID, Pred = pred_class)

mlrcmtr <- confusionMatrix( as.factor(pred_class),as.factor(dftr_m$ID),

positive = "1", mode = "everything")

pred_class2 <- ifelse(lrprote >= best_cutoff$threshold, 1, 0)

conf_matrix2 <- table(Actual = dfte_m$ID, Pred = pred_class2)

mlrcmte <- confusionMatrix( as.factor(pred_class2),as.factor(dfte_m$ID),

positive = "1", mode = "everything")

best_cutoff <- coords(xgb.roc.tr, "best", best.method = "youden")#计算最佳截断点

best_cutoff

pred_class <- ifelse(xgb.pre >= best_cutoff$threshold, 1, 0)

conf_matrix <- table(Actual = dftr_m$ID, Pred = pred_class)

mxgbcmtr <- confusionMatrix(as.factor(pred_class),as.factor(dftr_m$ID),

positive = "1", mode = "everything")

pred_class2 <- ifelse(xgb.prete >= best_cutoff$threshold, 1, 0)

conf_matrix2 <- table(Actual = dfte_m$ID, Pred = pred_class2)

mxgbcmte <- confusionMatrix(as.factor(pred_class2),as.factor(dfte_m$ID),

positive = "1", mode = "everything")

best_cutoff <- coords(m_rf_roctr, "best", best.method = "youden")#计算最佳截断点

best_cutoff

pred_class <- ifelse(m_rftrpro >= best_cutoff$threshold, 1, 0)

conf_matrix <- table(Actual = dftr_m$ID, Pred = pred_class)

mrfcmtr <- confusionMatrix( as.factor(pred_class),as.factor(dftr_m$ID),

positive = "1", mode = "everything")

pred_class2 <- ifelse(m_rftepro >= best_cutoff$threshold, 1, 0)

conf_matrix2 <- table(Actual = dfte_m$ID, Pred = pred_class2)

mrfcmte <- confusionMatrix( as.factor(pred_class2),as.factor(dfte_m$ID),

positive = "1", mode = "everything")

best_cutoff <- coords(m_svmroctr, "best", best.method = "youden")#计算最佳截断点

best_cutoff

pred_class <- ifelse(msvmprotr >= best_cutoff$threshold, 1, 0)

conf_matrix <- table(Actual = dftr_m$ID, Pred = pred_class)

msvmcmtr <- confusionMatrix( as.factor(pred_class),as.factor(dftr_m$ID),

positive = "1", mode = "everything")

pred_class2 <- ifelse(msvmprote >= best_cutoff$threshold, 1, 0)

conf_matrix2 <- table(Actual = dfte_m$ID, Pred = pred_class2)

msvmcmte <- confusionMatrix( as.factor(pred_class2),as.factor(dfte_m$ID),

positive = "1", mode = "everything")

best_cutoff <- coords(knntr_cv, "best", best.method = "youden")#计算最佳截断点

best_cutoff

pred_class <- ifelse(cv_probs>= best_cutoff$threshold, 1, 0)

conf_matrix <- table(Actual = dftr_m$ID, Pred = pred_class)

mknncmtr <- confusionMatrix( as.factor(pred_class),as.factor(dftr_m$ID),

positive = "1", mode = "everything")

pred_class2 <- ifelse(knntepro >= best_cutoff$threshold, 1, 0)

conf_matrix2 <- table(Actual = dfte_m$ID, Pred = pred_class2)

mknncmte <- confusionMatrix( as.factor(pred_class2),as.factor(dfte_m$ID),

positive = "1", mode = "everything")

best_cutoff <- coords(nbtr, "best", best.method = "youden")#计算最佳截断点

best_cutoff

pred_class <- ifelse(nbtrpro >= best_cutoff$threshold, 1, 0)

conf_matrix <- table(Actual = dftr_m$ID, Pred = pred_class)

mnbcmtr <- confusionMatrix( as.factor(pred_class),as.factor(dftr_m$ID),

positive = "1", mode = "everything")

pred_class2 <- ifelse(nbtepro >=best_cutoff$threshold, 1, 0)

conf_matrix2 <- table(Actual = dfte_m$ID, Pred = pred_class2)

mnbcmte <- confusionMatrix( as.factor(pred_class2),as.factor(dfte_m$ID),

positive = "1", mode = "everything")

mcm_result <- data.frame(lr <- c(

round(mlrcmtr$byClass["Sensitivity"], 3),

round(mlrcmtr$byClass["Specificity"], 3),

round(mlrcmtr$byClass["F1"], 3)),

xgb <- c(

round(mxgbcmtr$byClass["Sensitivity"], 3),

round(mxgbcmtr$byClass["Specificity"], 3),

round(mxgbcmtr$byClass["F1"], 3)),

rf <- c(

round(mrfcmtr$byClass["Sensitivity"], 3),

round(mrfcmtr$byClass["Specificity"], 3),

round(mrfcmtr$byClass["F1"], 3)),

svm <- c(

round(msvmcmtr$byClass["Sensitivity"], 3),

round(msvmcmtr$byClass["Specificity"], 3),

round(msvmcmtr$byClass["F1"], 3)),

knn <- c(

round(mknncmtr$byClass["Sensitivity"], 3),

round(mknncmtr$byClass["Specificity"], 3),

round(mknncmtr$byClass["F1"], 3)),

nb <- c(

round(mnbcmtr$byClass["Sensitivity"], 3),

round(mnbcmtr$byClass["Specificity"], 3),

round(mnbcmtr$byClass["F1"], 3))

)

write.csv(mcm_result,"C:\\Users\\wlxy8\\Desktop\\课题\\肺结核鉴别诊断\\研二下诊断分析\\sace全重新做\\不平衡数量\\重新插补后的结果\\男训练集各指标结果3.csv")

mcmte_result <- data.frame(lr <- c(

round(mlrcmte$byClass["Sensitivity"], 3),

round(mlrcmte$byClass["Specificity"], 3),

round(mlrcmte$byClass["F1"], 3)),

xgb <- c(

round(mxgbcmte$byClass["Sensitivity"], 3),

round(mxgbcmte$byClass["Specificity"], 3),

round(mxgbcmte$byClass["F1"], 3)),

rf <- c(

round(mrfcmte$byClass["Sensitivity"], 3),

round(mrfcmte$byClass["Specificity"], 3),

round(mrfcmte$byClass["F1"], 3)),

svm <- c(

round(msvmcmte$byClass["Sensitivity"], 3),

round(msvmcmte$byClass["Specificity"], 3),

round(msvmcmte$byClass["F1"], 3)),

knn <- c(

round(mknncmte$byClass["Sensitivity"], 3),

round(mknncmte$byClass["Specificity"], 3),

round(mknncmte$byClass["F1"], 3)),

nb <- c(

round(mnbcmte$byClass["Sensitivity"], 3),

round(mnbcmte$byClass["Specificity"], 3),

round(mnbcmte$byClass["F1"], 3))

)

write.csv(mcmte_result,"C:\\Users\\wlxy8\\Desktop\\课题\\肺结核鉴别诊断\\研二下诊断分析\\sace全重新做\\不平衡数量\\重新插补后的结果\\男验证集各指标结果3.csv")

##########DCA##########

dca_data <- data.frame(

outcome = as.numeric(as.character(dftr_m$ID)),

LR = lrprotr,

XGBoost=xgb.pre,

RF = m_rftrpro,

KNN=cv_probs,

SVM = msvmprotr,

NaiveBayes=nbtrpro

)

dca_results <- dca(

data = dca_data,

outcome~.,

thresholds = seq(0.01, 0.99, 0.01)

)

plot(dca_results,lwd=5) +

theme_bw() +

labs(title = "Decision Curve Analysis (Male model)",

x = "Threshold probability",

y = "Net Benefit") +

scale_color_manual(values = c("darkgrey","grey","#fed439","#709ae1","#8a9197","#d2af81","#fd7446","#d5e4a2") )+

theme(legend.position = c(0.9,0.8),

panel.grid=element_blank(),

legend.key.size = unit(13, "pt"))

ggsave("C:\\Users\\wlxy8\\Desktop\\课题\\肺结核鉴别诊断\\研二下诊断分析\\sace全重新做\\不平衡数量\\重新插补后的结果\\男DCA训练集4.pdf",width=7,height=7,dpi=300)

#validation DCA

dca_datate <- data.frame(

outcome = as.numeric(as.character(dfte_m$ID)), # 真实结果（二分类变量）

LR = lrprote, # 逻辑回归预测概率

XGBoost=xgb.prete,

RF = m_rftepro, # 随机森林预测概率

KNN=knntepro,

SVM = msvmprote,# 支持向量机预测概率

NaiveBayes=nbtepro

)

dca_teresults <- dca(

data = dca_datate,

outcome~.,

thresholds = seq(0.01, 0.99, 0.01)

)

\# 绘制DCA曲线

plot(dca_teresults) +

theme_bw() +

labs(title = "Decision Curve Analysis (Male model)",

x = "Threshold probability",

y = "Net Benefit") +

scale_color_manual(values = c("darkgrey","grey","#fed439","#709ae1","#8a9197","#d2af81","#fd7446","#d5e4a2") )+

theme(legend.position = c(0.9,0.8),

panel.grid=element_blank(),

legend.key.size = unit(13, "pt"))

ggsave("C:\\Users\\wlxy8\\Desktop\\课题\\肺结核鉴别诊断\\研二下诊断分析\\sace全重新做\\不平衡数量\\重新插补后的结果\\男DCA验证集3.pdf",width=7,height=7,dpi=300)

####Calibration curve/MALE/train#####

calibration_data <- data.frame(

ID = as.numeric(as.character(dftr_m$ID)),

LR = lrprotr,

XGBoost = xgb.pre,

RF = m_rftrpro,

KNN = cv_probs,

SVM = msvmprotr,

NaiveBayes = nbtrpro

)

library(runway)

library(Hmisc)

create_calibration <- function(data, prob_col, outcome_col, model_name, n_bins = 5) {

data <- data[!is.na(data[[prob_col]]) & !is.na(data[[outcome_col]]), ]

data$bin <- cut2(data[[prob_col]], g = n_bins)

data %>%

group_by(bin) %>%

summarise(

mean_pred = mean(.data[[prob_col]]),

obs_freq = mean(.data[[outcome_col]]),

n = n(),

\# 使用二项式精确置信区间（Clopper-Pearson）

ci_lower = binom.test(sum(.data[[outcome_col]]), n(), conf.level = 0.95)$conf.int[1],

ci_upper = binom.test(sum(.data[[outcome_col]]), n(), conf.level = 0.95)$conf.int[2],

.groups = 'drop'

) %>%

mutate(model = model_name)

}

models <- c("LR", "XGBoost", "RF", "KNN", "SVM", "NaiveBayes")

model_names <- c("LR", "XGBoost", "RF", "KNN", "SVM", "NaiveBayes")

cal_results <- bind_rows(lapply(seq_along(models), function(i) {

create_calibration(calibration_data, models[i], "ID", model_names[i], n_bins = 5)

}))

jzqx1 <- ggplot(cal_results, aes(x = mean_pred, y = obs_freq, color = model)) +

geom_line() +

\# geom_smooth(method = "loess")+

geom_point() +

#geom_errorbar(aes(ymin = ci_lower, ymax = ci_upper), width = 0.02, alpha = 0.5) +

geom_abline(slope = 1, intercept = 0, linetype = "dashed") +

scale_size_continuous(range = c(2, 5)) +

scale_color_manual(values = c("#709ae1","#8a9197","#fed439","#d2af81","#fd7446","#d5e4a2"))+

labs(title = "Calibration Curve (Male model)",

x = "Mean Predicted Probability",

y = "Observed Proportion",

color = "Model"

) +

coord_cartesian(xlim = c(0, 1), ylim = c(0, 1)) +

theme_bw()+

theme(panel.grid = element_blank())

ggsave("C:\\Users\\wlxy8\\Desktop\\课题\\肺结核鉴别诊断\\研二下诊断分析\\sace全重新做\\不平衡数量\\重新插补后的结果\\男校准曲线训练集3.pdf",width=7,height=6,dpi=300)

\# calculate Brier

brier_scores <- data.frame(

Model = models,

BrierScore = sapply(models, function(m) {

mean((calibration_data[[m]] - as.numeric(as.character(calibration_data$ID)))^2)

})

)

\# Brier score

print(brier_scores)

######calibration/ man /validation#########

calibration_data2 <- data.frame(

ID = dfte_m$ID,

LR = lrprote,

XGBoost = xgb.prete,

RF = m_rftepro,

KNN = knntepro,

SVM = msvmprote,

NaiveBayes = nbtepro

)

names(calibration_data2)[1] <- "obs"

cal_long2 <- calibration_data2 %>%

pivot_longer(cols = -obs, names_to = "model", values_to = "pred")

nbins <- 5

cal_curve_data2 <- cal_long2 %>%

group_by(model) %>%

mutate(bin = ntile(pred, nbins)) %>% # 分成nbins组

group_by(model, bin) %>%

summarise(

mean_pred = mean(pred, na.rm = TRUE),

mean_obs = mean(obs, na.rm = TRUE),

.groups = "drop"

)

\# plot

jzqx2 <- ggplot(cal_curve_data2, aes(x = mean_pred, y = mean_obs, color = model)) +

geom_line() +

geom_point() +

geom_abline(intercept = 0, slope = 1, linetype = "dashed", color = "gray") +

scale_color_manual(values = c("#709ae1","#8a9197","#fed439","#d2af81","#fd7446","#d5e4a2"))+

labs(

title = "Calibration Curves (Male Model)",

x = "Mean Predicted Probability",

y = "True Probability"

) +

scale_y_continuous(limits=c(0,1),seq(0,1,0.25))+

theme_bw()+

theme(panel.grid = element_blank())+

coord_equal() # 使x和y轴比例相同，更易看出偏差

ggsave("C:\\Users\\wlxy8\\Desktop\\课题\\肺结核鉴别诊断\\研二下诊断分析\\sace全重新做\\不平衡数量\\重新插补后的结果\\男校准曲线验证集2.pdf",width=7,height=8,dpi=300)

\# Brier score

brier_scores2 <- data.frame(

Model = models,

BrierScore = sapply(models, function(m) {

mean((calibration_data2[[m]] - as.numeric(as.character(calibration_data2$obs)))^2)

})

)

#####Male/SHAP########

library(e1071)

library(kernelshap)

library(shapviz)

library(ggplot2)

positive_class <- levels(factor(dfte_m$ID))[2] # 假设 "1" 是正类

predict_prob_svm <- function(object, newdata) {

pred <- predict(object, newdata = newdata, probability = TRUE)

prob <- attr(pred, "probabilities")[, positive_class]

return(prob)

}

set.seed(123)

Xtest <- dfte_m[, features]	# features

n_bg <- min(200, nrow(Xtest))

bg_idx <- sample(1:nrow(Xtest), n_bg)

bg <- Xtest[bg_idx, ]

X_explain <- Xtest

shap <- kernelshap(

object = m_svmmod,

X = X_explain,

bg_X = bg,

pred_fun = predict_prob_svm,

tol = 0.01,

max_iter = 250,

parallel = TRUE

)

\# 转换为 shapviz 对象，便于绘图

shv <- shapviz(shap, X = X_explain)

sv_waterfall(shv, row_id =6,max_display = Inf) +

#ggtitle("Waterfall Plot for First Sample") +

theme_bw()+

theme(panel.grid=element_blank())

ggsave("C:\\Users\\wlxy8\\Desktop\\课题\\肺结核鉴别诊断\\研二下诊断分析\\sace全重新做\\不平衡数量\\重新插补后的结果\\shap男瀑布图3.pdf",width=6,height=5,dpi=300)

sv_importance(shv, kind = "bar") + # 'bee'显示散点，'bar'为纯柱状

#ggtitle("SHAP Variable Importance") +

theme_bw()+

theme(panel.grid=element_blank())

ggsave("C:\\Users\\wlxy8\\Desktop\\课题\\肺结核鉴别诊断\\研二下诊断分析\\sace全重新做\\不平衡数量\\重新插补后的结果\\shap男重要性图3.pdf",width=6,height=5,dpi=300)

sv_importance(shv, kind = "bee") + # 'bee'显示散点，'bar'为纯柱状

#ggtitle("SHAP Variable Importance") +

theme_bw()+

theme(panel.grid=element_blank())

ggsave("C:\\Users\\wlxy8\\Desktop\\课题\\肺结核鉴别诊断\\研二下诊断分析\\sace全重新做\\不平衡数量\\重新插补后的结果\\shap男蜂窝图3.pdf",width=6,height=5,dpi=300)

sv_force(shv, row_id = 6)

ggsave("C:\\Users\\wlxy8\\Desktop\\课题\\肺结核鉴别诊断\\研二下诊断分析\\sace全重新做\\不平衡数量\\重新插补后的结果\\shap男单个力图3.pdf",width=7,height=2,dpi=300)

#Dependency graph

dep_plots <- lapply(features, function(feat) {

sv_dependence(shv, v = feat, alpha = 0.5) +

ggtitle(feat) +

theme_minimal() +

theme(plot.title = element_text(size = 10)

)

})

combined_plot <- wrap_plots(dep_plots, ncol = 3)

pdf("C:\\Users\\wlxy8\\Desktop\\课题\\肺结核鉴别诊断\\研二下诊断分析\\sace全重新做\\不平衡数量\\重新插补后的结果\\shap男依赖图3.pdf",

width = 10, height = 9)

print(combined_plot)

dev.off()
